# Cards Against Pulmonology

**DOI:** 10.5070/M5.52358

**Published:** 2026-01-31

**Authors:** Lauren Lamparter, Alisa Wray

**Affiliations:** * University of California, Irvine, Department of Emergency Medicine, Orange, CA

## Abstract

**Audience:**

This card game is designed to cultivate educational discussion among emergency medicine resident physicians about the assessment, treatment, and disposition of key pediatric and adult thoracic-respiratory diagnoses in a fun, casual environment. It could also be played by emergency medicine-bound medical students.

**Introduction:**

Emergency department visits related to the thoracic-respiratory system are common complaints in both the pediatric and adult populations. In the United States, for several years prior to the Covid-19 pandemic, respiratory system diseases accounted for about 10.6% of ED visits.^1^ In children, respiratory complaints make up the largest percentage of their ED visits, particularly in the fall and winter seasons.^2^ This number appears to have only grown higher in both adults and pediatrics in the years following the Covid-19 pandemic.^3^ Thoracic-respiratory disorders also account for about 7% of the American Board of Emergency Medicine In-Training Exam and qualifying exam content.^4^ Therefore, it is paramount that resident physicians understand the presentation, management, and treatment of a wide range of both pediatric and adult thoracic-respiratory complaints and pathology that mimics these presentations. This game explores key topics in the thoracic-respiratory system in both the pediatric and adult populations allowing for fun discussion regarding management, treatment, and disposition of these complicated disease processes. Topics range from sick to not sick patients and include bronchiolitis, pulmonary edema, pulmonary embolism, COPD exacerbation, neonatal cyanosis, viral upper respiratory infections, and more.

**Educational Objectives:**

By the end of this card game, learners will 1) understand the methods of clinical assessment in thoracic-respiratory related diseases, 2) implement escalating levels of respiratory support for thoracic-respiratory pathology in pediatric and adult patients, 3) review and utilize important medications in the management of thoracic-respiratory diseases, and 4) choose appropriate dispositions of patients with various thoracic-respiratory related complaints.

**Educational Methods:**

The goal of Cards Against Pulmonology is for learners to further understand the clinical assessment, management, and disposition of various thoracic-respiratory emergencies by providing the next best critical action in a given clinical situation. This game will equip residents to differentiate the sick from non-sick patients and collaboratively discuss the management and disposition of patients with a variety of thoracic-respiratory related complaints.

This card game is a cognitive artifact designed to stimulate small group discussion that will enhance the clinical reasoning skills of the medical students and resident physicians who play the game. The clinical content of thoracic-respiratory conditions has been gamified through the strategy of play modeled after the popular card game, Cards Against Humanity. Discussion of key educational points during and after the game provides clarification of learner knowledge to solidify concepts.

**Research Methods:**

The game was implemented in a weekly resident educational conference session where 19 resident physicians and several faculty physicians participated in gameplay and immediately following the game, evaluated the educational experience by survey using a Likert scale. They assessed their overall experience with the game, engagement with the game, the game’s ability to reinforce existing medical knowledge, and if game content was relevant to their clinical practice.

**Results:**

The results were overwhelmingly positive with an average of strongly agreed on every Likert scale in every category and a request for the creation of other similar games covering more topics. Resident physicians stated they appreciated being able to laugh and learn, and that the inclusion of case discussion after a case concluded really emphasized the educational points regarding the medical care of patients with respiratory complaints. They encouraged increased discussion of the medicine after each round.

**Discussion:**

Overall, this game was very effective in stimulating conversation regarding the care of patients with thoracic-respiratory related complaints. All medical students, residents, and attending physicians were very engaged and remained excited throughout gameplay. Implementation of the game showed the appropriate small group size is about five to six players to allow for robust discussion and engagement. It is also important for the facilitators to discuss expected outcomes for the patient at the conclusion of a set of case cards to encourage educational value alongside humorous game play.

**Topics:**

Pulmonology, thoracic-respiratory system, shortness of breath, cough, viral respiratory infection, bronchiolitis, asthma, COPD, pulmonary edema, pediatric respiratory conditions.

## USER GUIDE

**Table:** 

List of Resources:	
Abstract	59
User Guide	61
Cards Against Pulmonology Small Group Instructions	63
Instructor Guide and Debrief Pearls	68

**Learner Audience:**

This educational activity is appropriate for medical students, interns, junior and senior residents, and attending physicians.

**Time Required for Implementation:**

45 minutes

**Recommended Number of Learners Per Instructor:**

5–6 learners in each small group, preferably a mix of residency years

**Topics:**

Pulmonology, thoracic-respiratory system, shortness of breath, cough, viral respiratory infection, bronchiolitis, asthma, COPD, pulmonary edema, pediatric respiratory conditions.

**Objectives:**

By the end of this card game, learners will:

1. Understand the methods of clinical assessment in thoracic-respiratory related diseases
2. Implement escalating levels of respiratory support for thoracic-respiratory pathology in pediatric and adult patients
3. Review and utilize important medications in the management of thoracic-respiratory diseases
4. Choose appropriate dispositions of patients with various thoracic-respiratory related complaints

### Linked objectives and methods

The goals and objectives of this gamified card game are achieved through collaborative discussion of the thoracic-respiratory content using the cognitive artifact of patient case, assessment, intervention, and disposition cards. While allowing for humorous release and psychological safety, learners engage with real life cases of varying severity and follow along as their actions have cause and effect results for the patients. At completion of each round of a case, the learners explain why they chose the card they played, and the team discusses alternatives and correct assessment, intervention, and disposition, and the faculty correct any knowledge gaps. This allows resident physicians to further understand the appropriate assessment, management, and disposition for patients with various thoracic-respiratory complaints, allowing them to practice, integrate knowledge, and build future knowledge to more appropriately take care of patients with thoracic-respiratory related illnesses. All four objectives are built into each step of play. The instructions and rules of the card game are attached below.

### Recommended pre-reading for facilitator

Game instructions and Case Instructor Guide linked below. Any article on thoracic-respiratory diseases and care in emergency medicine.

### Results and Tips for Successful Implementation

The game was implemented in a weekly resident educational conference and played by medical students, resident physicians, and attending physicians. Our total residency size is 27 residents, and 19 players participated in gameplay. Immediately following the game, they evaluated the educational experience by survey using a Likert scale. They assessed their overall experience with the game, engagement with the game, the game’s ability to reinforce existing medical knowledge, and if game content was relevant to their clinical practice.

The results were overwhelmingly positive with almost unanimous rating of “strongly agree” in each category: 100% stated they strongly agreed their experience with the game was positive, 100% strongly agreed the game was engaging, 94% strongly agreed the game reinforced their medical knowledge (6% stating they agree), and 100% strongly agreed that the game was relevant to their clinical practice. The residents unanimously requested similar games in the future with one requesting an adaptation to critical care cards. Comments included, “This is a must do - provides a safe space to laugh and joke while also learning!” and “This was very fun! I loved how it was fun and silly but also educational!” The importance of psychological safety in the learning environment did not go unrecognized by the residents, and they expressed that the inclusion of immediate case discussion after case set concluded really emphasized the educational points regarding the medical care of patients with thoracic-respiratory complaints.

Overall, this game was very effective in stimulating conversation regarding the care of patients with thoracic-respiratory system related complaints. All residents were very engaged and remained excited throughout the gameplay. Implementation of the game showed the appropriate small group size is about five to six players to allow for robust discussion and engagement. It is also important for the facilitators to discuss expected outcomes for the patients at the conclusion of a set of case cards to encourage educational value alongside humorous game play. Suggested discussion points for each case are included on the Case Instructor Guide below.

## Supplementary Information



## Figures and Tables

**Figure:**
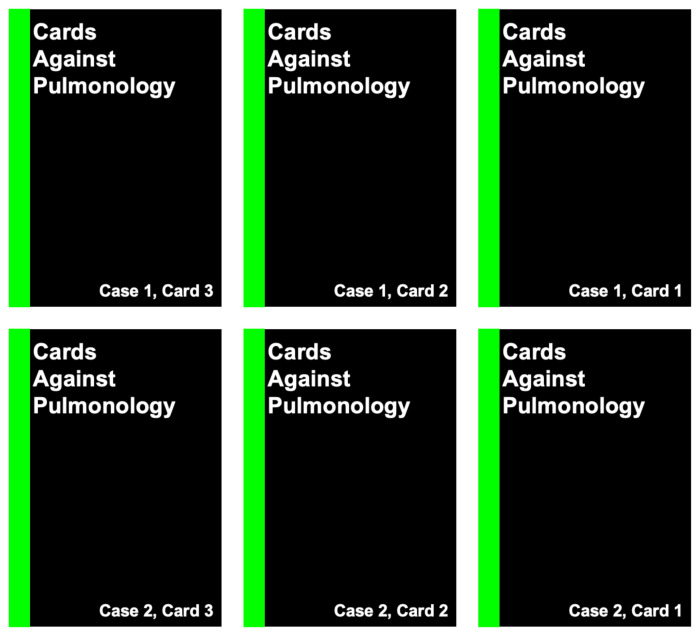
Please see associated PowerPoint file

## SMALL GROUPS LEARNING MATERIALS

### Cards Against Pulmonology Small Group Instructions

**Number of players:** Ideally about 5–6 players per group, one facilitator.

- Senior residents may be facilitators to debrief game play while also engaging in the game.

**Cards:**

- Clinical Scenario Cards
  ○ Labeled Case 1, 1–1, Case 1, 1–2, etc.
  ○ Played in order during rounds of game play to advance the case
- Action Cards
  ○ Assessment Cards
  ○ Disposition Cards
  ○ Intervention Cards

**Game Play**

- Faculty or senior residents will facilitate the game (Facilitator). They will hold the Case Scenario Cards and play them to advance the case and debrief at each stage.
- Each learner playing (Players) will need to draw 6 action cards:
  ○ **2** Assessment cards
  ○ **1** Disposition card
  ○ **3** Intervention cards
- Players should ***always have 6 action cards*** (2 Assessment, 1 Disposition, 3 Intervention) in their hand. Cards are color-coded (green case cards, pink assessment cards, blue disposition cards, and purple intervention cards) for ease. After a card is played, players will draw a new action card from the category they play from in a round to replenish the same number of action cards in their hand. Players will always have 6 action cards in their hand (2 assessment, 1 disposition card, 3 intervention cards).
- Playing a Round:
- For each round the Facilitator will hand a **clinical scenario card** to one of the Players who will serve as that round’s judge.
  ○ Clinical Scenario Cards will be played in order as labeled to progress through a full Case (labeled Case 1, 1-x). Cases are worded similar in the structure to the questions in a standardized interview oral board case.
    ▪ For example, Case 1, 1–1 will be played first, followed by Case 1, 1–2, followed by Case 1, 1–3, etc., until all Case cards have been played.
    ▪ Then the Facilitator will briefly discuss the educational points of the case, and choose the next Case set to play.
  ○ Cases are organized in progression, so the natural next card is drawn relative to the same clinical situation, ie., treating respiratory syncytial virus (RSV).
- The round’s judge will read the Clinical Scenario Card, and other game players will all submit an action card (assessment, intervention, or disposition) to fit the scenario, **answering the question: what is the best next clinical step?** Action cards should be submitted face side down. The judge will not submit an action card.
  ○ As the cards are shuffled and distributed randomly, there may not be a “correct” answer for a player to submit. Players must choose any action card from their hand to play for that round regardless of if it is medically “correct.” This allows for some lighthearted play as several of the action cards are designed to be incorrect and allow for comedic relief.
- When all action cards are submitted, the judge will turn them over and read them one at a time. The judge will then select the best action card for that round (this game allows the answers to be humorous or serious, depending on the judge who is assessing the submitted answer cards).
  ○ The judge selects the “winning” action card, and the player who submitted that action card receives the clinical scenario card, ending that round.
  ○ If the medically correct answer is not chosen or not available to choose, and the judge chooses a humorous approach, the correct medicine will be discussed by the group facilitator when play for that set of case cards concludes.
- Moving clockwise, the next player will be handed the next clinical scenario card in the case and become the judge, repeating the process.
  ○ The facilitators may still engage in game play, being the judge when it is their respective turn.
- When the final Clinical Scenario Card in a Case has been played, the round is complete. Prior to moving onto the next case, the facilitator will give a brief review of appropriate assessment, management, and disposition.
  ○ Given that the game does not always allow the patient to play the correct answer card by nature of randomization, facilitator should ask the group what the correct next step would be for each component of the case before revealing the correct answers to assess knowledge.
  ○ Facilitators should then answer any questions from the learners and correct any misconceptions.
  ○ The game will then continue to the next case.
- The first player to collect **5 clinical scenario cards**, wins that game.
  ○ The game will continue for as many rounds as it takes for the winning player to collect 5 clinical scenario cards.
  ○ If a player has not reached 5 scenario cards by the time the scheduled educational session ends, the player with the most clinical scenario cards wins.
  ○ Consider providing a small prize to the player who collects 5 scenario cards first.

**Special Situation Instructions:**

- If two players play the same action card, AND that action is selected as the **best next patient outcome** by that round’s judge, the next clinical scenario card will be drawn by the same judge for game play by ONLY the two tied players.
  ○ The tied players will each play a second action card. The judge will select the best next outcome for that clinical scenario card.
  ○ The winner of the tie breaker will win BOTH clinical scenario cards; then the game will progress as normal

**Making The Cards:**

- Please see attached PowerPoint document of clinical scenario cards, assessment cards, intervention cards, and management cards.
- Cards were created on PowerPoint using a playing card template and then double-side printed using printable playing card paper with pre-perforated cards. However, they could easily be printed on cardstock and cut down to size. They were then thermal laminated to allow for repeated use.
  ○ Pre-cut perforated cards: https://www.amazon.com/White-Printable-Playing-Card-Paper/dp/B0D6T42NZ9/ref=sr_1_8?crid=FIS3LM3E9DOR&dib=eyJ2IjoiMSJ9.SQVNWBIoMR76AaeMZMtzyY1VGfIPagOGBszjwvVL_AWf34L0t5TCoeXwZIzAC1y153jPXzsSZRssXhTWOYOaLsmAHRSuDPo5PXy8F17PNqBB7sv5bTy3JXFE88xkUG1QGQquwxj80BJASvG9rraPz-WiDa5NifxnfUbrEXuoJFFW3Ohr2n9oc3g9ZGj30ria1QqQqyuODXTvQU9COsTQwxywNbvsKTLkDL1ew114SPiQ5Ev1EtJsExGZ_OQLdcRpx3E4rXwq-37a3FM6jdVqZvnHuEKJU0d4Tfig4AvV6nI.NydG4MsFLtv3sIKvtdFEl6BYKuUTLyRgFt7df1IWhY8&dib_tag=se&keywords=printable+playing+cards&qid=1747673621&sprefix=printable+playing+card%2Caps%2C191&sr=8-8
  ○ Thermal Lamination Pouches: https://www.amazon.com/Wallet-Photo-Laminating-Pouches-Clear/dp/B00KOCJCKI/ref=sr_1_6?crid=BVKA2BB4VV9L&dib=eyJ2IjoiMSJ9.gsNTqBVFErNrqxbUhaAP3KZ8eqnuWXrZjXmCfRULHQJ6QW2XJASgRzh5V7SdsF6HEVI2A7aBbupUJyZsrYum2uP5Wyn_dWLKTnth6iVr6KG2FurXn7hR-29FZNpeVPGAffU6lfLdAHB3aWY8LbsOFOQJU3sj-cjXE6KUcq5QSdYurVrM5XqC-TA1y_gkXaR-zsCs1c-mr0hhgnb4IzbFeuK-0BviT1QXHlofFuGWFkR-VQen7q6l77kh2ym0wiybixfQfhBajso3IL8UB3WN23n9jslyVkH8ElOfE67R9_4.a3dQ8V8jKxZcC6GkS9QXb7F9e-tyzrK-sJJgWUBJxOM&dib_tag=se&keywords=2+3%2F4+by+3+3%2F4+lamination+pouches&qid=1747673992&sprefix=2+3%2F4+by+3+3%2F4+lamination+pouches%2Caps%2C159&sr=8-6
    or
  ○ Self-Sealing Laminating Pouches: https://www.amazon.com/IMAGAME-Self-Sealing-Laminating-Lamination-Waterproof/dp/B0D3DM229P/ref=sr_1_3?crid=2TT9F8890JXOZ&dib=eyJ2IjoiMSJ9.SEmsMwKiPHhBtGiSUbkp6HInN7Uy1FPxL5LVblyNU5vKamBft2-U0cJ3Hmv_HFofoNdsT9Kt7yM4oehHvNguXeFNDmUvL896wW0Oy64J_MTj2_orOz4PKy8-VEMH0gJiJgcsPSOZSFulCn_TXnYrcUpV-Q00xN3Dj858wYi_VzYbxo7ZQuIkgDGaZpS8W_L_JYkSRaB3atrVFWAGZKyyuJg_BOXinbJNtYpP_elgfCg.JODwJdGIzhL_LvSDquWxSn8sKln6oO3fzs11MAvmFds&dib_tag=se&keywords=playing%2Bcard%2Bself%2Bsealing%2Blaminating%2Bpouches&qid=1752864615&sprefix=playing%2Bcard%2Bself%2Bsealing%2Blaminating%2Bpouches%2Caps%2C156&sr=8-3&th=1

**Card Content:**

1. Clinical Scenario Cards:
  - Clinical Scenario Cards are ordered Case 1, Card 1… Case 2, Card 2…. These cards are designed to be played sequentially. As the instructor, you should mix up the case order (Case 1, Case 7, Case 3, etc.) while keeping the cards related to each case in sequential order. Provide the player who is judging a given round with the next sequential case card after every round to ensure they are in order.
  - Provide a brief clinical review at the conclusion of each set of cards in a case.
2. Clinical Action Cards:
  - Within the deck, there are 5 clinical action cards for every one case card.
  - If you are playing with more than 5 people, shuffle the discarded action cards when you run low on the specific type of cards throughout game play. Many action cards can be used several times.
  - The action cards include assessment cards, intervention cards, and disposition cards.

**Making the cards your own:**

- Some of the cards are specific to our emergency department. Please feel free to edit cards that include interventions or assessments that are not possible in your department.
- It can be fun to add flair to things that happen in your department. Please feel free to edit cards to have some language unique to your institution and learners.
- Some of the cards are intended for comedic relief rather than medical accuracy. Because of this, we encourage the facilitator to discuss the clinical relevance of each case following case conclusion.

### Instructor Guide and Debrief Pearls

The following instructor guide references each set of case cards with discussion points intended to be discussed with the players after a case is completed. The content of the case cards and action cards (assessment, management, disposition) are not listed here but can be found in the card game template (separate power-point).

#### Case 1: Bronchiolitis^5^

- Bronchiolitis occurs most commonly in children with upper respiratory illnesses caused by respiratory syncytial virus, but can be caused by other viruses.
- The best next step for these children is often quality nasal suctioning to relieve the nasal passages from congestion.
- Interventions such as albuterol, nebulized saline, and steroids have not shown clinical benefit in bronchiolitis.
- The disposition of a child with bronchiolitis will depend on their work of breathing and oxygen saturation. With increased work of breathing and any O2 requirement, these children will need to be admitted. High flow nasal cannula is an excellent choice for these children because the humidified oxygen helps to improve respiratory effort and has been proven to decrease the rate of intubation in children with bronchiolitis.

#### Case 2: Croup (moderate to severe)^6^

- Croup presents with a barky cough, sometimes stridor, and is often worse at night and is a clinical diagnosis.
- Croup severity is assessed as mild/moderate/severe with stepwise interventions depending on the child’s assessed severity.
  ○ mild = no stridor or significant retractions at rest
  ○ moderate= stridor and chest wall retractions at rest
  ○ severe= stridor, chest wall retractions, and agitation or lethargy
- Distinguish between: tracheitis, epiglottis, foreign body aspiration, peritonsillar abscesses or retropharyngeal abscesses, allergic reactions, among other differential diagnoses.
- Treatment of all stages of croup is with steroids (Decadron 0.6mg/kg one time dose, IV formulation given PO).
- Treatment of moderate to severe croup includes the addition of racemic epinephrine and warrants an observation period of 2–3 hours after administration to insure no rebound .
- If there is no rebound response, the child can be discharged home with strict return precautions. If there was a rebound requiring repeated doses of racemic epinephrine, admission must be considered with an assessment of severity of illness for general ward vs. PICU admission.

#### Case 3: Croup (mild)^6^

- See above (Case 2-moderate/severe Croup)
- This child does not need racemic epinephrine and can be safely discharged home with strict return precautions.

#### Case 4: COPD exacerbation^7^–^8^

- This patient has COPD and is in respiratory distress.
- The next step on initial management is to place this man in a bed, administer oxygen, and escalate quickly to BiPAP (Bilevel-positive airway pressure).
- The standard management of an acute COPD exacerbation is with oxygen, inhaled bronchodilators, and corticosteroids. Antibiotics such as macrolides can be considered because they have anti-inflammatory and immune-modulating effects leading to decreased airway inflammation.
- Magnesium 2g IV over 20 minutes can also be administered to assist with bronchodilation, and it has been shown to decrease hospital admission and length of stay.
- Learners should recognize this patient is very sick and administer continuous albuterol, methylprednisolone, and start him on BiPAP. The leg swelling is an acknowledgement that this patient has such severe lung disease, he may now have a component of right heart failure (learners should distinguish between COPD and congestive heart failure (CHF) exacerbation).
- This case is designed to have various escalations depending on what intervention is chosen, and you as the facilitator should discuss with the learners the various paths the patient could have gone down and why a multimodal approach of management is key in real-life clinical scenarios:
  ○ Depending on the previous intervention played, resident will read the scenario:
    ▪ 1) *If high flow or Bipap initiated* - patient’s respiratory rate improves, and he sits forward, breathing with the rate of the vent, taking deep breaths. You can hear improved air movement, but the wheezing persists.
    ▪ 2) *If Douneb or steroids started* - patient will be air hungry, respiratory rate remains elevated, still clutching his chest and states the medicine isn’t enough, “Can you help me more, doc?”
    ▪ 3) *If any other intervention was selected*, patient will become unresponsive and new vitals are HR 75 BP 85/50, RR 4, 72% on 6L nasal cannula.
    ▪ This patient will need to be admitted to the ICU.

#### Case 5: Postpartum Pulmonary Edema from Cardiomyopathy^9^

- Postpartum patients are at risk for developing cardiomyopathy in the weeks leading to delivery and up to five months postpartum.
- They are also at risk for postpartum preeclampsia, and in this context, the patient’s dyspnea and hypertension should prompt learners to start antihypertensive medications and magnesium empirically while more assessment is being performed. The immediate best next step is to move this patient to a treatment space, on a monitor.
- The treatment of pulmonary edema from postpartum cardiomyopathy is the same as treating CHF: vasodilation, positive pressure ventilation, and diuresis. Learners should control blood pressure with nitroglycerin and start the patient on Lasix, and consider starting BiPAP. The patient will need to be admitted and monitored for arrhythmia. A discussion of initiating anticoagulation given high risk of thromboembolic events should be done with the OBGYN team.

#### Case 6: Upper respiratory illness^10^

- This patient has a viral respiratory illness and needs no further workup or management.
- Discuss over-the-counter medications for management of viral upper respiratory infection (URI).
- Counsel this patient against a CXR as he does not have evidence of pneumonia. As a further test, you can get a Covid/Flu swab, but it may not give the patient the answer. Treatment plan should focus on anticipatory guidance and supportive care.

#### Case 7: Hemoptysis^11^–^12^

- The most common cause of hemoptysis presenting to the ED is bronchitis. Other differential diagnoses include pulmonary embolism (PE), TB, pneumonia, or lung cancer.
- This patient was appropriately placed in isolation with concern for infectious processes but then found to have cancer. The moderate volume hemoptysis indicates the hemoglobin is likely lower than calculated on the given CBC.
- It is most important to assess the patient from a respiratory standpoint, rather than from the hemoglobin.
- This patient needs to be admitted and closely monitored for airway deterioration and a need for blood transfusion.

#### Case 8: Massive Pulmonary Embolism^13^–^14^

- For the first card, the STEMI is meant to be a distractor. The residents should prepare a room for the patient and obtain a repeat EKG. Trust but verify STEMIs called in the field, and always repeat an EKG on arrival to the ED.
- When you assess the patient in card 2, she is hypoxic and needs to be intubated. This should prompt a discussion about the difficulty of intubating someone with a massive pulmonary embolism secondary to rapid desaturation despite pre-oxygenation and the shift in intrathoracic pressure caused by intubation that will likely lead to cardiac arrest. The patient suffers a cardiac arrest after intubation because of the positive pressure adding to the strain on the heart from the PE. The treatment is IV thrombolytics, and you can give either Tissue-type plasminogen activator (TPA) (50 mg bolus over 2 minutes, with possible repeat in 10–30 minutes) or Tenecteplase (TNK) (no accepted dose, but consider 25 mg-50 mg bolus, depending on patient’s weight). Cardiopulmonary resuscitation needs to continue for a minimum of 30 minutes to an hour after starting IV thrombolytics.

#### Case 9: CHF exacerbation^15^–^16^

- The next step is to perform a good physical exam and assess his stability.
- Treating methamphetamine induced cardiomyopathy is the same as treating CHF; treatment should include blood pressure control and diuresis with nitroglycerin and furosemide. At this point in time, he is stable and does not need positive pressure ventilation (BiPAP).
  If he decompensates further, consider beginning positive pressure ventilation with BiPAP. An argument could be made for nasal cannula vs. high flow. He will need to be admitted.

#### Case 10: Neonatal Cardiomyopathy^17^–^18^

- Difficulty feeding or sweats with feeds should raise your suspicion for a pediatric congenital cardiac abnormality because it is a type of baby stress test indicating neonatal heart failure. Consider these conditions with feeding difficulties and neonatal cyanosis.
- This child has evidence of a cardiac abnormality and heart failure by the history of sweating with feeds. At this young age, we have to presume the etiology is closing of the patent ductus arteriosus (PDA) which is required to allow circulation of oxygenated blood in many congenital cardiac conditions. When the PDA closes, these children experience significant problems manifested by cyanosis. To keep the ductus arteriosus open, treat the neonate with prostaglandin E.
- The child should be started on oxygen, continuous monitoring, and empiric prostaglandin E while the etiology is being sought with EKG, chest x-ray, and echocardiogram.
  The child will need to be admitted for further evaluation and medical optimization with likely surgical management, depending on the cardiac lesion identified.
