# Supplementary material for: Cards Against Pulmonology
Source: J Educ Teach Emerg Med. 2026 Jan 31;11(1):SG59–72. doi: 10.5070/M5.52358 (PMC12880885; doi:10.5070/M5.52358)
Supplement: Supplementary file 1 [file 11-1-SG59-Supp.pptx]

## Slide 1
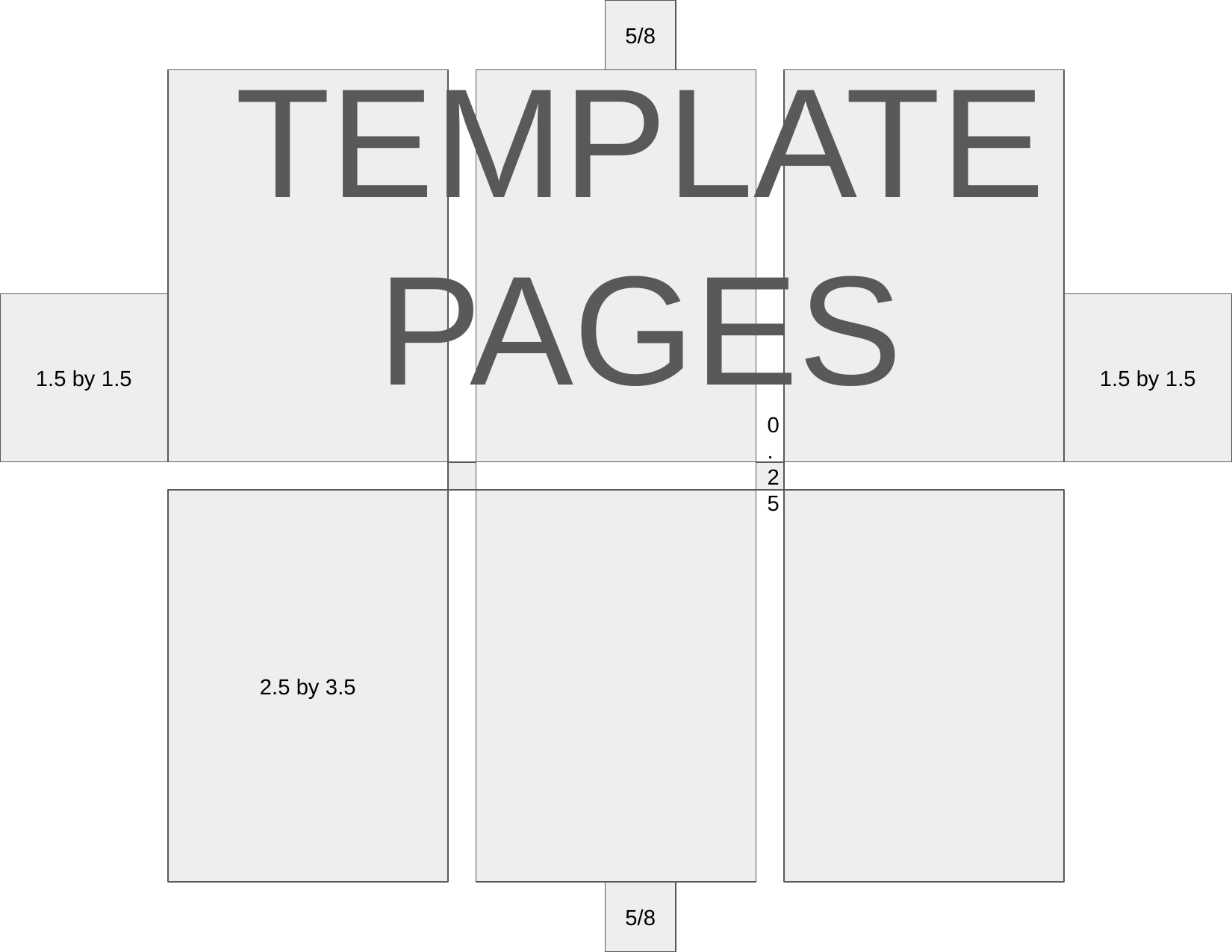

5/8
TEMPLATE PAGES
1.5 by 1.5
1.5 by 1.5
0.25
2.5 by 3.5
5/8

## Slide 2
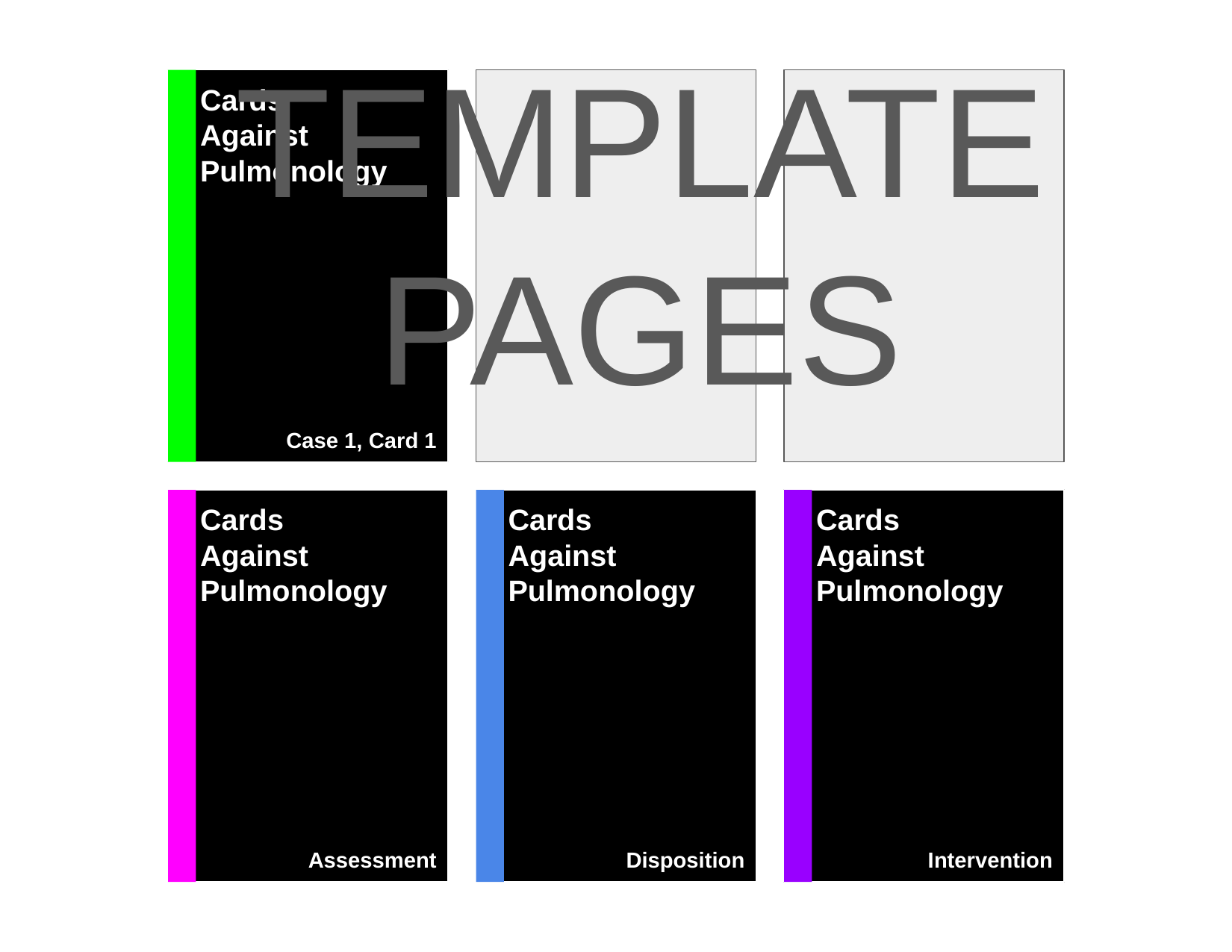

TEMPLATE PAGES
Cards
Against
Pulmonology
Case 1, Card 1
Cards
Against
Pulmonology
Assessment
Cards
Against
Pulmonology
Disposition
Cards
Against
Pulmonology
Intervention

## Slide 3
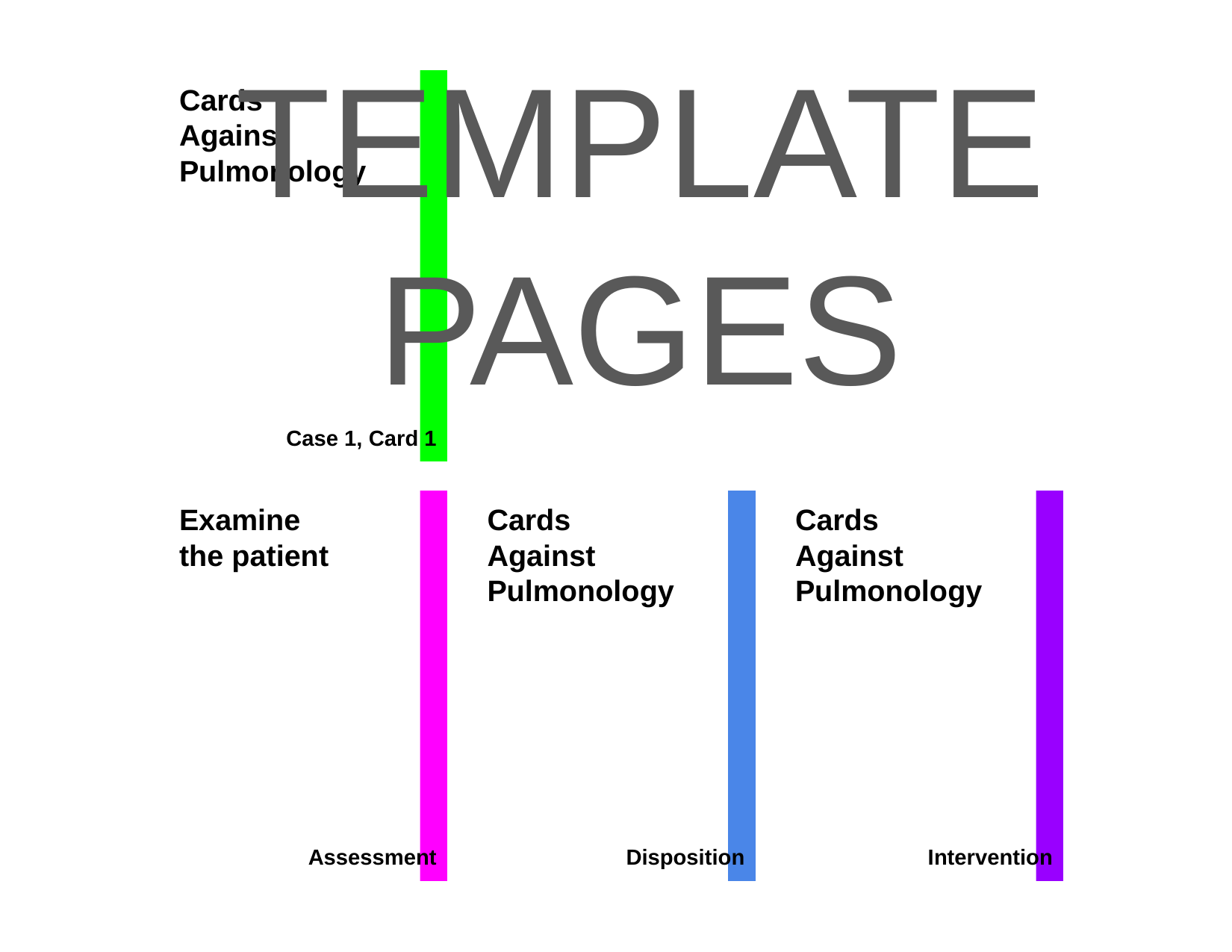

TEMPLATE PAGES
Cards
Against
Pulmonology
Case 1, Card 1
Examine
the patient
Assessment
Cards
Against
Pulmonology
Disposition
Cards
Against
Pulmonology
Intervention

## Slide 4
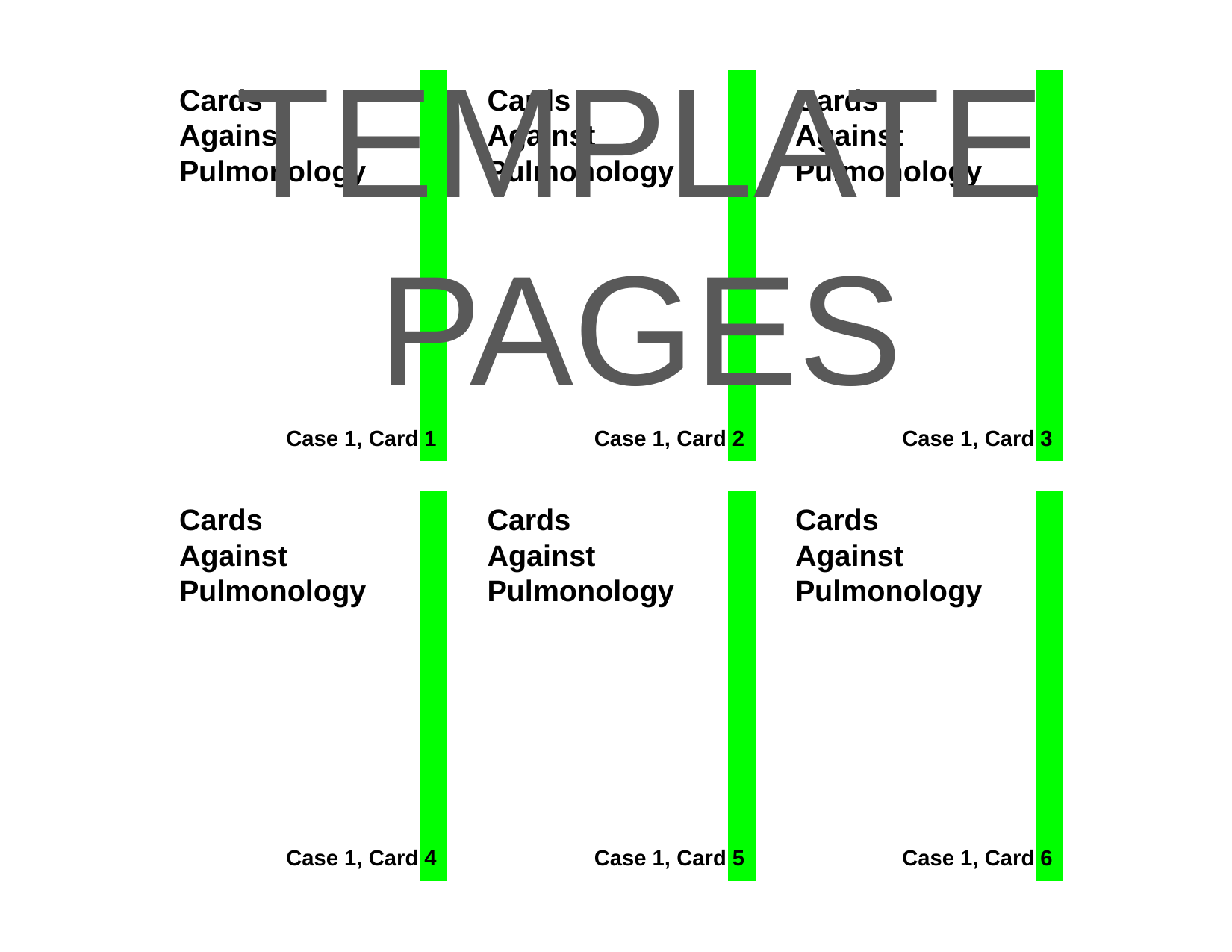

TEMPLATE PAGES
Cards
Against
Pulmonology
Case 1, Card 1
Cards
Against
Pulmonology
Case 1, Card 2
Cards
Against
Pulmonology
Case 1, Card 3
Cards
Against
Pulmonology
Case 1, Card 4
Cards
Against
Pulmonology
Case 1, Card 5
Cards
Against
Pulmonology
Case 1, Card 6

## Slide 5
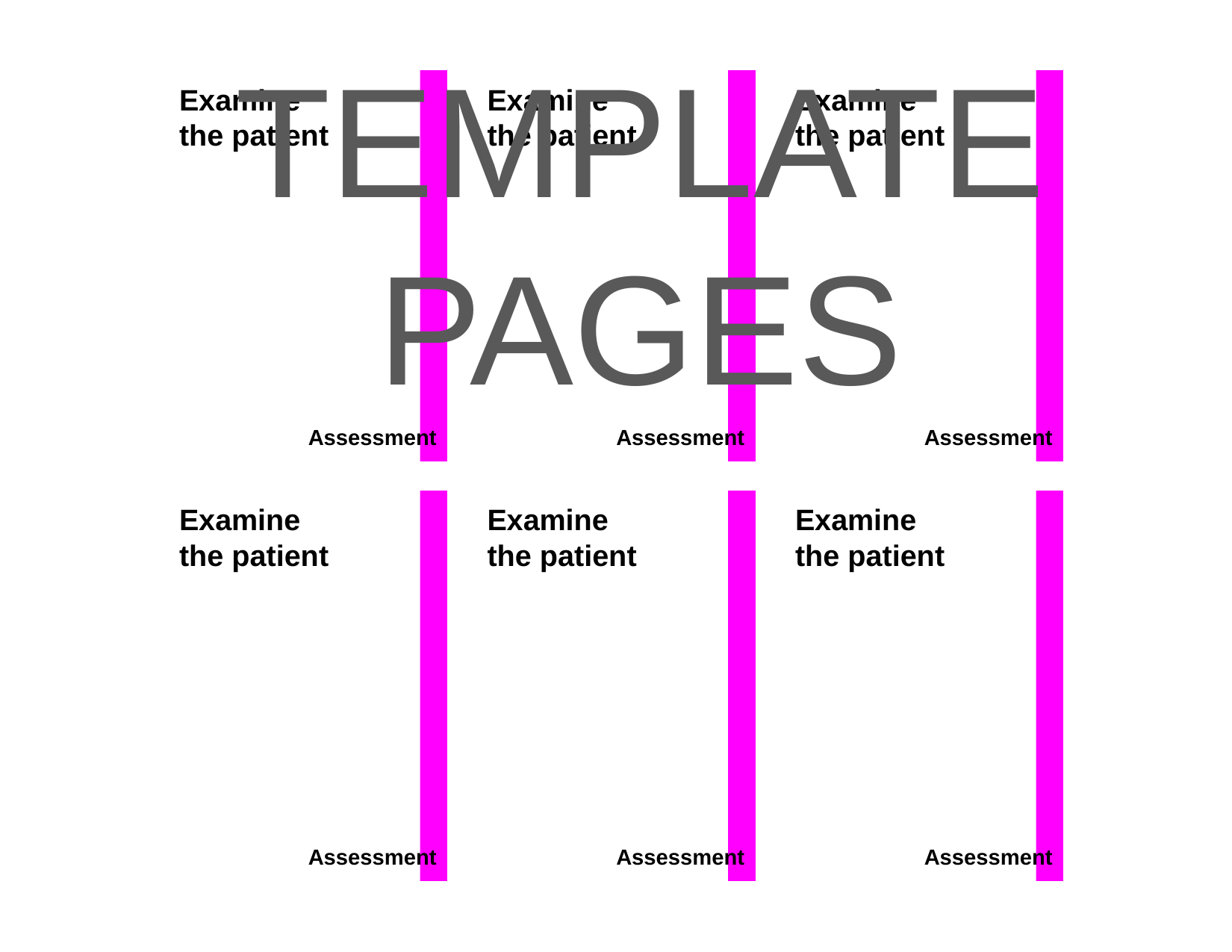

TEMPLATE PAGES
Examine
the patient
Assessment
Examine
the patient
Assessment
Examine
the patient
Assessment
Examine
the patient
Assessment
Examine
the patient
Assessment
Examine
the patient
Assessment

## Slide 6
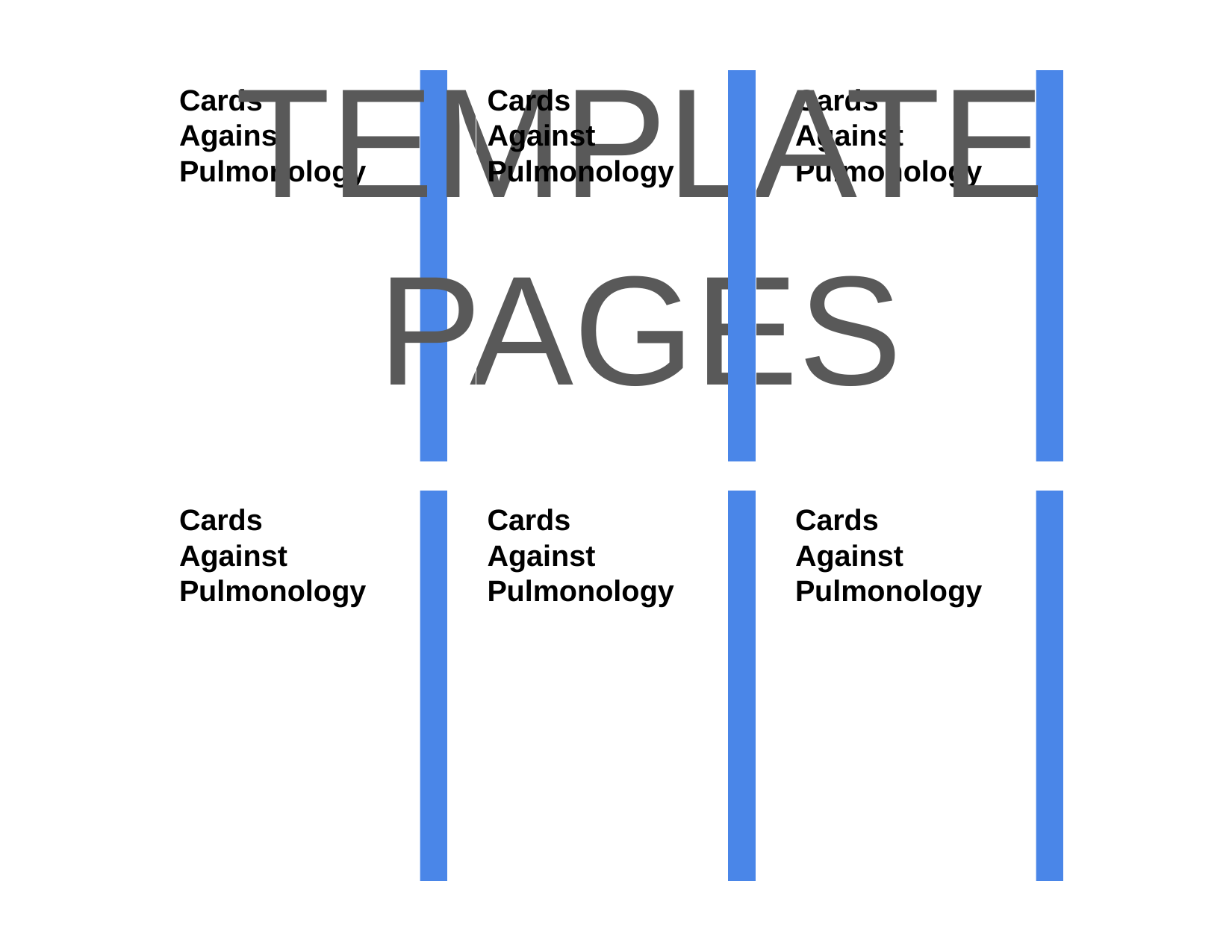

TEMPLATE PAGES
Cards
Against
Pulmonology
Cards
Against
Pulmonology
Cards
Against
Pulmonology
Cards
Against
Pulmonology
Cards
Against
Pulmonology
Cards
Against
Pulmonology

## Slide 7
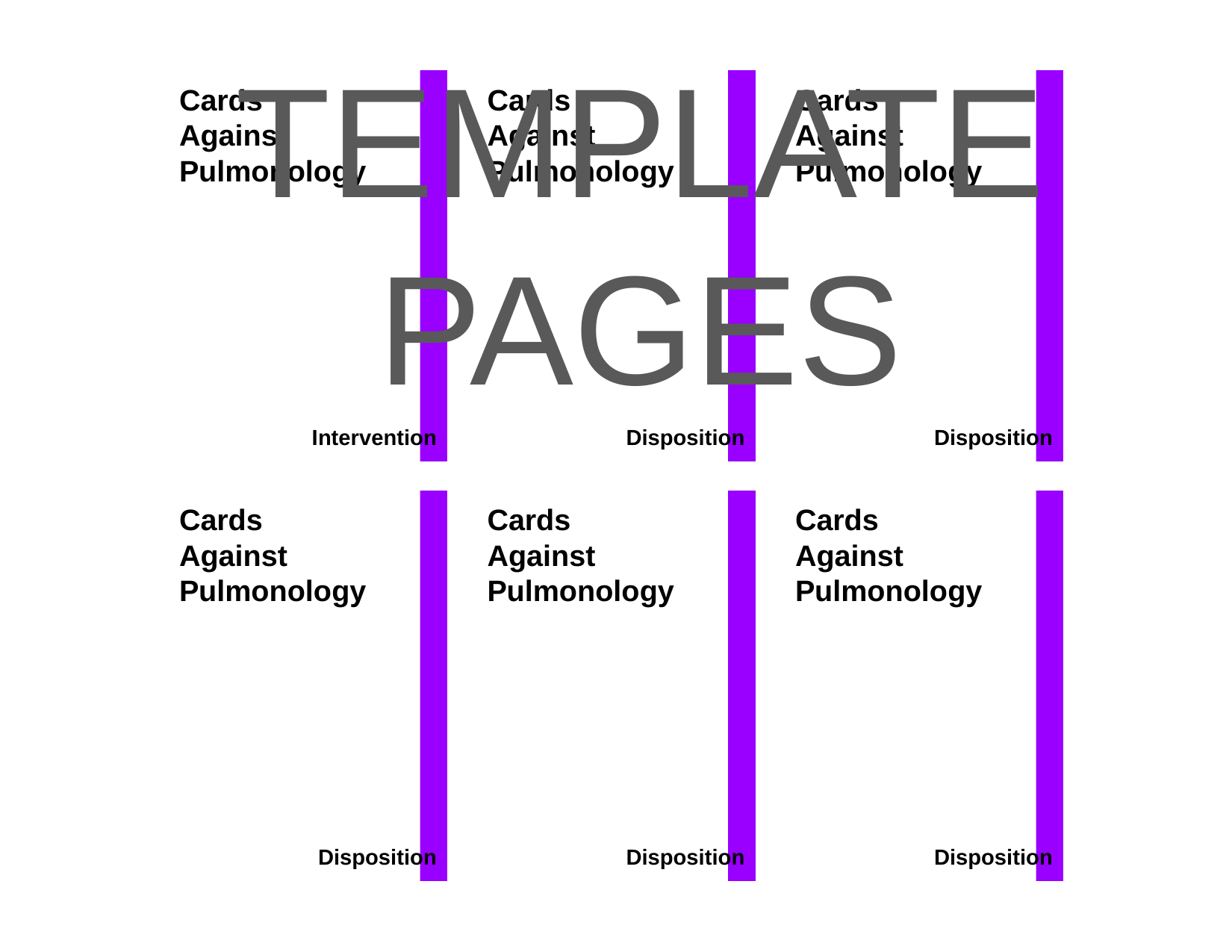

TEMPLATE PAGES
Cards
Against
Pulmonology
Intervention
Cards
Against
Pulmonology
Disposition
Cards
Against
Pulmonology
Disposition
Cards
Against
Pulmonology
Disposition
Cards
Against
Pulmonology
Disposition
Cards
Against
Pulmonology
Disposition

## Slide 8
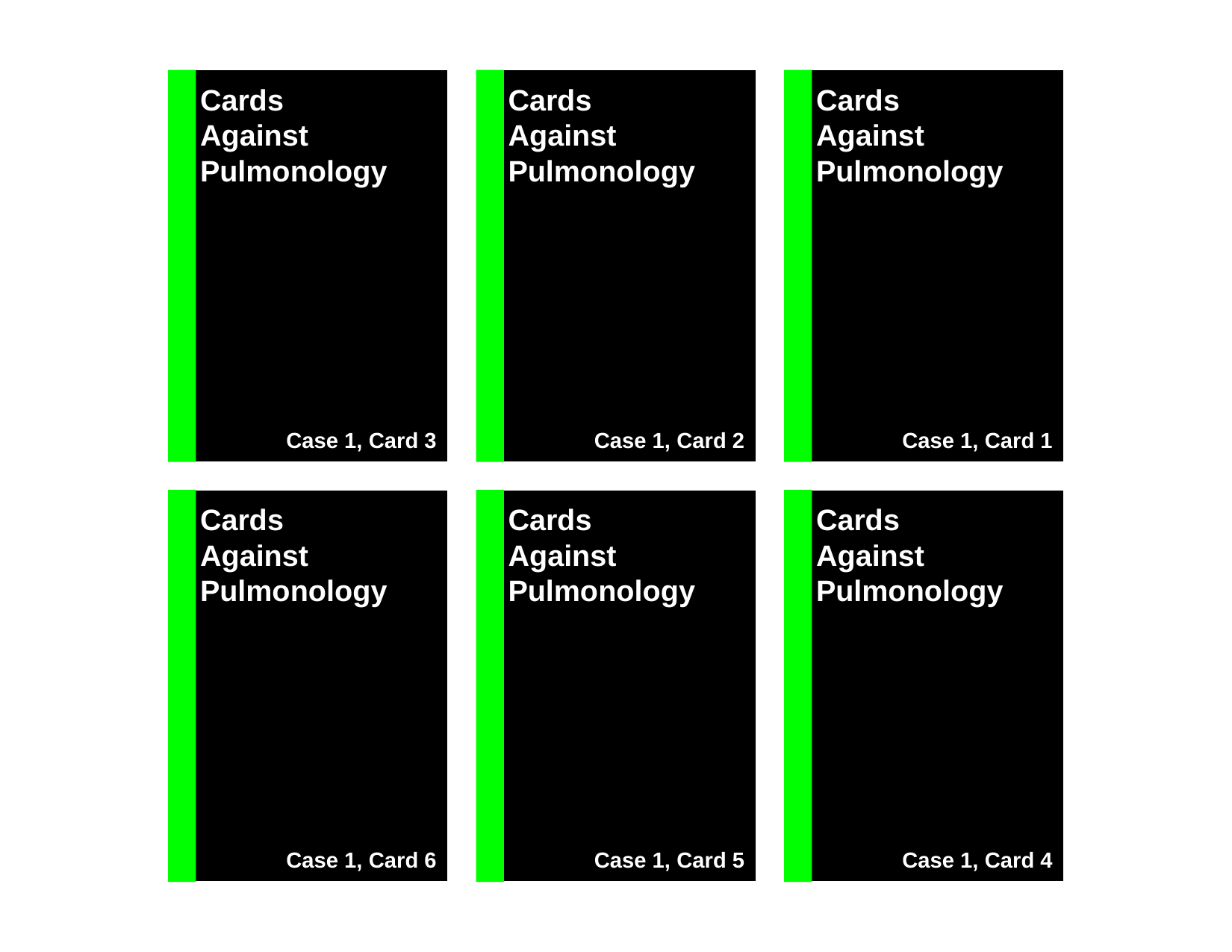

Cards
Against
Pulmonology
Case 1, Card 3
Cards
Against
Pulmonology
Case 1, Card 2
Cards
Against
Pulmonology
Case 1, Card 1
Cards
Against
Pulmonology
Case 1, Card 6
Cards
Against
Pulmonology
Case 1, Card 5
Cards
Against
Pulmonology
Case 1, Card 4

## Slide 9
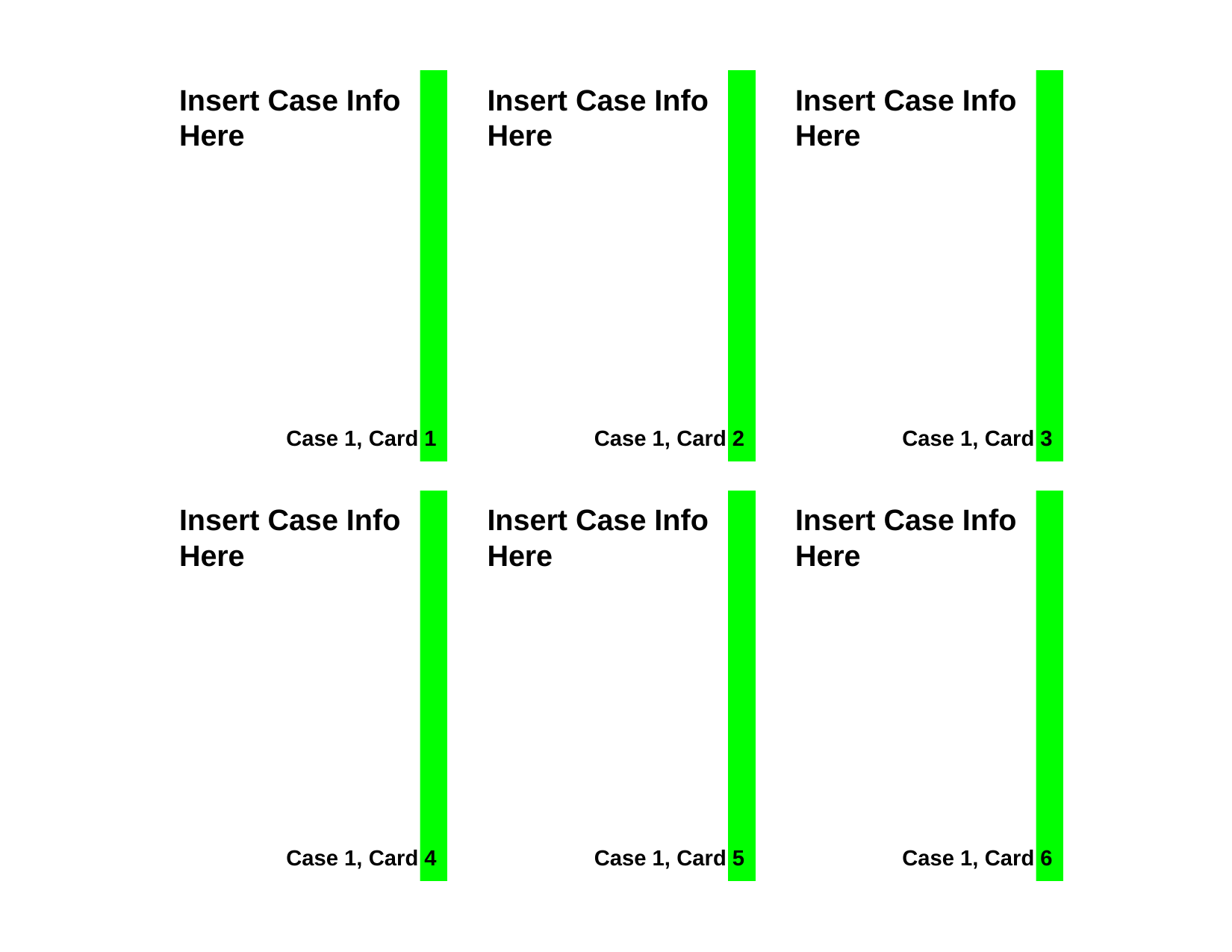

Insert Case Info Here
Case 1, Card 1
Insert Case Info Here
Case 1, Card 2
Insert Case Info Here
Case 1, Card 3
Insert Case Info Here
Case 1, Card 4
Insert Case Info Here
Case 1, Card 5
Insert Case Info Here
Case 1, Card 6

## Slide 10
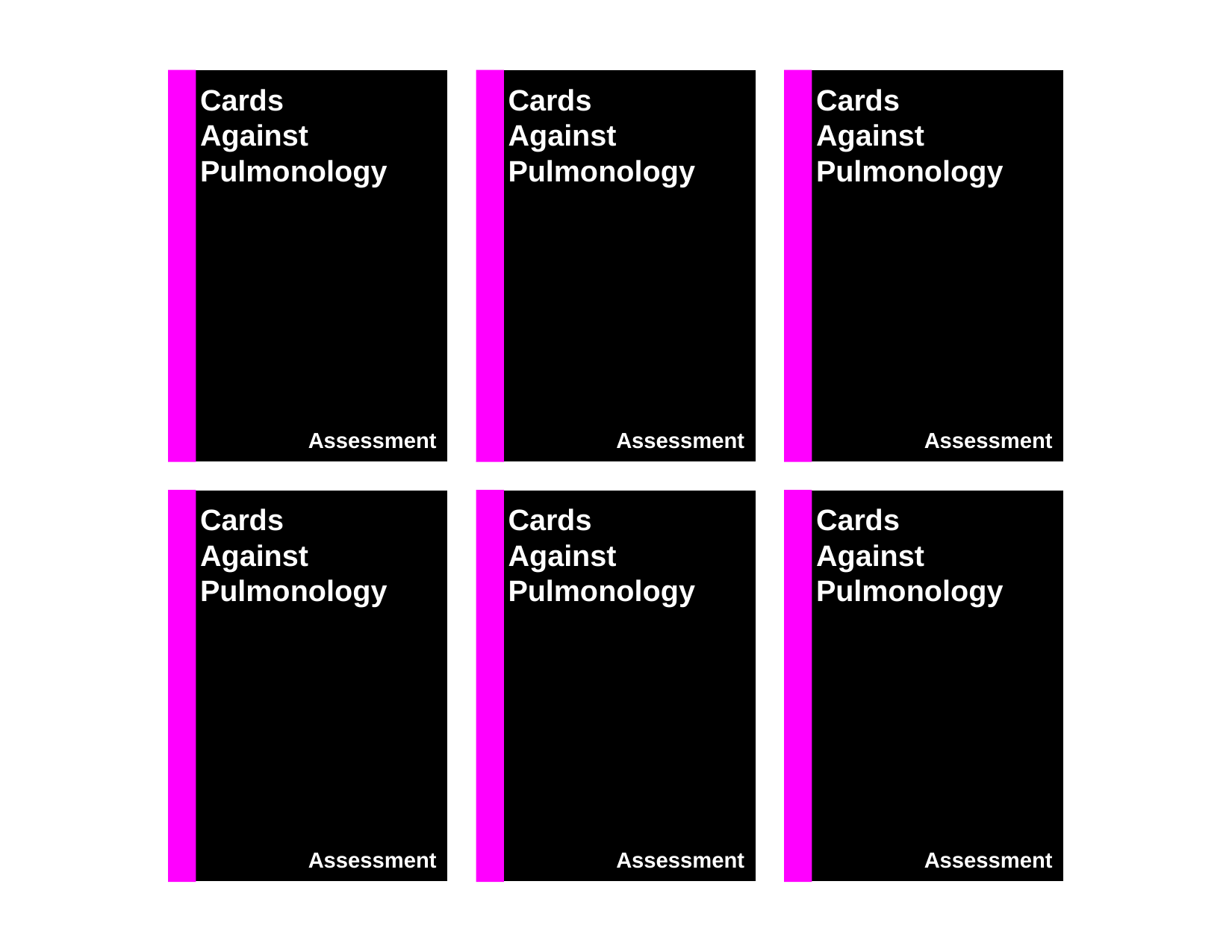

Cards
Against
Pulmonology
Assessment
Cards
Against
Pulmonology
Assessment
Cards
Against
Pulmonology
Assessment
Cards
Against
Pulmonology
Assessment
Cards
Against
Pulmonology
Assessment
Cards
Against
Pulmonology
Assessment

## Slide 11
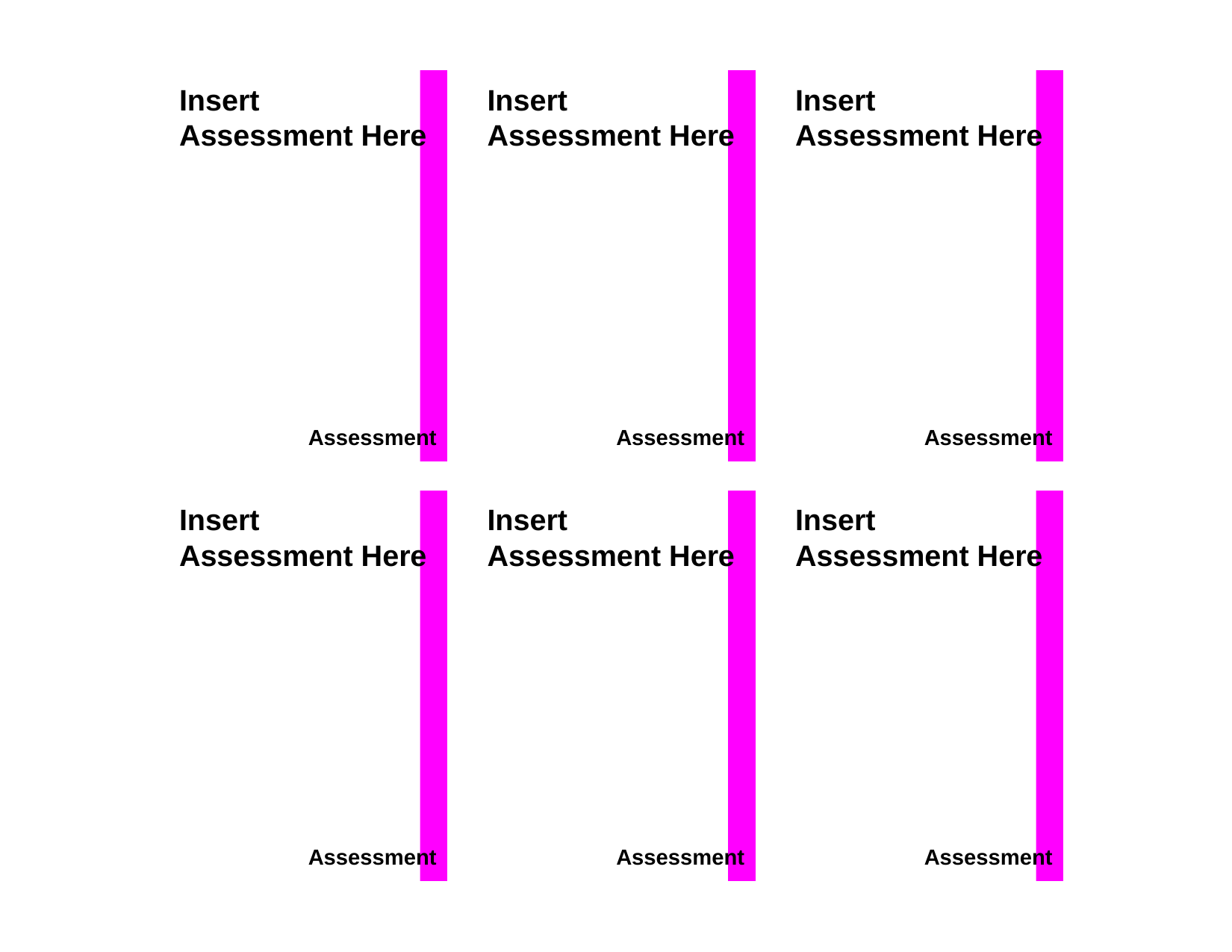

Insert Assessment Here
Assessment
Insert Assessment Here
Assessment
Insert Assessment Here
Assessment
Insert Assessment Here
Assessment
Insert Assessment Here
Assessment
Insert Assessment Here
Assessment

## Slide 12
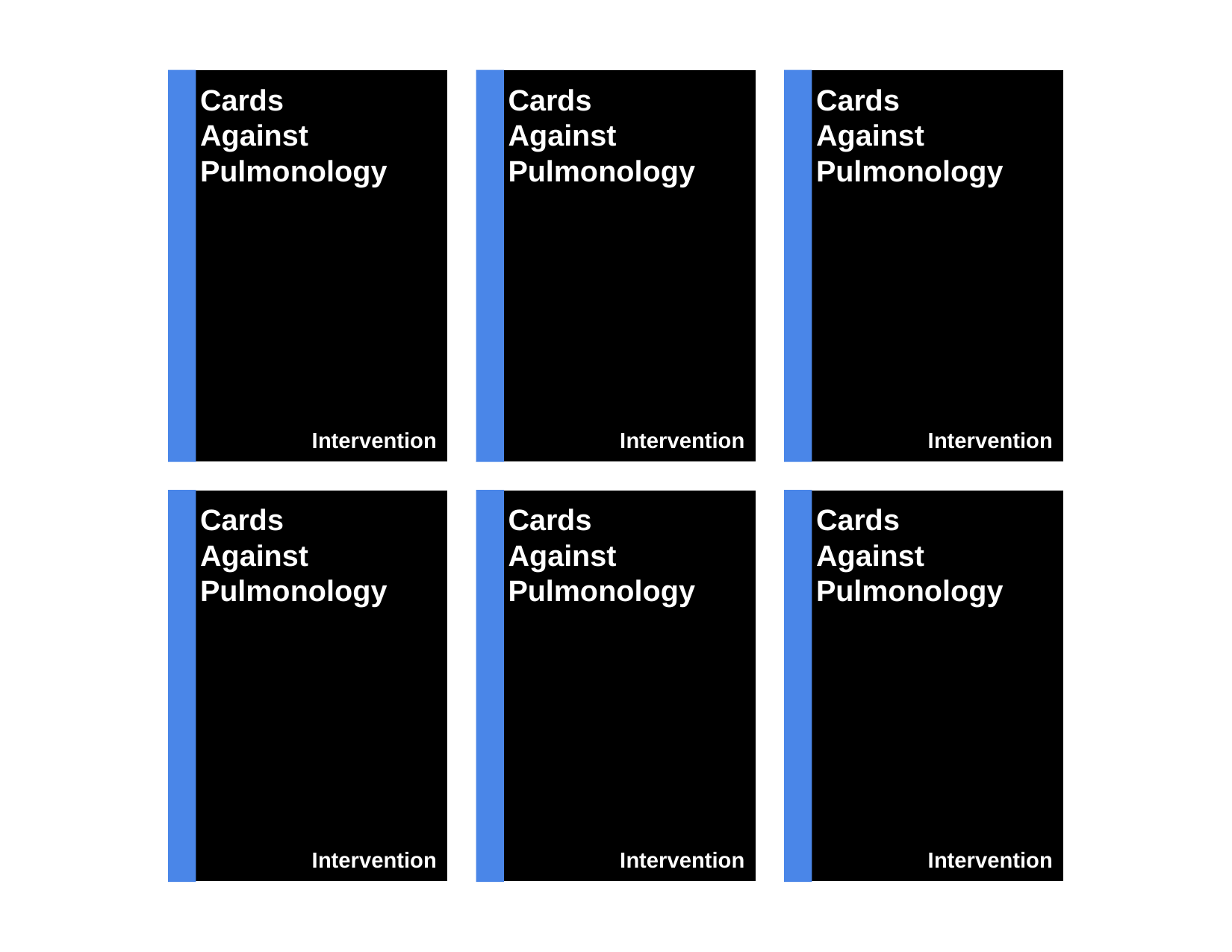

Cards
Against
Pulmonology
Intervention
Cards
Against
Pulmonology
Intervention
Cards
Against
Pulmonology
Intervention
Cards
Against
Pulmonology
Intervention
Cards
Against
Pulmonology
Intervention
Cards
Against
Pulmonology
Intervention

## Slide 13
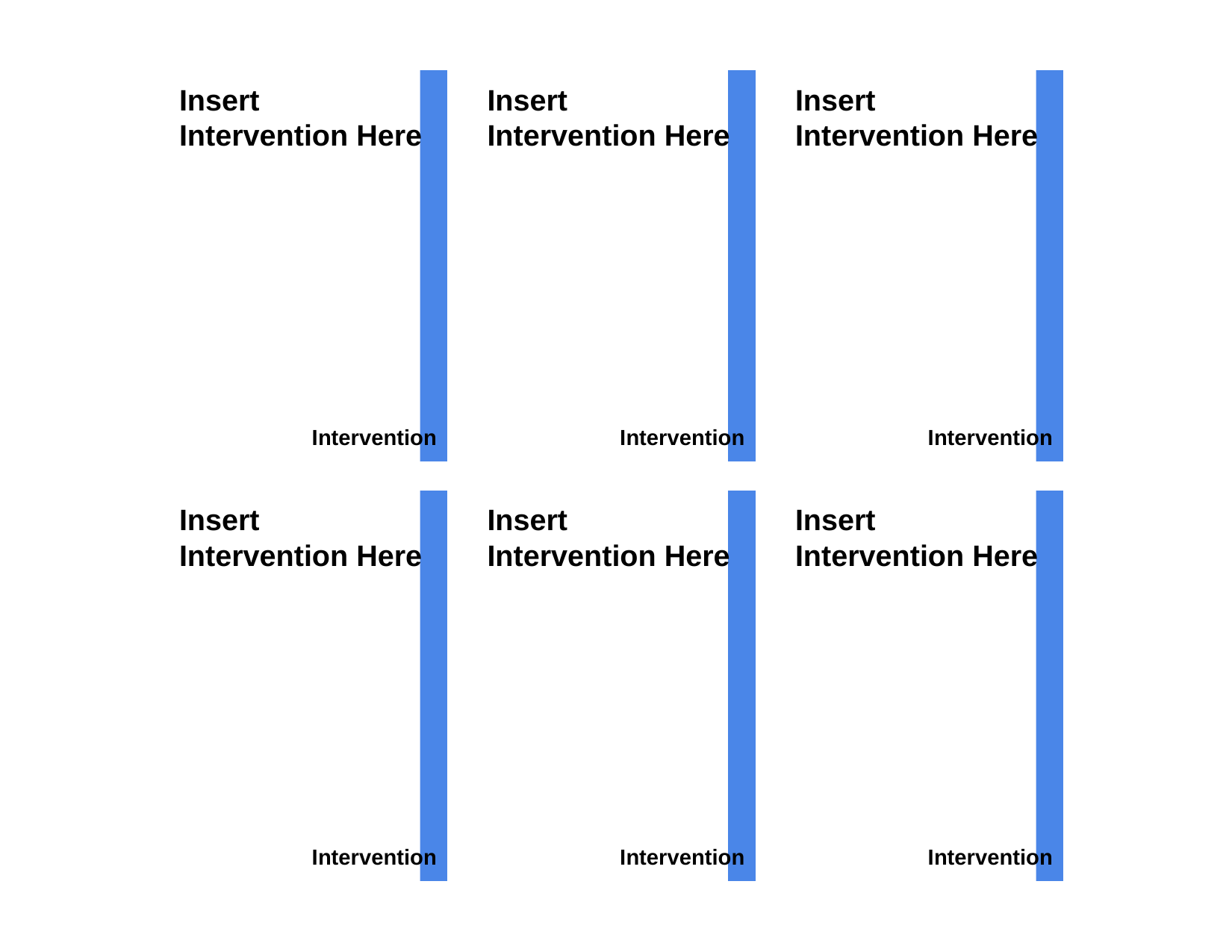

Insert Intervention Here
Intervention
Insert Intervention Here
Intervention
Insert Intervention Here
Intervention
Insert Intervention Here
Intervention
Insert Intervention Here
Intervention
Insert Intervention Here
Intervention

## Slide 14
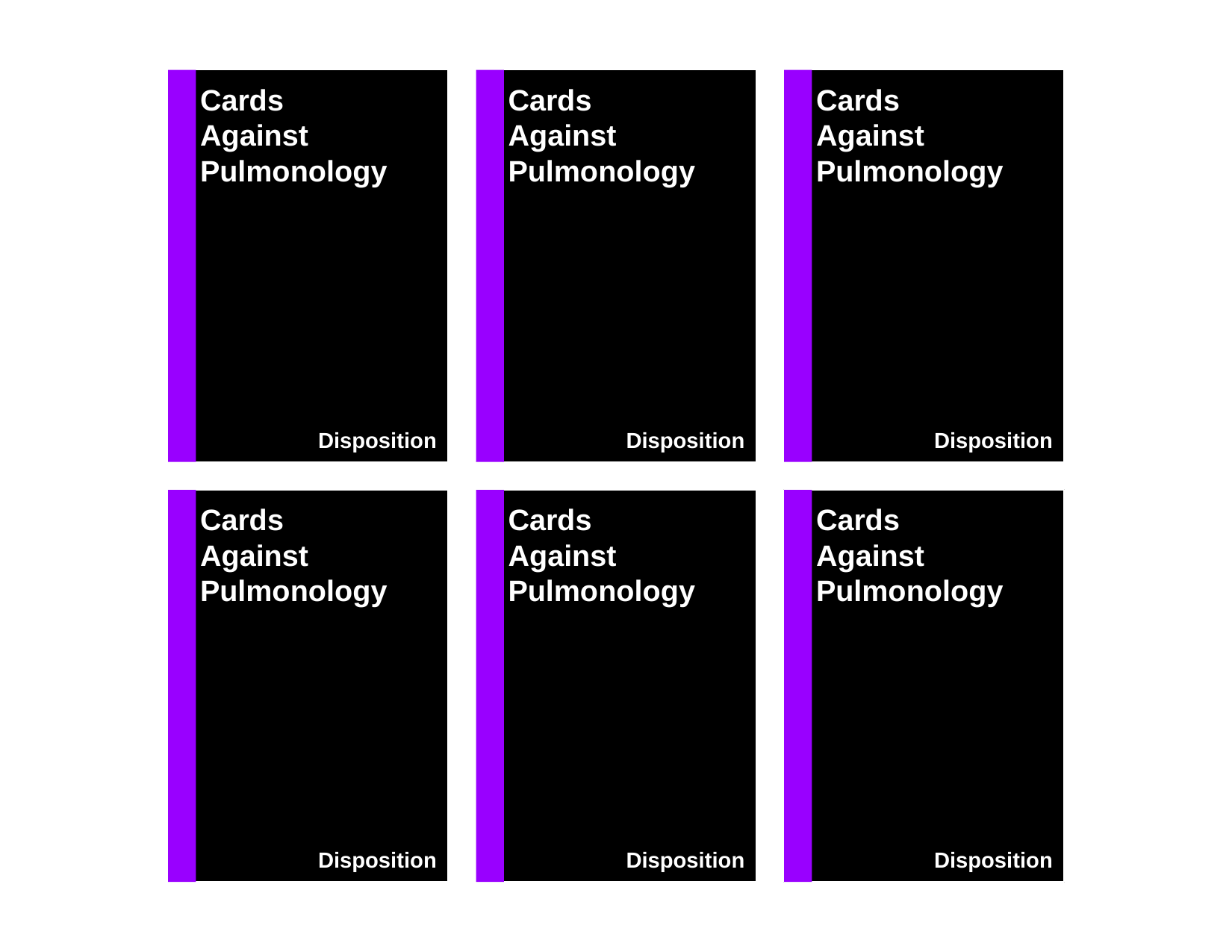

Cards
Against
Pulmonology
Disposition
Cards
Against
Pulmonology
Disposition
Cards
Against
Pulmonology
Disposition
Cards
Against
Pulmonology
Disposition
Cards
Against
Pulmonology
Disposition
Cards
Against
Pulmonology
Disposition

## Slide 15
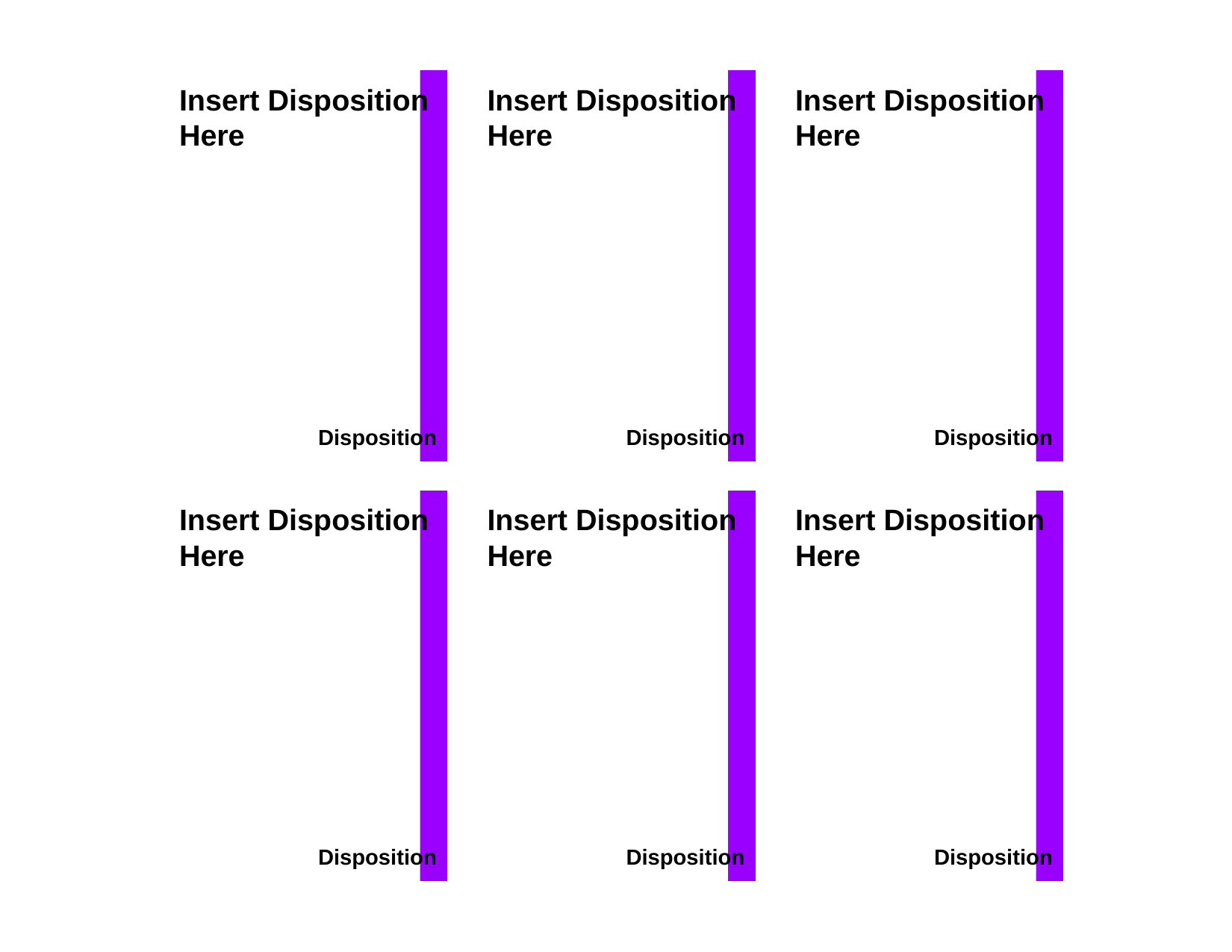

Insert Disposition Here
Disposition
Insert Disposition Here
Disposition
Insert Disposition Here
Disposition
Insert Disposition Here
Disposition
Insert Disposition Here
Disposition
Insert Disposition Here
Disposition

## Slide 16
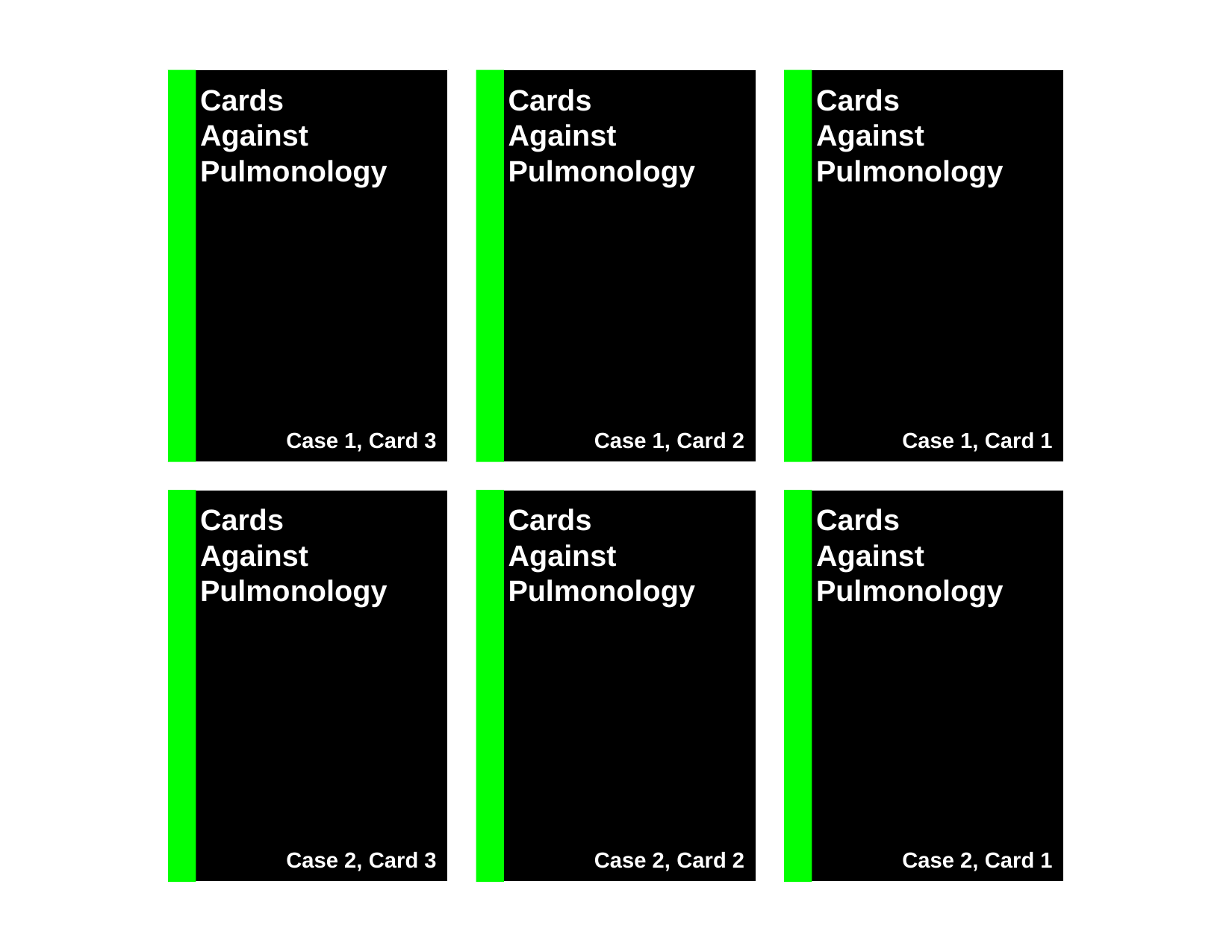

Cards
Against
Pulmonology
Case 1, Card 3
Cards
Against
Pulmonology
Case 1, Card 2
Cards
Against
Pulmonology
Case 1, Card 1
Cards
Against
Pulmonology
Case 2, Card 3
Cards
Against
Pulmonology
Case 2, Card 2
Cards
Against
Pulmonology
Case 2, Card 1

## Slide 17
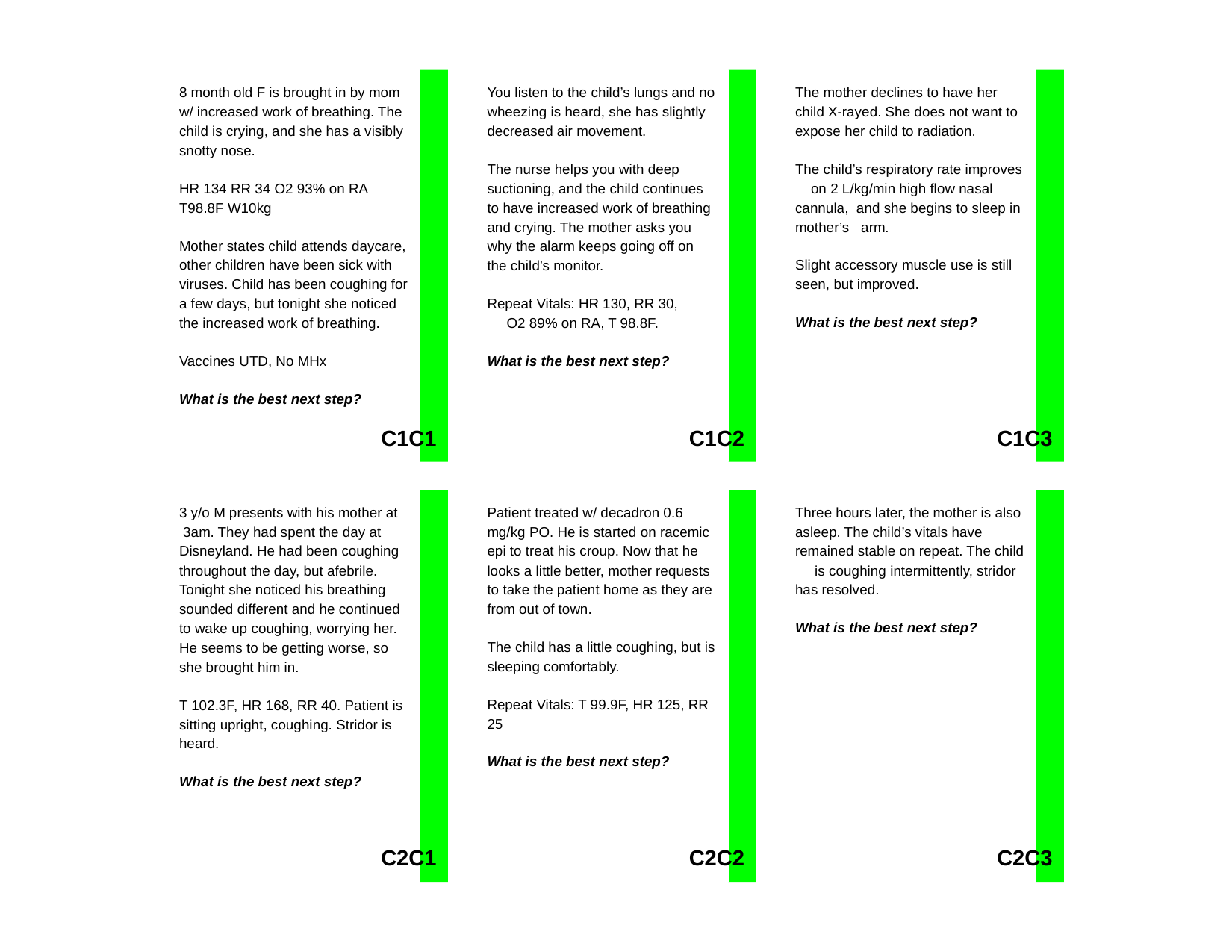

8 month old F is brought in by mom w/ increased work of breathing. The child is crying, and she has a visibly snotty nose.
HR 134 RR 34 O2 93% on RA T98.8F W10kg
Mother states child attends daycare, other children have been sick with viruses. Child has been coughing for a few days, but tonight she noticed the increased work of breathing.
Vaccines UTD, No MHx
What is the best next step?
C1C1
You listen to the child’s lungs and no wheezing is heard, she has slightly decreased air movement.
The nurse helps you with deep suctioning, and the child continues to have increased work of breathing and crying. The mother asks you why the alarm keeps going off on the child’s monitor.
Repeat Vitals: HR 130, RR 30, O2 89% on RA, T 98.8F.
What is the best next step?
C1C2
The mother declines to have her child X-rayed. She does not want to expose her child to radiation.
The child’s respiratory rate improves on 2 L/kg/min high flow nasal cannula, and she begins to sleep in mother’s arm.
Slight accessory muscle use is still seen, but improved.
What is the best next step?
C1C3
3 y/o M presents with his mother at 3am. They had spent the day at Disneyland. He had been coughing throughout the day, but afebrile. Tonight she noticed his breathing sounded different and he continued to wake up coughing, worrying her. He seems to be getting worse, so she brought him in.
T 102.3F, HR 168, RR 40. Patient is sitting upright, coughing. Stridor is heard.
What is the best next step?
C2C1
Patient treated w/ decadron 0.6 mg/kg PO. He is started on racemic epi to treat his croup. Now that he looks a little better, mother requests to take the patient home as they are from out of town.
The child has a little coughing, but is sleeping comfortably.
Repeat Vitals: T 99.9F, HR 125, RR 25
What is the best next step?
C2C2
Three hours later, the mother is also asleep. The child’s vitals have remained stable on repeat. The child is coughing intermittently, stridor has resolved.
What is the best next step?
C2C3

## Slide 18
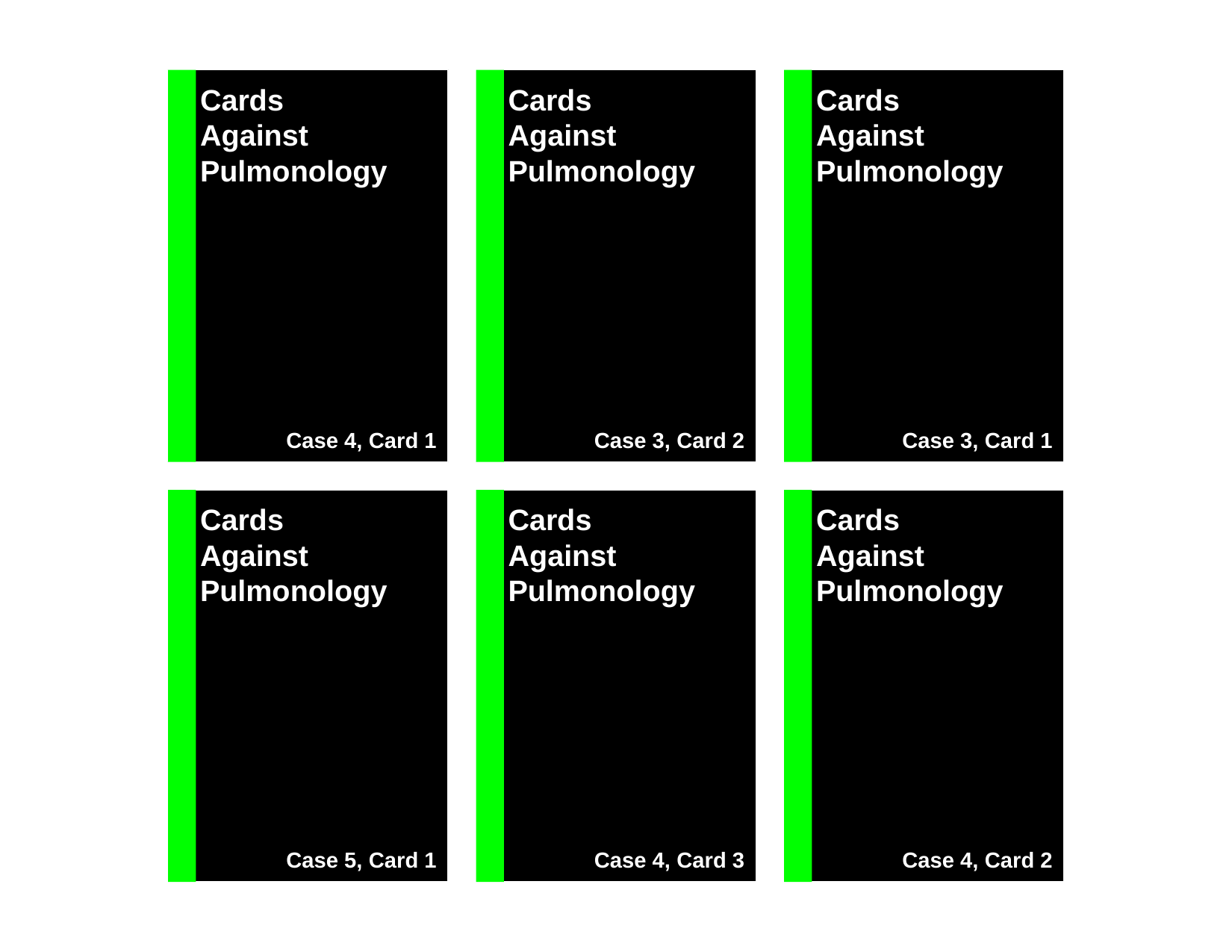

Cards
Against
Pulmonology
Case 4, Card 1
Cards
Against
Pulmonology
Case 3, Card 2
Cards
Against
Pulmonology
Case 3, Card 1
Cards
Against
Pulmonology
Case 5, Card 1
Cards
Against
Pulmonology
Case 4, Card 3
Cards
Against
Pulmonology
Case 4, Card 2

## Slide 19
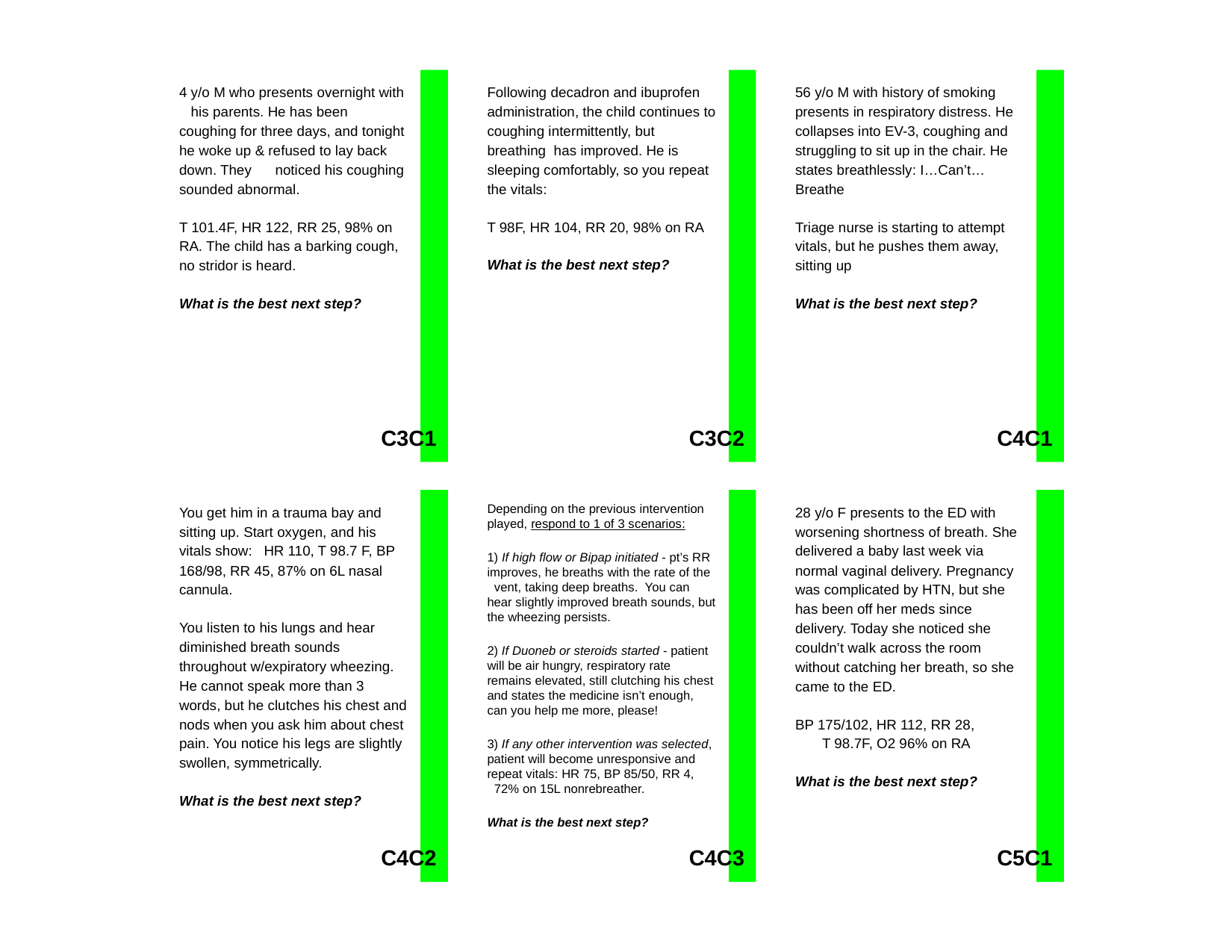

4 y/o M who presents overnight with his parents. He has been coughing for three days, and tonight he woke up & refused to lay back down. They noticed his coughing sounded abnormal.
T 101.4F, HR 122, RR 25, 98% on RA. The child has a barking cough, no stridor is heard.
What is the best next step?
C3C1
Following decadron and ibuprofen administration, the child continues to coughing intermittently, but breathing has improved. He is sleeping comfortably, so you repeat the vitals:
T 98F, HR 104, RR 20, 98% on RA
What is the best next step?
C3C2
56 y/o M with history of smoking presents in respiratory distress. He collapses into EV-3, coughing and struggling to sit up in the chair. He states breathlessly: I…Can’t…Breathe
Triage nurse is starting to attempt vitals, but he pushes them away, sitting up
What is the best next step?
C4C1
You get him in a trauma bay and sitting up. Start oxygen, and his vitals show: HR 110, T 98.7 F, BP 168/98, RR 45, 87% on 6L nasal cannula.
You listen to his lungs and hear diminished breath sounds throughout w/expiratory wheezing. He cannot speak more than 3 words, but he clutches his chest and nods when you ask him about chest pain. You notice his legs are slightly swollen, symmetrically.
What is the best next step?
C4C2
Depending on the previous intervention played, respond to 1 of 3 scenarios:
1) If high flow or Bipap initiated - pt’s RR improves, he breaths with the rate of the vent, taking deep breaths. You can hear slightly improved breath sounds, but the wheezing persists.
2) If Duoneb or steroids started - patient will be air hungry, respiratory rate remains elevated, still clutching his chest and states the medicine isn’t enough, can you help me more, please!
3) If any other intervention was selected, patient will become unresponsive and repeat vitals: HR 75, BP 85/50, RR 4, 72% on 15L nonrebreather.
What is the best next step?
C4C3
28 y/o F presents to the ED with worsening shortness of breath. She delivered a baby last week via normal vaginal delivery. Pregnancy was complicated by HTN, but she has been off her meds since delivery. Today she noticed she couldn’t walk across the room without catching her breath, so she came to the ED.
BP 175/102, HR 112, RR 28, T 98.7F, O2 96% on RA
What is the best next step?
C5C1

## Slide 20
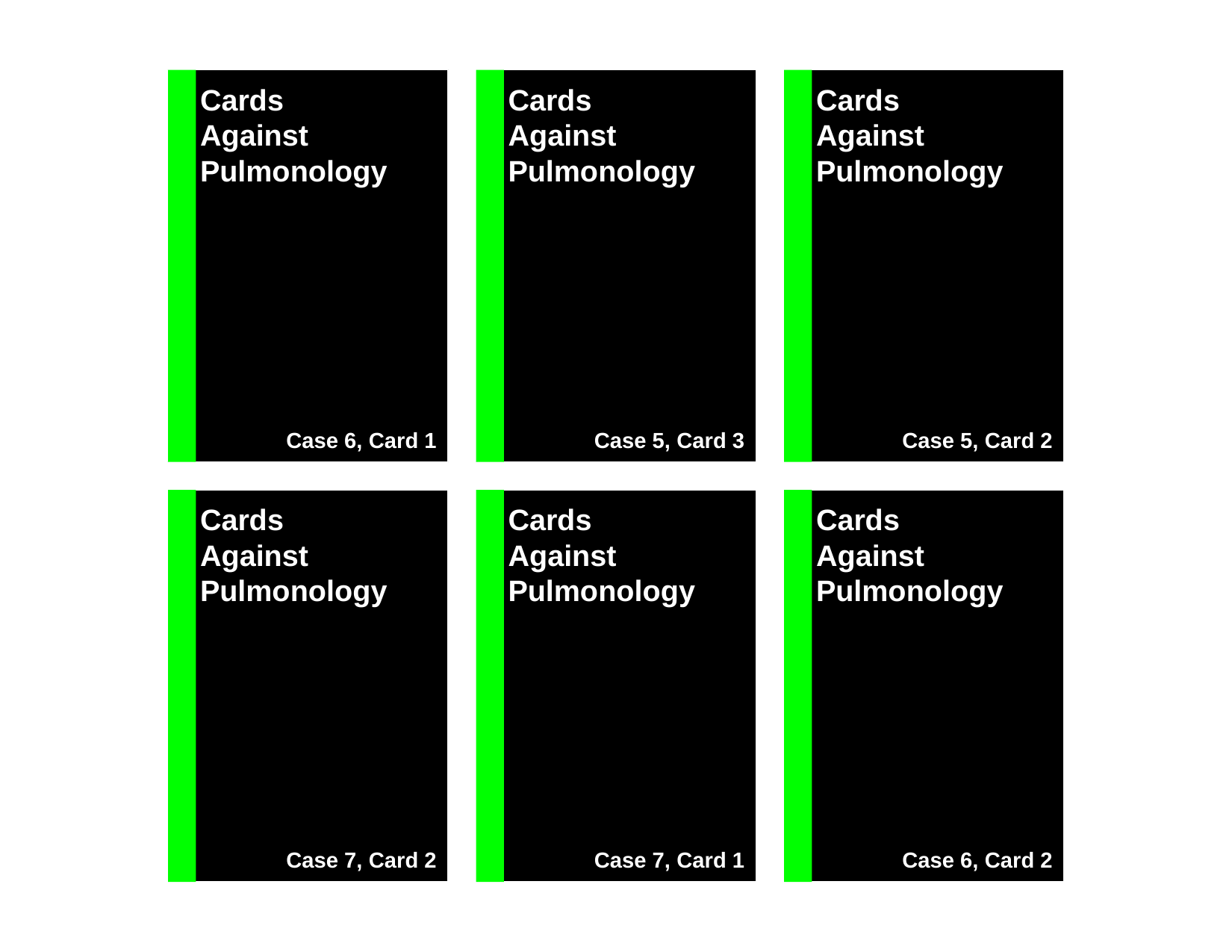

Cards
Against
Pulmonology
Case 6, Card 1
Cards
Against
Pulmonology
Case 5, Card 3
Cards
Against
Pulmonology
Case 5, Card 2
Cards
Against
Pulmonology
Case 7, Card 2
Cards
Against
Pulmonology
Case 7, Card 1
Cards
Against
Pulmonology
Case 6, Card 2

## Slide 21
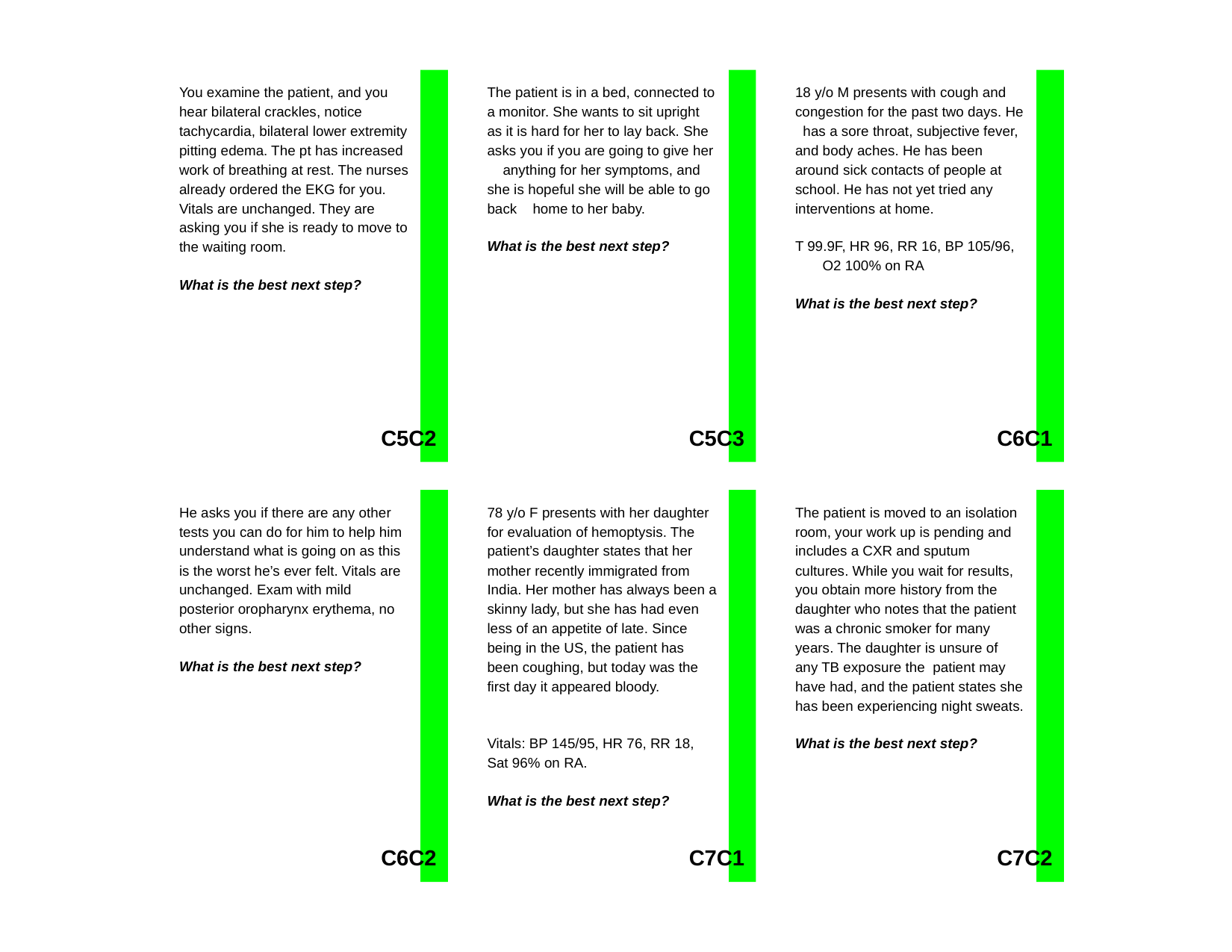

You examine the patient, and you hear bilateral crackles, notice tachycardia, bilateral lower extremity pitting edema. The pt has increased work of breathing at rest. The nurses already ordered the EKG for you. Vitals are unchanged. They are asking you if she is ready to move to the waiting room.
What is the best next step?
C5C2
The patient is in a bed, connected to a monitor. She wants to sit upright as it is hard for her to lay back. She asks you if you are going to give her anything for her symptoms, and she is hopeful she will be able to go back home to her baby.
What is the best next step?
C5C3
18 y/o M presents with cough and congestion for the past two days. He has a sore throat, subjective fever, and body aches. He has been around sick contacts of people at school. He has not yet tried any interventions at home.
T 99.9F, HR 96, RR 16, BP 105/96, O2 100% on RA
What is the best next step?
C6C1
He asks you if there are any other tests you can do for him to help him understand what is going on as this is the worst he’s ever felt. Vitals are unchanged. Exam with mild posterior oropharynx erythema, no other signs.
What is the best next step?
C6C2
78 y/o F presents with her daughter for evaluation of hemoptysis. The patient’s daughter states that her mother recently immigrated from India. Her mother has always been a skinny lady, but she has had even less of an appetite of late. Since being in the US, the patient has been coughing, but today was the first day it appeared bloody.
Vitals: BP 145/95, HR 76, RR 18, Sat 96% on RA.
What is the best next step?
C7C1
The patient is moved to an isolation room, your work up is pending and includes a CXR and sputum cultures. While you wait for results, you obtain more history from the daughter who notes that the patient was a chronic smoker for many years. The daughter is unsure of any TB exposure the patient may have had, and the patient states she has been experiencing night sweats.
What is the best next step?
C7C2

## Slide 22
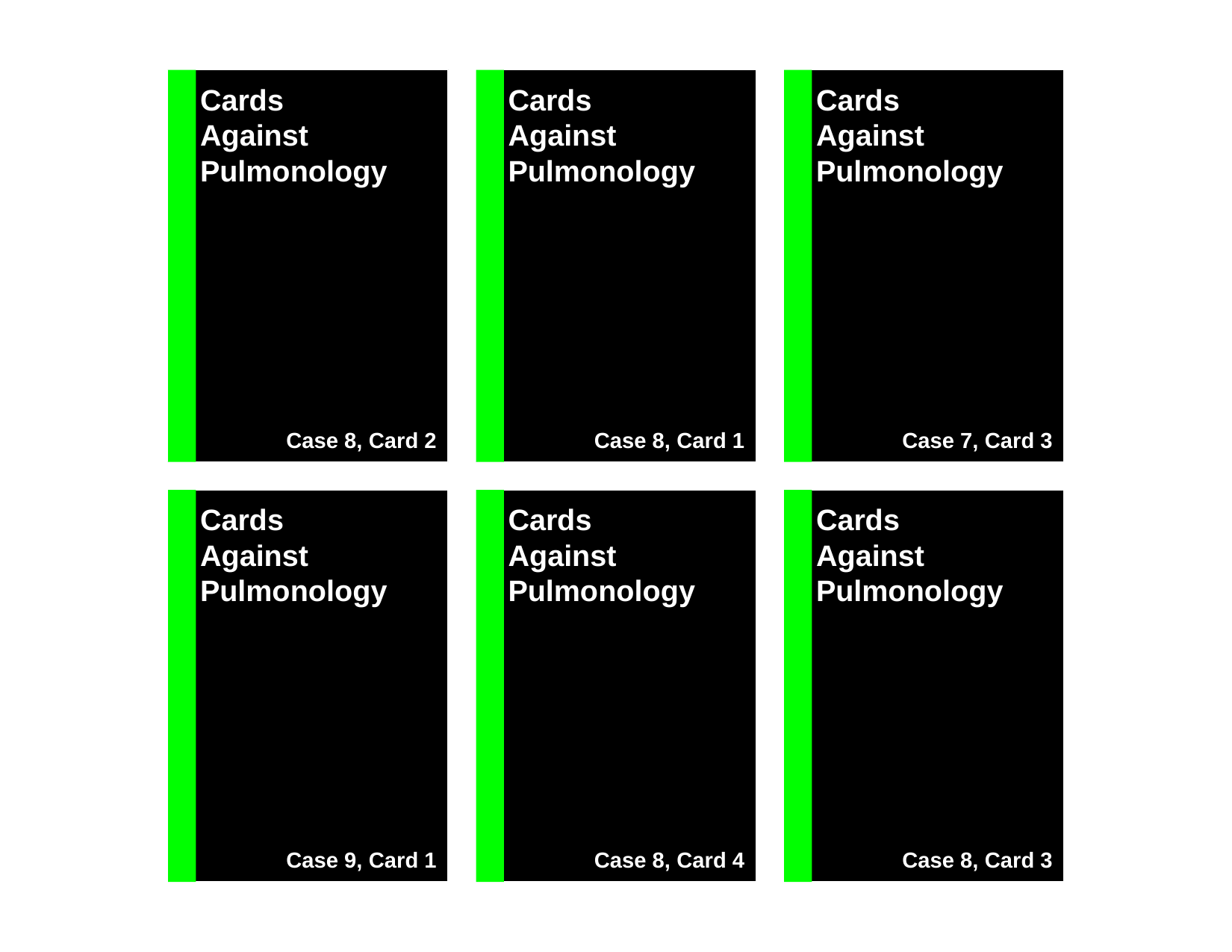

Cards
Against
Pulmonology
Case 8, Card 2
Cards
Against
Pulmonology
Case 8, Card 1
Cards
Against
Pulmonology
Case 7, Card 3
Cards
Against
Pulmonology
Case 9, Card 1
Cards
Against
Pulmonology
Case 8, Card 4
Cards
Against
Pulmonology
Case 8, Card 3

## Slide 23
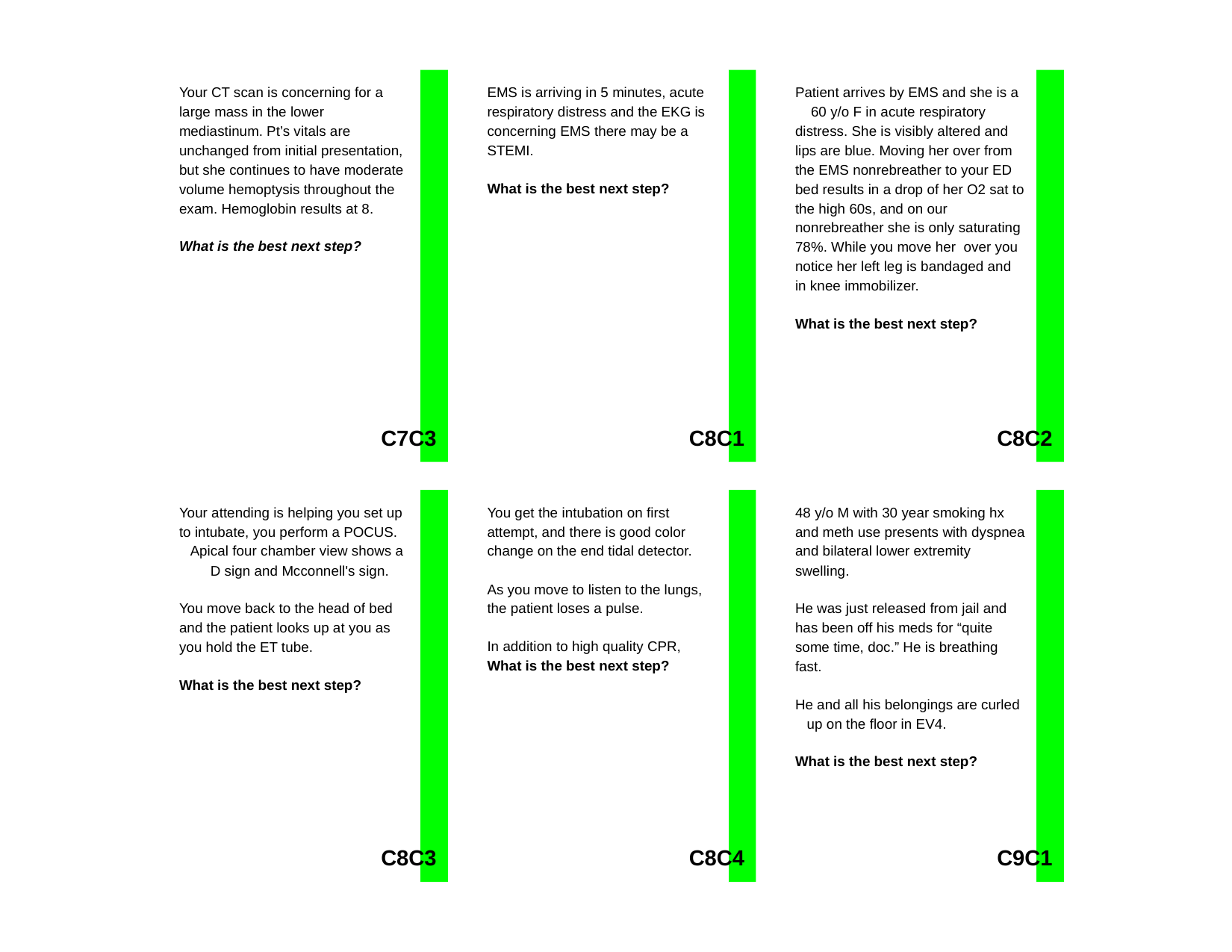

Your CT scan is concerning for a large mass in the lower mediastinum. Pt’s vitals are unchanged from initial presentation, but she continues to have moderate volume hemoptysis throughout the exam. Hemoglobin results at 8.
What is the best next step?
C7C3
EMS is arriving in 5 minutes, acute respiratory distress and the EKG is concerning EMS there may be a STEMI.
What is the best next step?
C8C1
Patient arrives by EMS and she is a 60 y/o F in acute respiratory distress. She is visibly altered and lips are blue. Moving her over from the EMS nonrebreather to your ED bed results in a drop of her O2 sat to the high 60s, and on our nonrebreather she is only saturating 78%. While you move her over you notice her left leg is bandaged and in knee immobilizer.
What is the best next step?
C8C2
Your attending is helping you set up to intubate, you perform a POCUS. Apical four chamber view shows a D sign and Mcconnell's sign.
You move back to the head of bed and the patient looks up at you as you hold the ET tube.
What is the best next step?
C8C3
You get the intubation on first attempt, and there is good color change on the end tidal detector.
As you move to listen to the lungs, the patient loses a pulse.
In addition to high quality CPR, What is the best next step?
C8C4
48 y/o M with 30 year smoking hx and meth use presents with dyspnea and bilateral lower extremity swelling.
He was just released from jail and has been off his meds for “quite some time, doc.” He is breathing fast.
He and all his belongings are curled up on the floor in EV4.
What is the best next step?
C9C1

## Slide 24
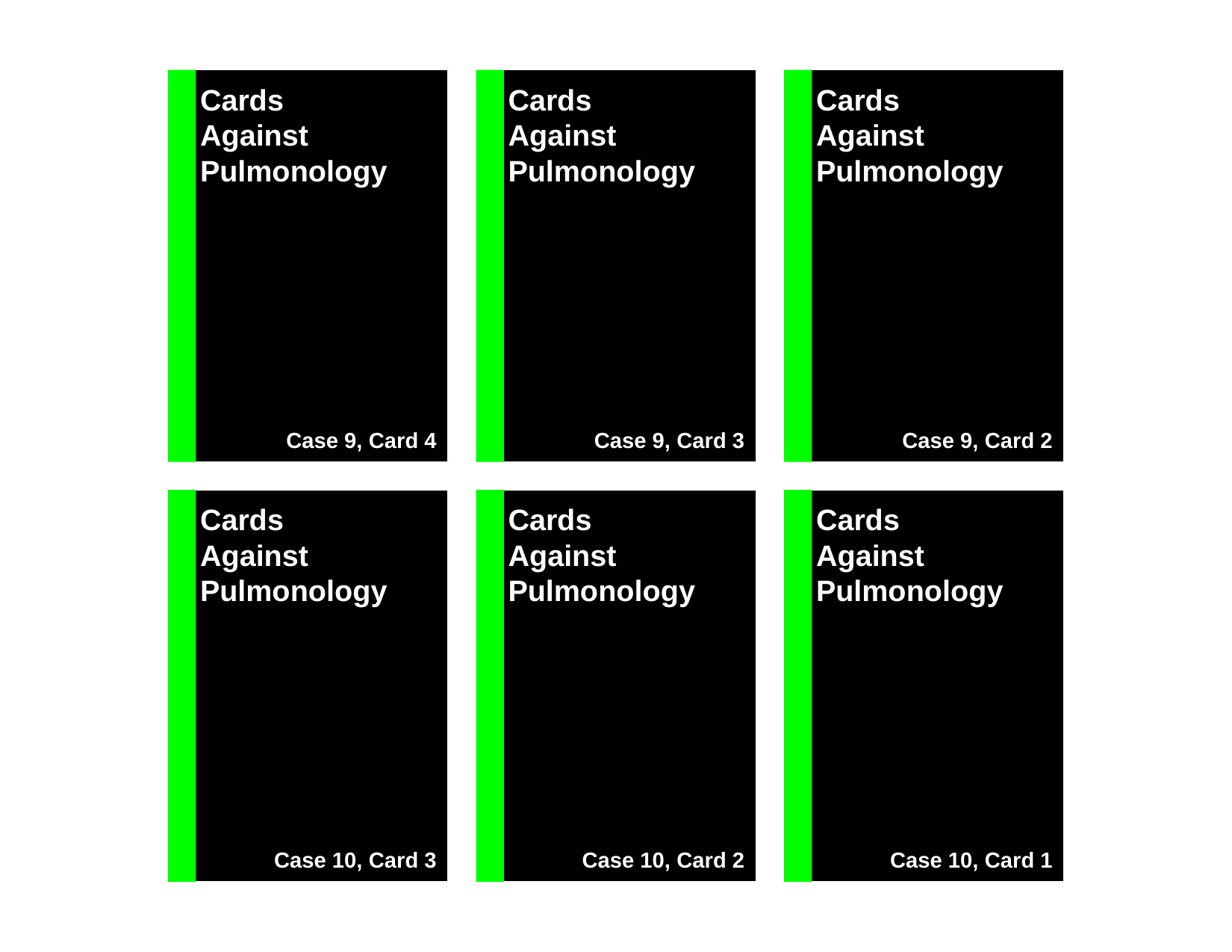

Cards
Against
Pulmonology
Case 9, Card 4
Cards
Against
Pulmonology
Case 9, Card 3
Cards
Against
Pulmonology
Case 9, Card 2
Cards
Against
Pulmonology
Case 10, Card 3
Cards
Against
Pulmonology
Case 10, Card 2
Cards
Against
Pulmonology
Case 10, Card 1

## Slide 25
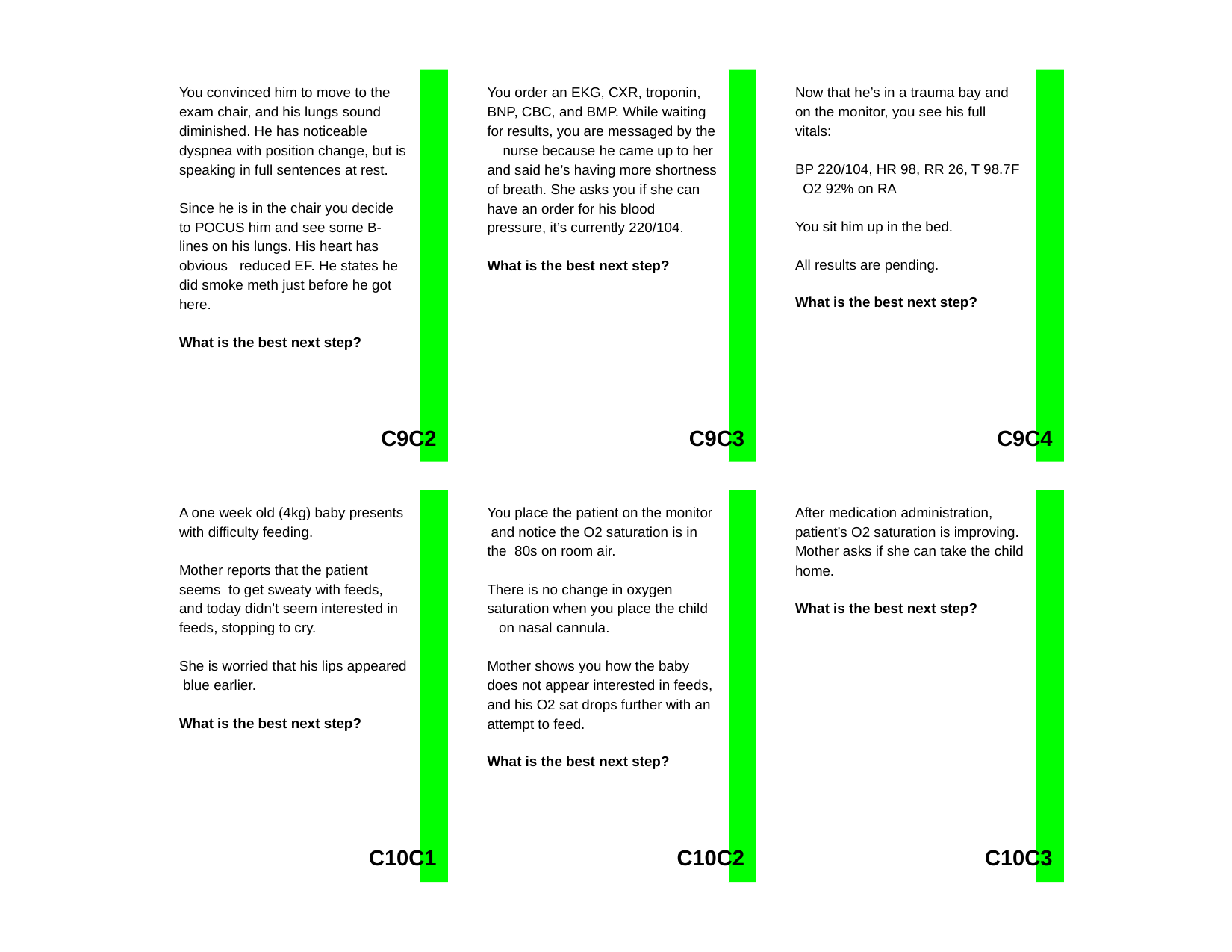

You convinced him to move to the exam chair, and his lungs sound diminished. He has noticeable dyspnea with position change, but is speaking in full sentences at rest.
Since he is in the chair you decide to POCUS him and see some B-lines on his lungs. His heart has obvious reduced EF. He states he did smoke meth just before he got here.
What is the best next step?
C9C2
You order an EKG, CXR, troponin, BNP, CBC, and BMP. While waiting for results, you are messaged by the nurse because he came up to her and said he’s having more shortness of breath. She asks you if she can have an order for his blood pressure, it’s currently 220/104.
What is the best next step?
C9C3
Now that he’s in a trauma bay and on the monitor, you see his full vitals:
BP 220/104, HR 98, RR 26, T 98.7F O2 92% on RA
You sit him up in the bed.
All results are pending.
What is the best next step?
C9C4
A one week old (4kg) baby presents with difficulty feeding.
Mother reports that the patient seems to get sweaty with feeds, and today didn’t seem interested in feeds, stopping to cry.
She is worried that his lips appeared blue earlier.
What is the best next step?
C10C1
You place the patient on the monitor and notice the O2 saturation is in the 80s on room air.
There is no change in oxygen saturation when you place the child on nasal cannula.
Mother shows you how the baby does not appear interested in feeds, and his O2 sat drops further with an attempt to feed.
What is the best next step?
C10C2
After medication administration, patient’s O2 saturation is improving. Mother asks if she can take the child home.
What is the best next step?
C10C3

## Slide 26
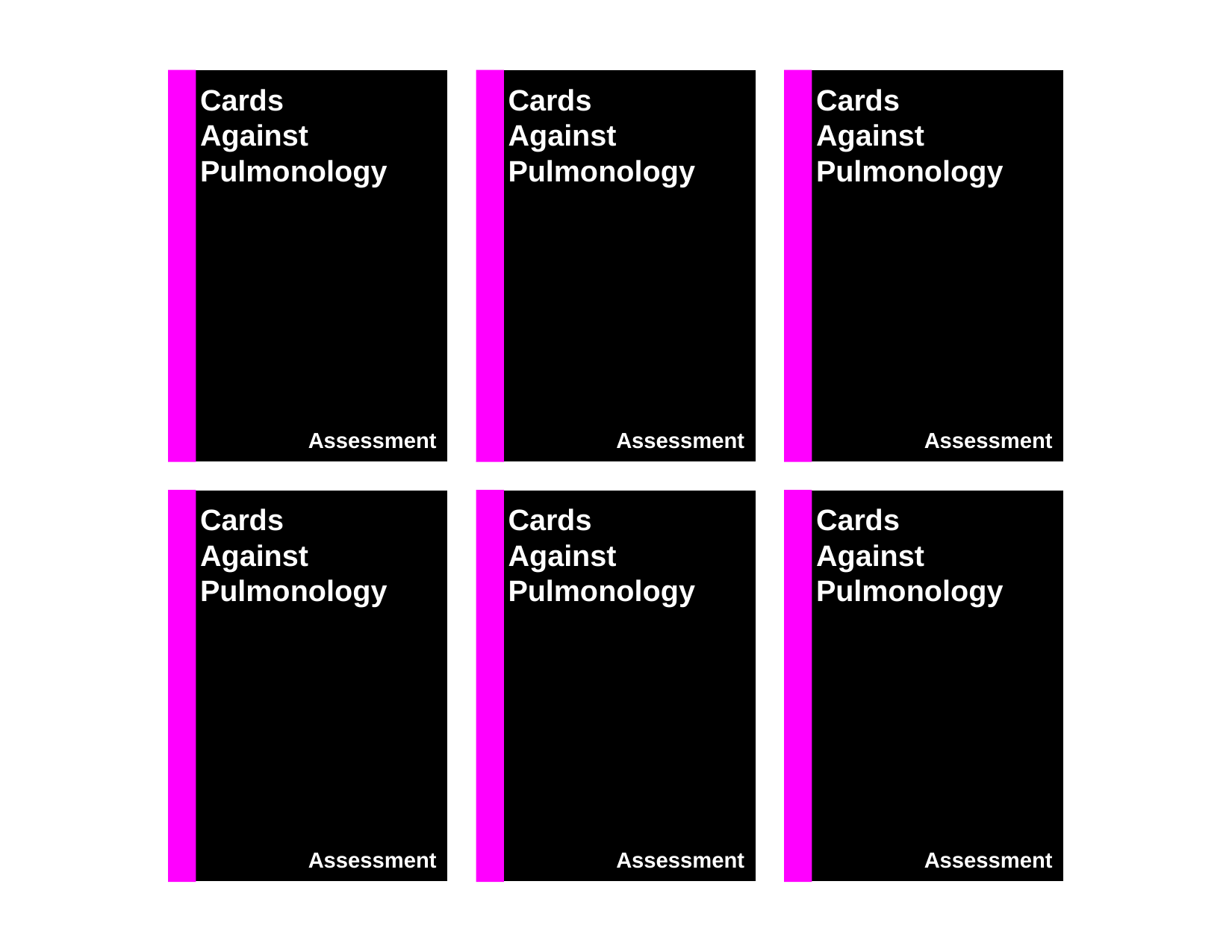

Cards
Against
Pulmonology
Assessment
Cards
Against
Pulmonology
Assessment
Cards
Against
Pulmonology
Assessment
Cards
Against
Pulmonology
Assessment
Cards
Against
Pulmonology
Assessment
Cards
Against
Pulmonology
Assessment

## Slide 27
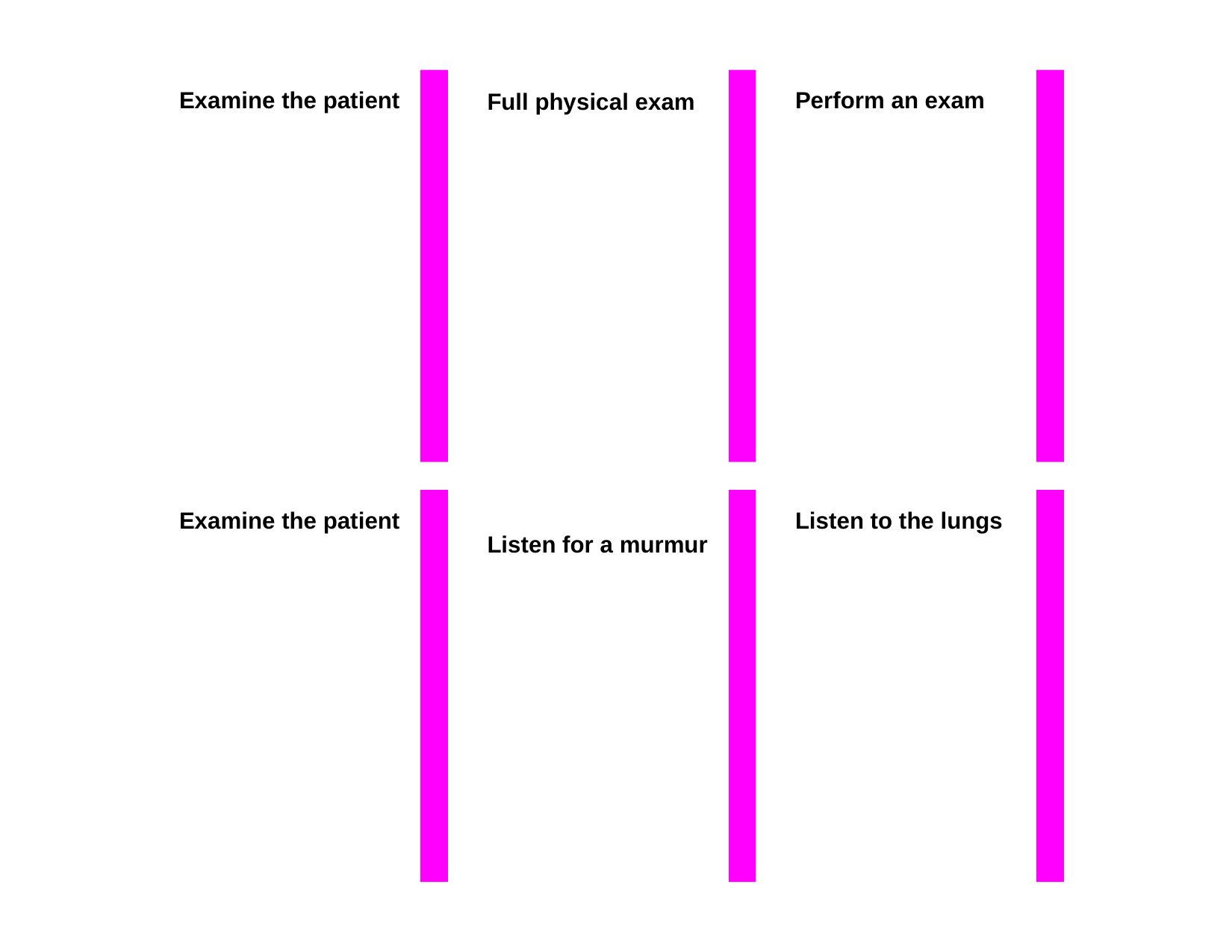

Examine the patient
Full physical exam
Perform an exam
Examine the patient
Listen for a murmur
Listen to the lungs

## Slide 28
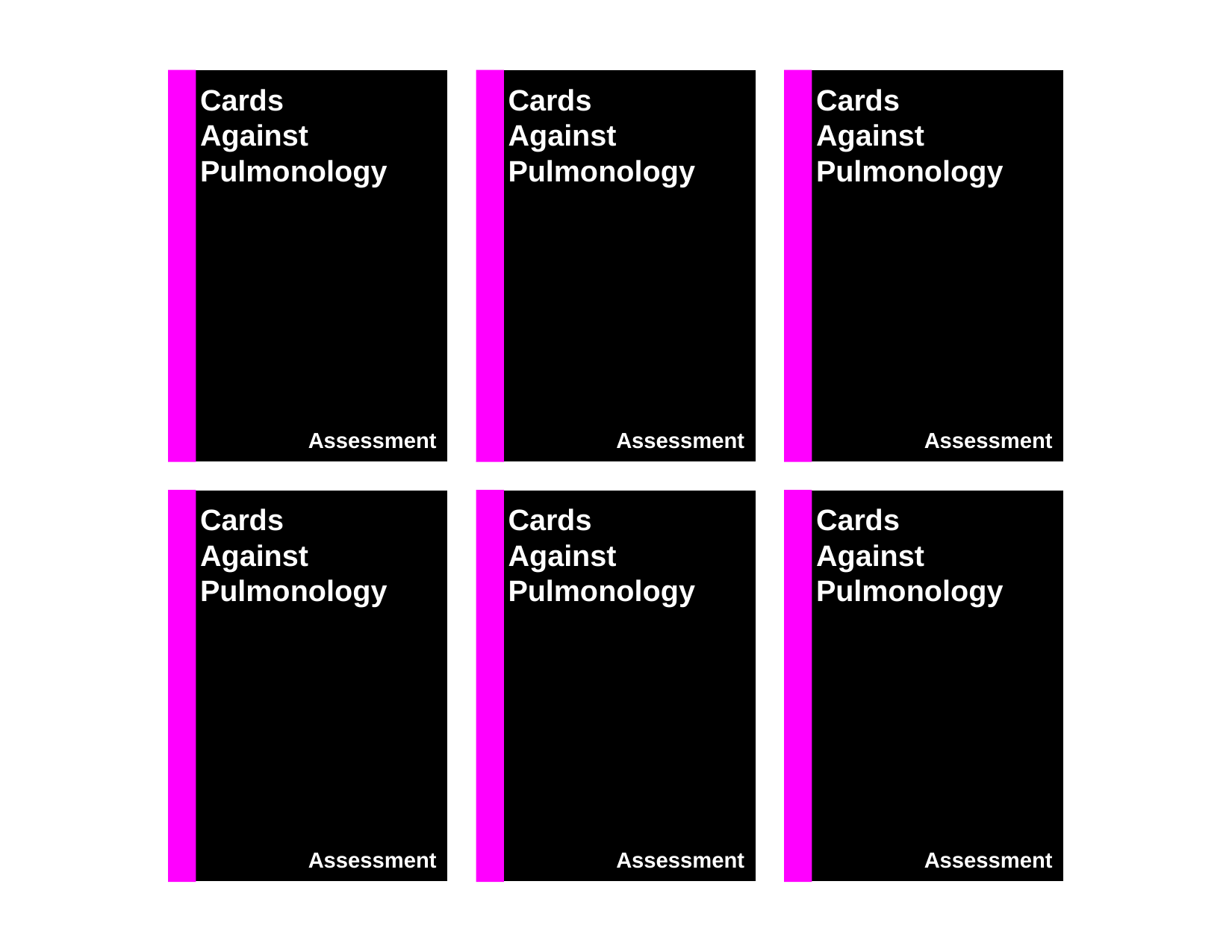

Cards
Against
Pulmonology
Assessment
Cards
Against
Pulmonology
Assessment
Cards
Against
Pulmonology
Assessment
Cards
Against
Pulmonology
Assessment
Cards
Against
Pulmonology
Assessment
Cards
Against
Pulmonology
Assessment

## Slide 29
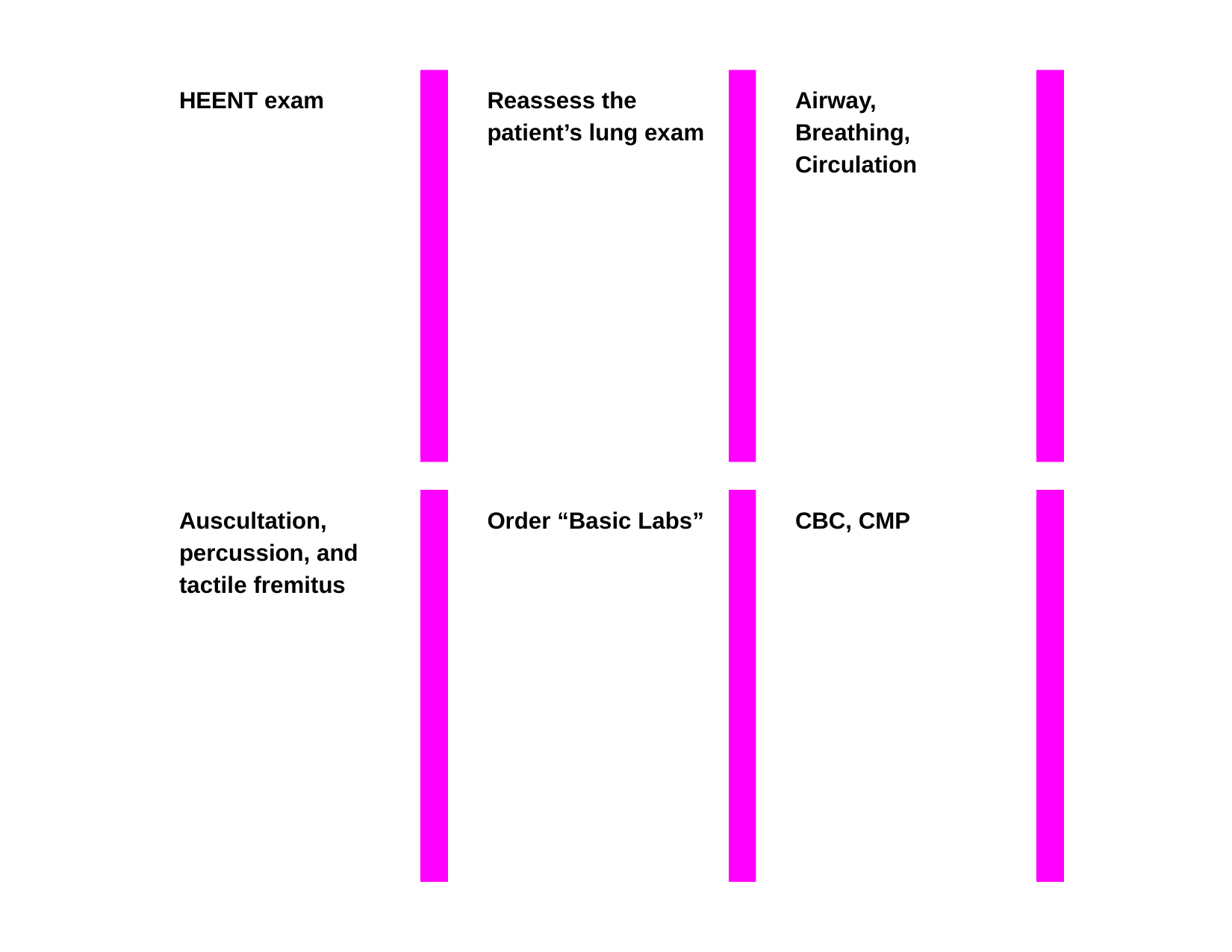

HEENT exam
Reassess the patient’s lung exam
Airway, Breathing, Circulation
Auscultation, percussion, and tactile fremitus
Order “Basic Labs”
CBC, CMP

## Slide 30
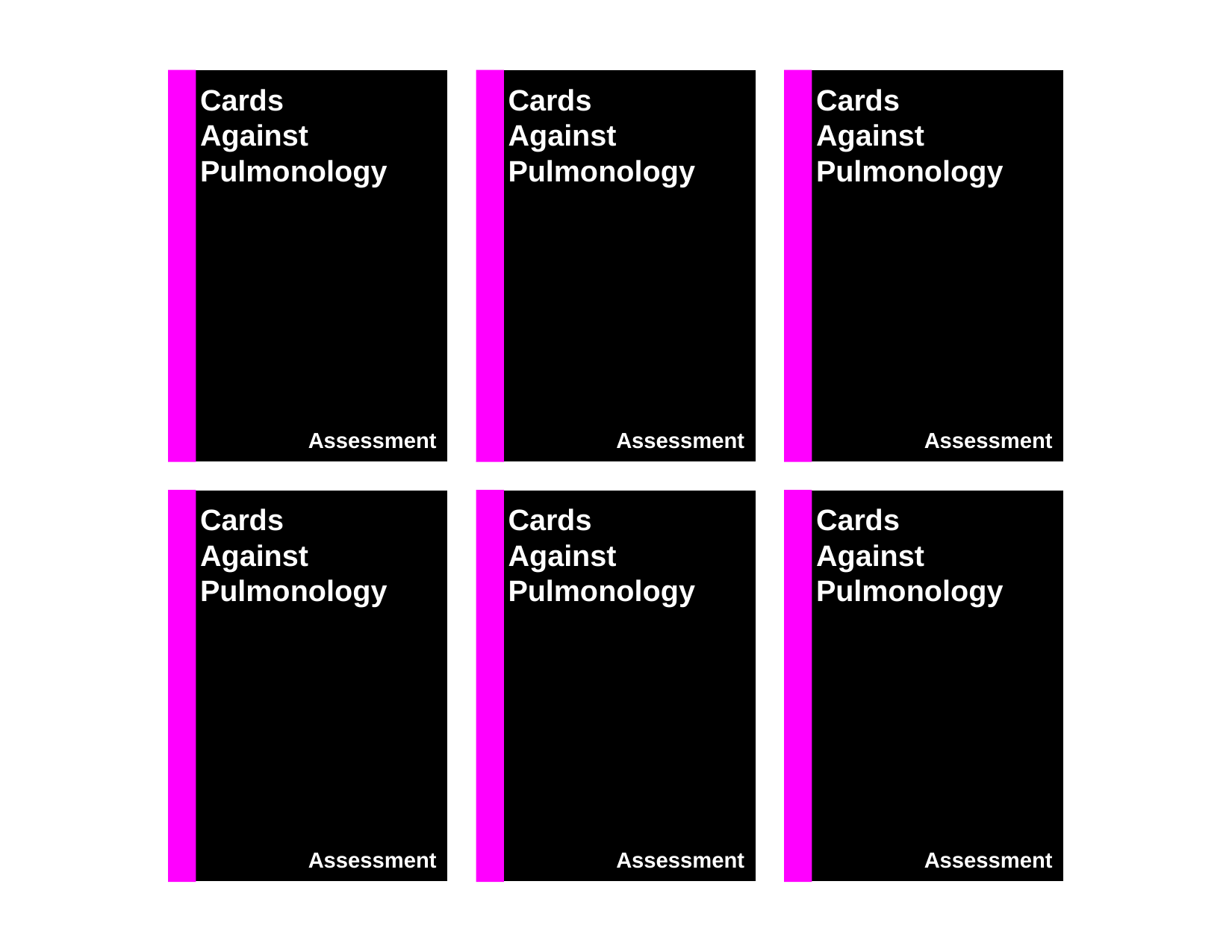

Cards
Against
Pulmonology
Assessment
Cards
Against
Pulmonology
Assessment
Cards
Against
Pulmonology
Assessment
Cards
Against
Pulmonology
Assessment
Cards
Against
Pulmonology
Assessment
Cards
Against
Pulmonology
Assessment

## Slide 31
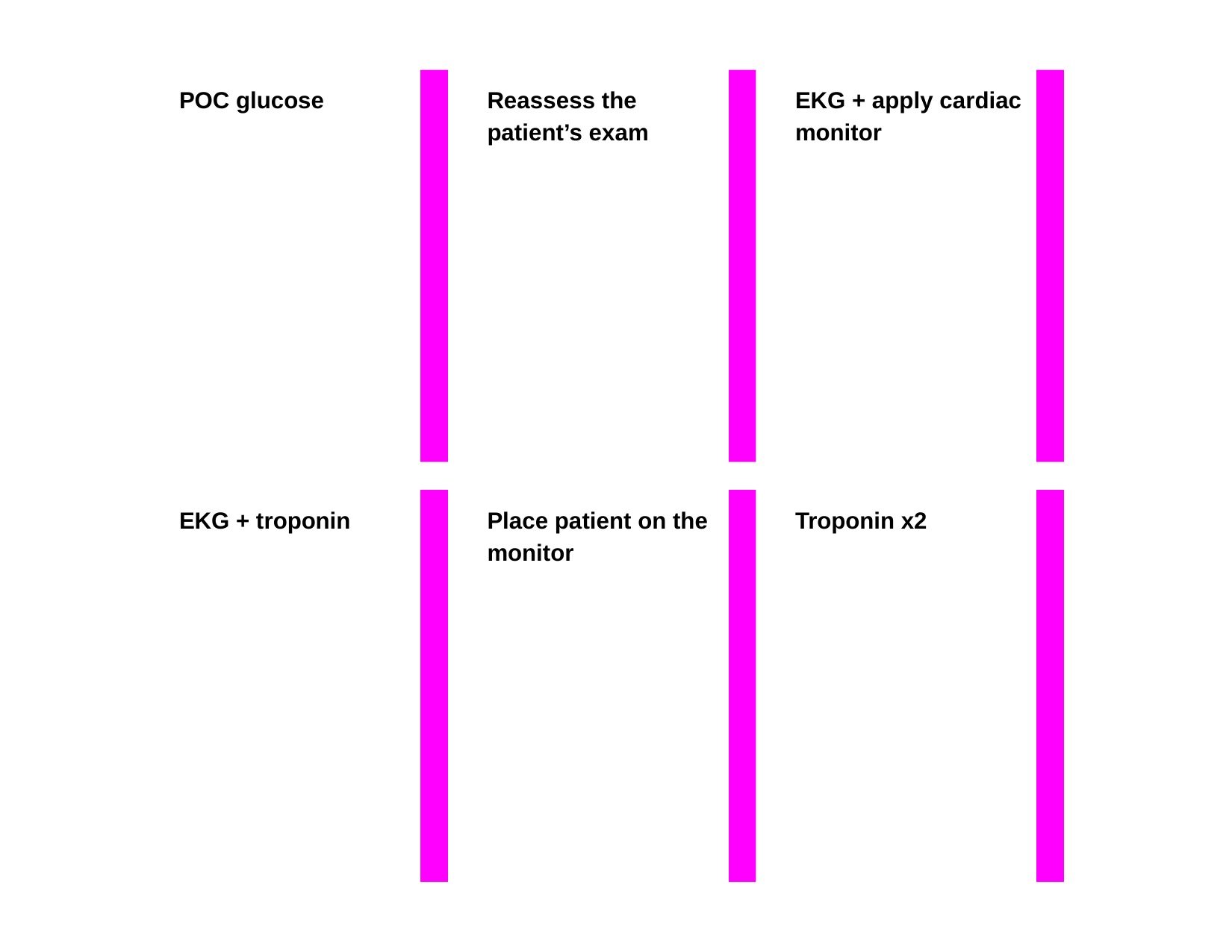

POC glucose
Reassess the patient’s exam
EKG + apply cardiac monitor
EKG + troponin
Place patient on the monitor
Troponin x2

## Slide 32
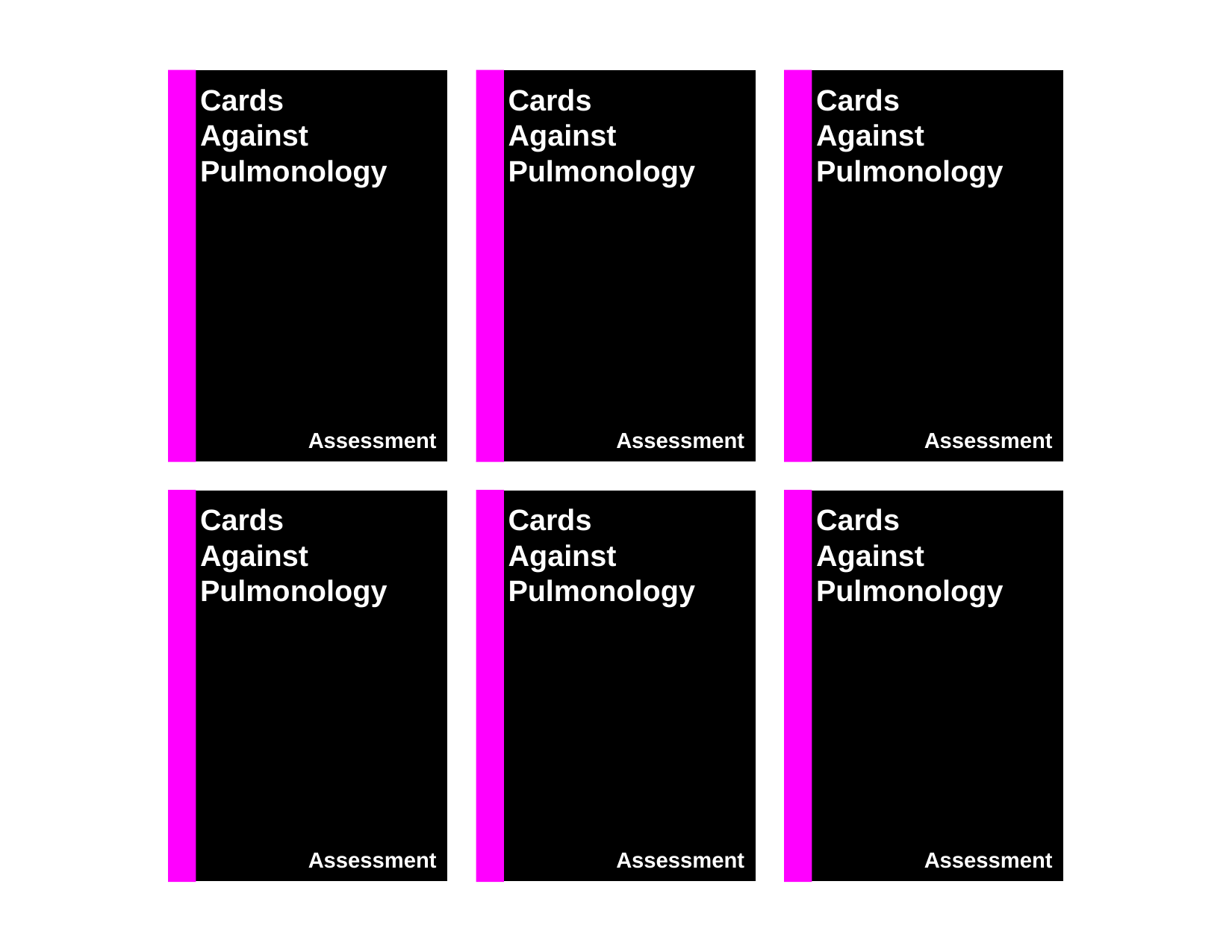

Cards
Against
Pulmonology
Assessment
Cards
Against
Pulmonology
Assessment
Cards
Against
Pulmonology
Assessment
Cards
Against
Pulmonology
Assessment
Cards
Against
Pulmonology
Assessment
Cards
Against
Pulmonology
Assessment

## Slide 33
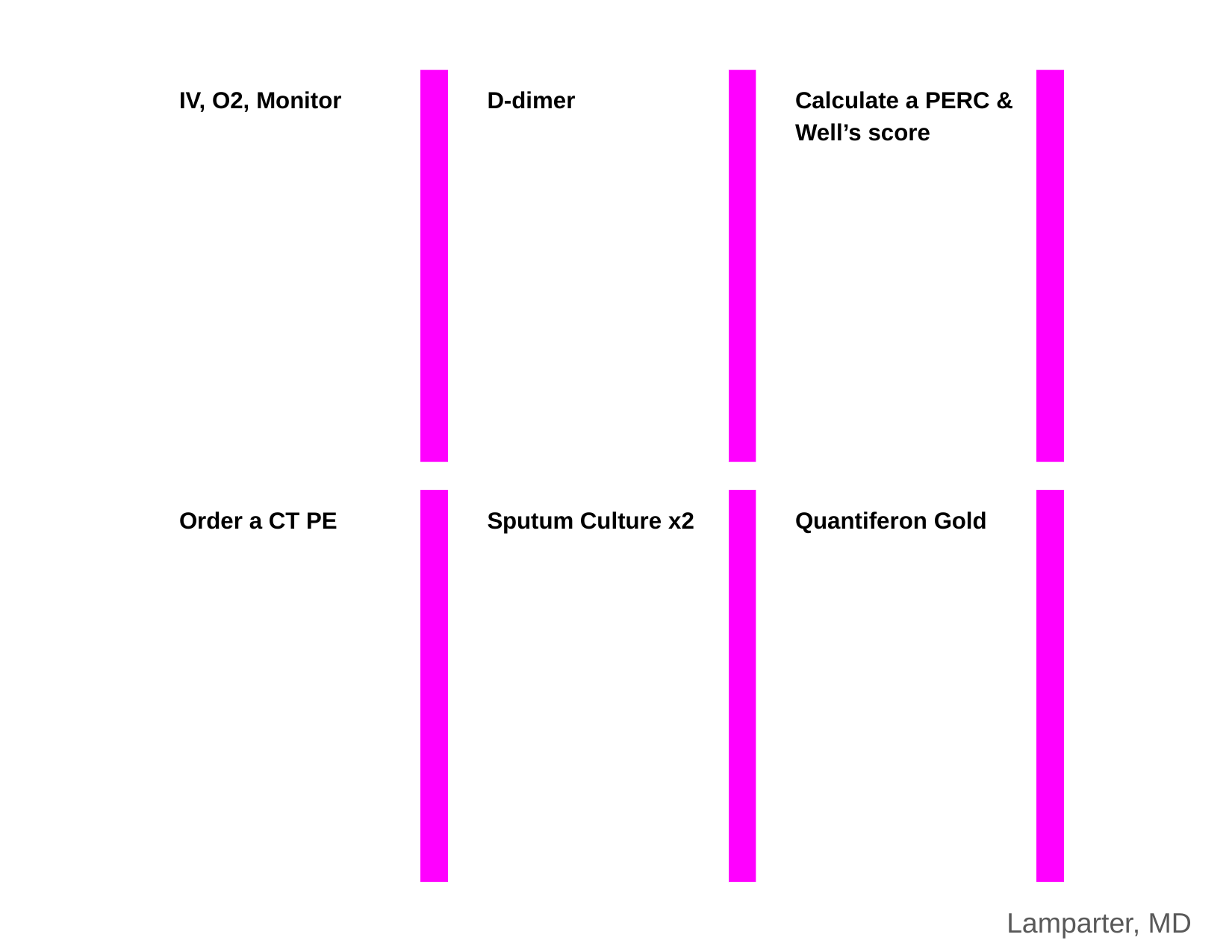

IV, O2, Monitor
D-dimer
Calculate a PERC & Well’s score
Order a CT PE
Sputum Culture x2
Quantiferon Gold
Lamparter, MD

## Slide 34
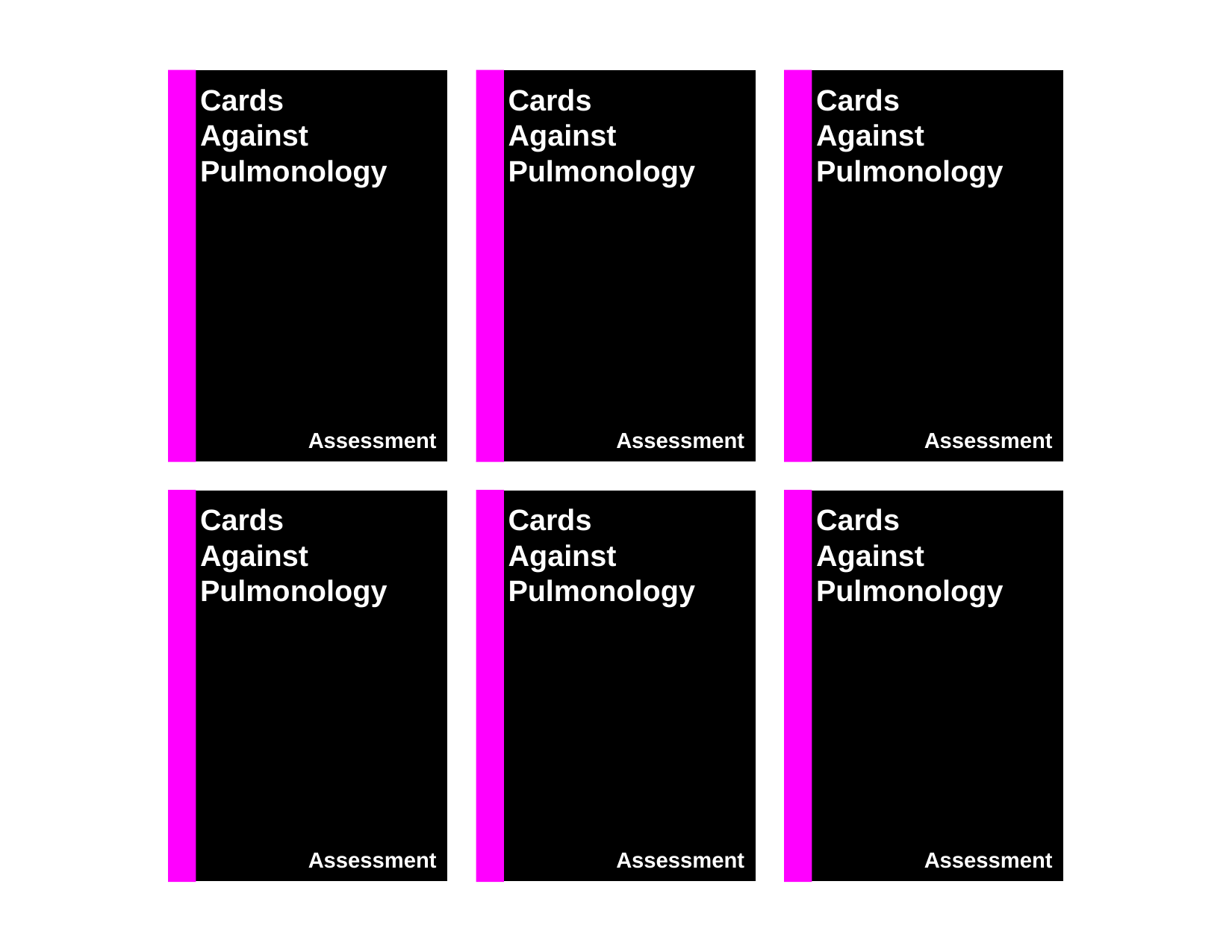

Cards
Against
Pulmonology
Assessment
Cards
Against
Pulmonology
Assessment
Cards
Against
Pulmonology
Assessment
Cards
Against
Pulmonology
Assessment
Cards
Against
Pulmonology
Assessment
Cards
Against
Pulmonology
Assessment

## Slide 35
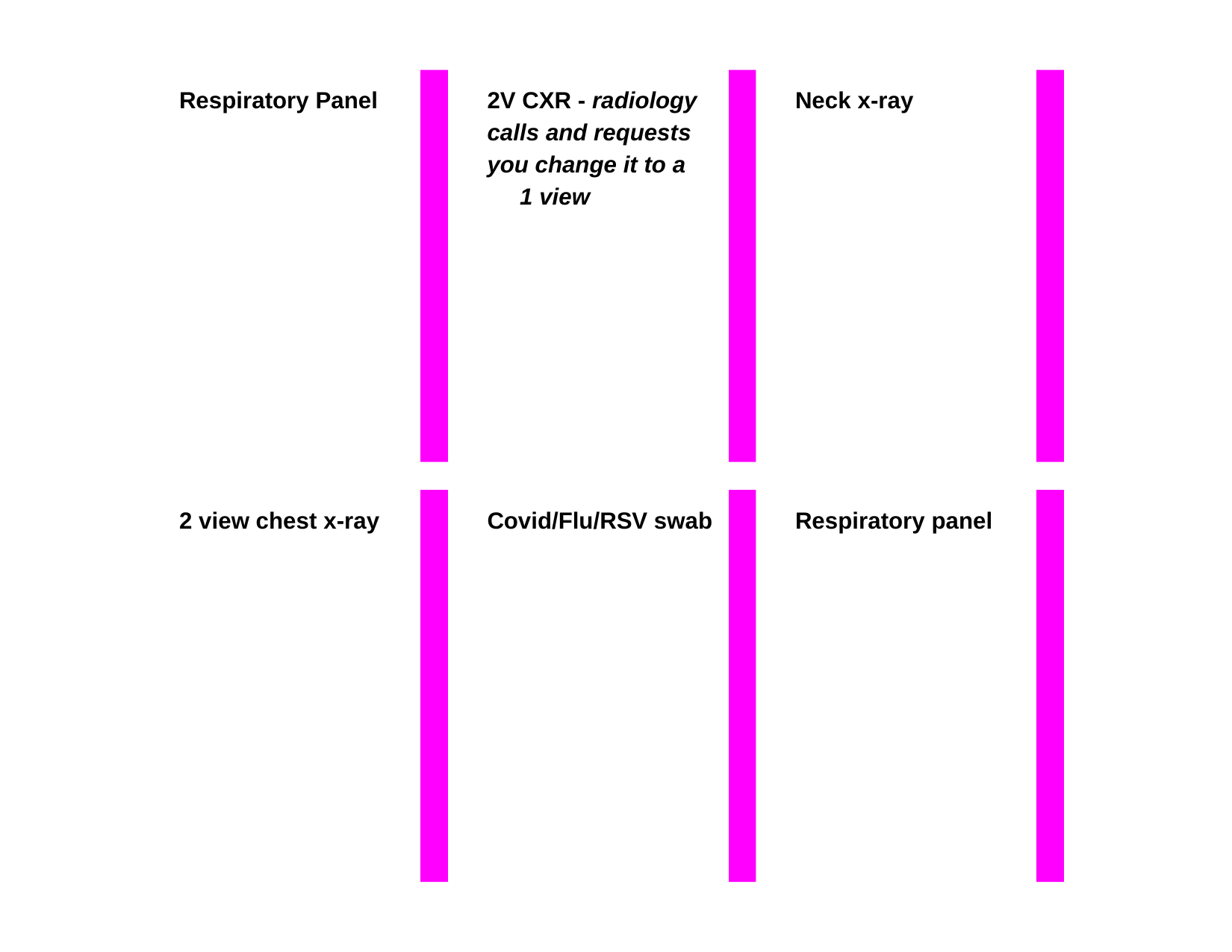

Respiratory Panel
2V CXR - radiology calls and requests you change it to a 1 view
Neck x-ray
2 view chest x-ray
Covid/Flu/RSV swab
Respiratory panel

## Slide 36
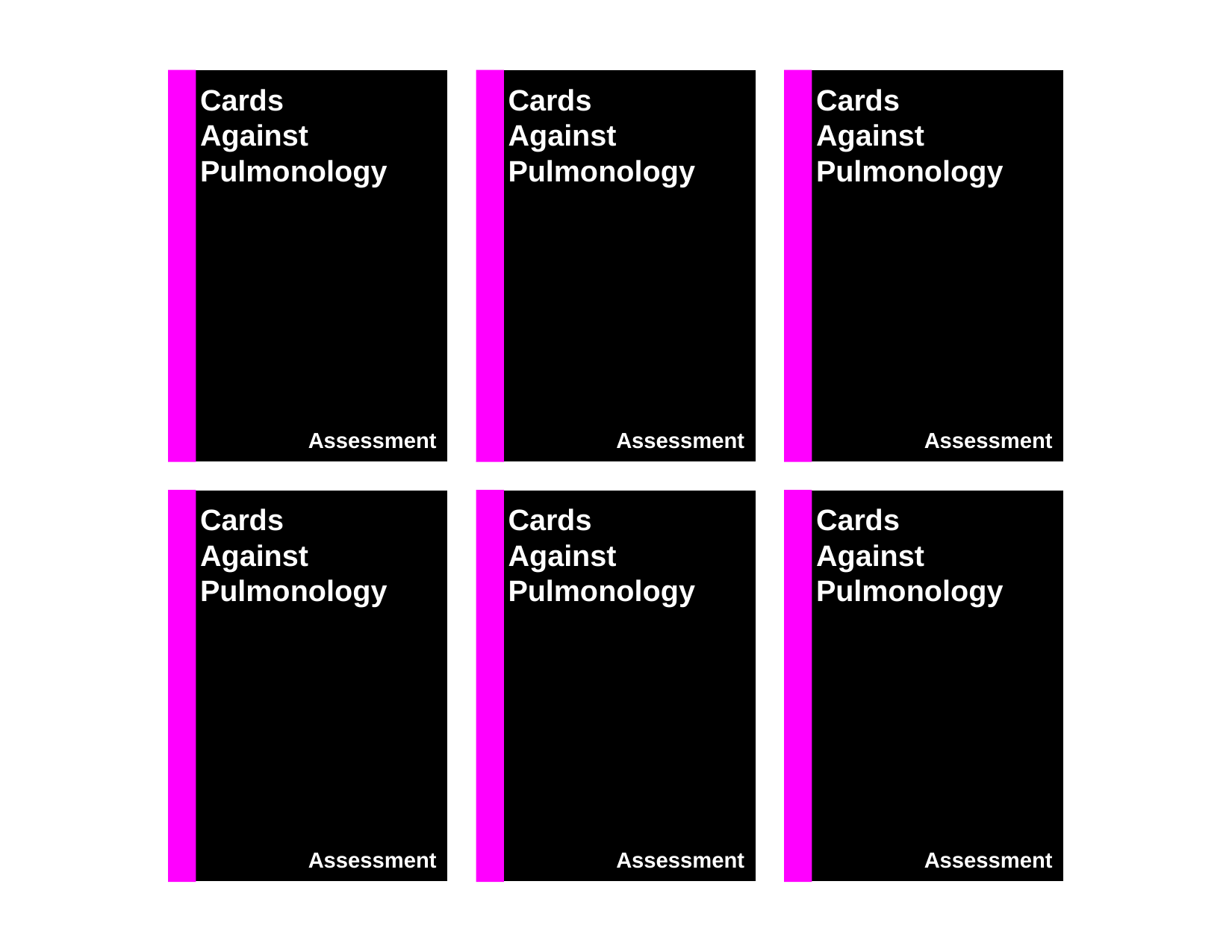

Cards
Against
Pulmonology
Assessment
Cards
Against
Pulmonology
Assessment
Cards
Against
Pulmonology
Assessment
Cards
Against
Pulmonology
Assessment
Cards
Against
Pulmonology
Assessment
Cards
Against
Pulmonology
Assessment

## Slide 37
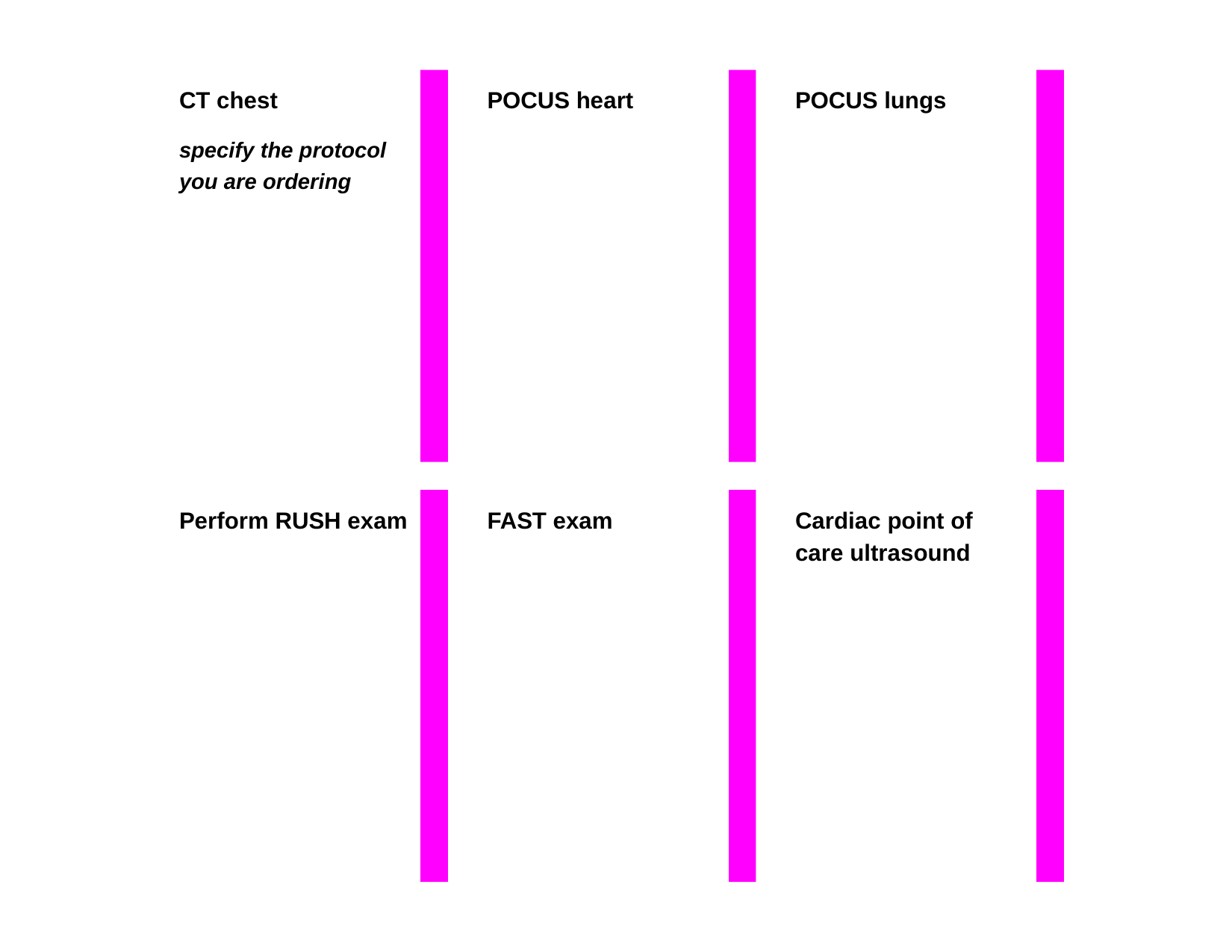

CT chest
specify the protocol you are ordering
POCUS heart
POCUS lungs
Perform RUSH exam
FAST exam
Cardiac point of care ultrasound

## Slide 38
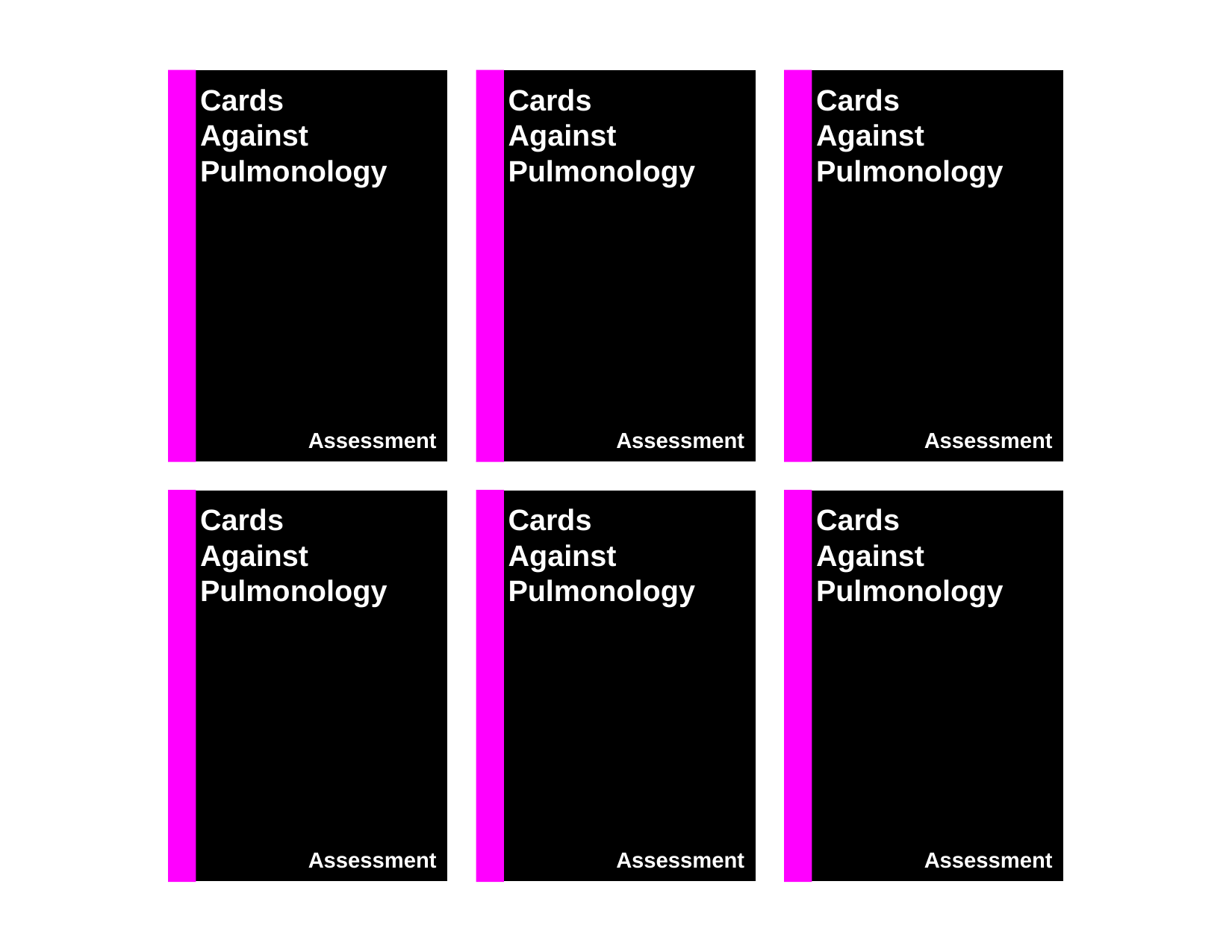

Cards
Against
Pulmonology
Assessment
Cards
Against
Pulmonology
Assessment
Cards
Against
Pulmonology
Assessment
Cards
Against
Pulmonology
Assessment
Cards
Against
Pulmonology
Assessment
Cards
Against
Pulmonology
Assessment

## Slide 39
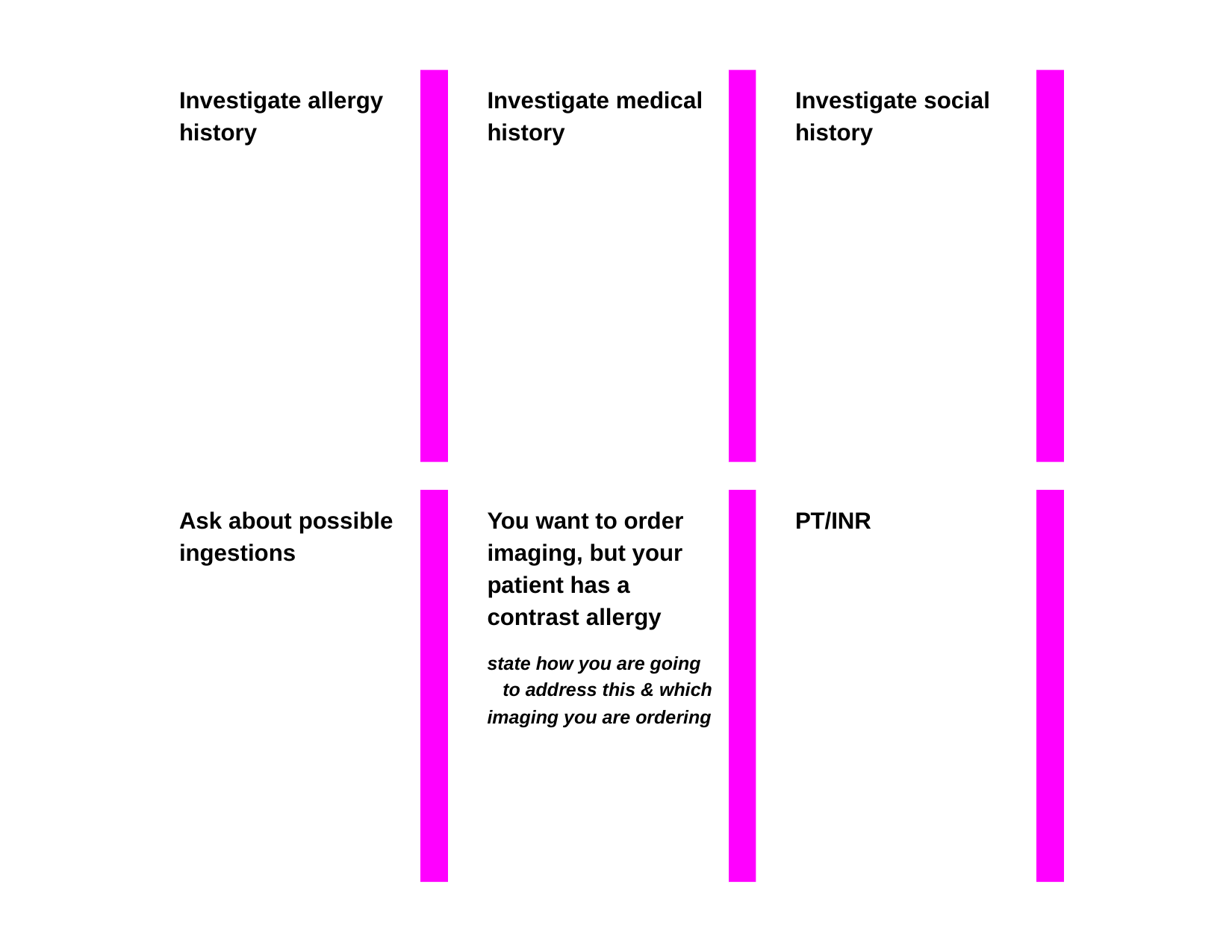

Investigate allergy history
Investigate medical history
Investigate social history
Ask about possible ingestions
You want to order imaging, but your patient has a contrast allergy
state how you are going to address this & which imaging you are ordering
PT/INR

## Slide 40
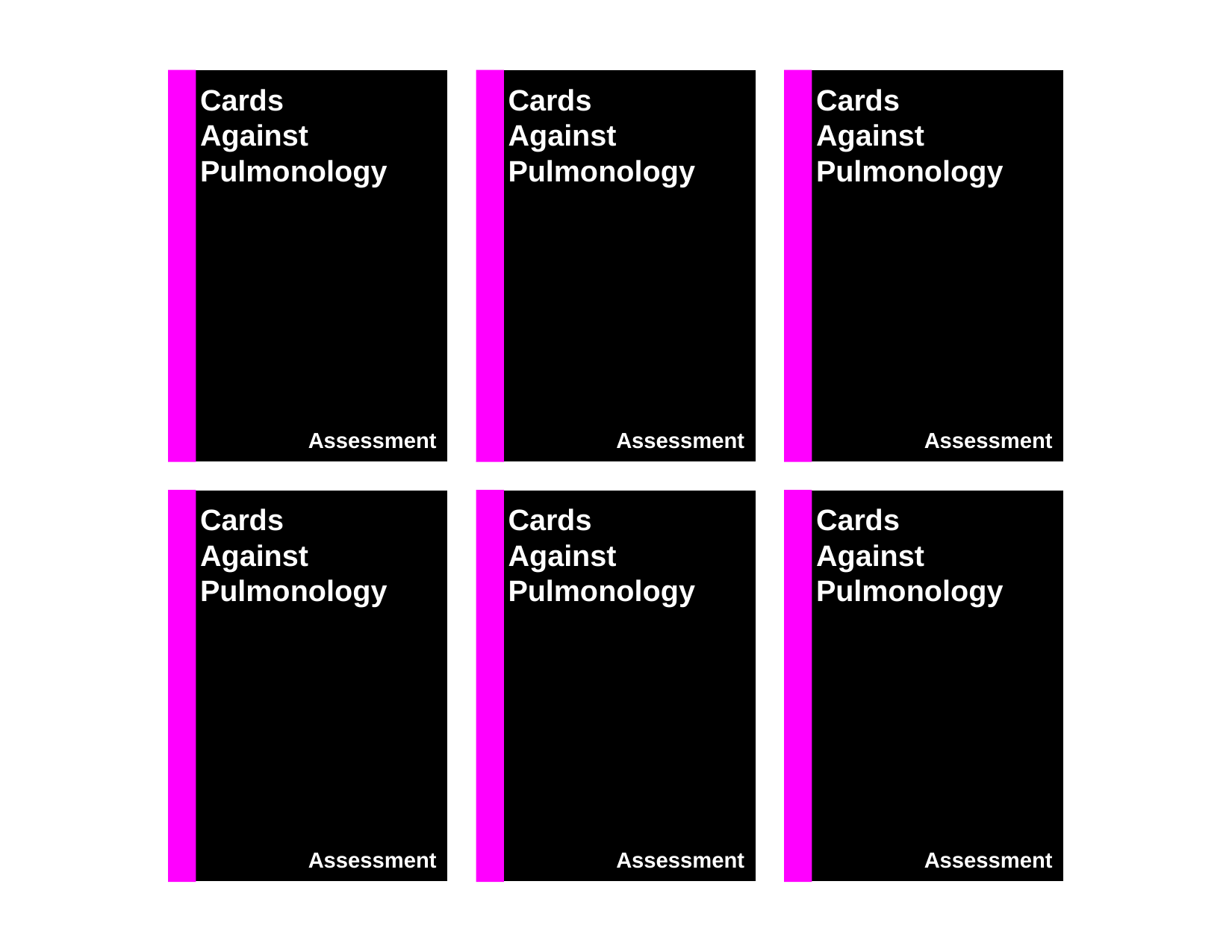

Cards
Against
Pulmonology
Assessment
Cards
Against
Pulmonology
Assessment
Cards
Against
Pulmonology
Assessment
Cards
Against
Pulmonology
Assessment
Cards
Against
Pulmonology
Assessment
Cards
Against
Pulmonology
Assessment

## Slide 41
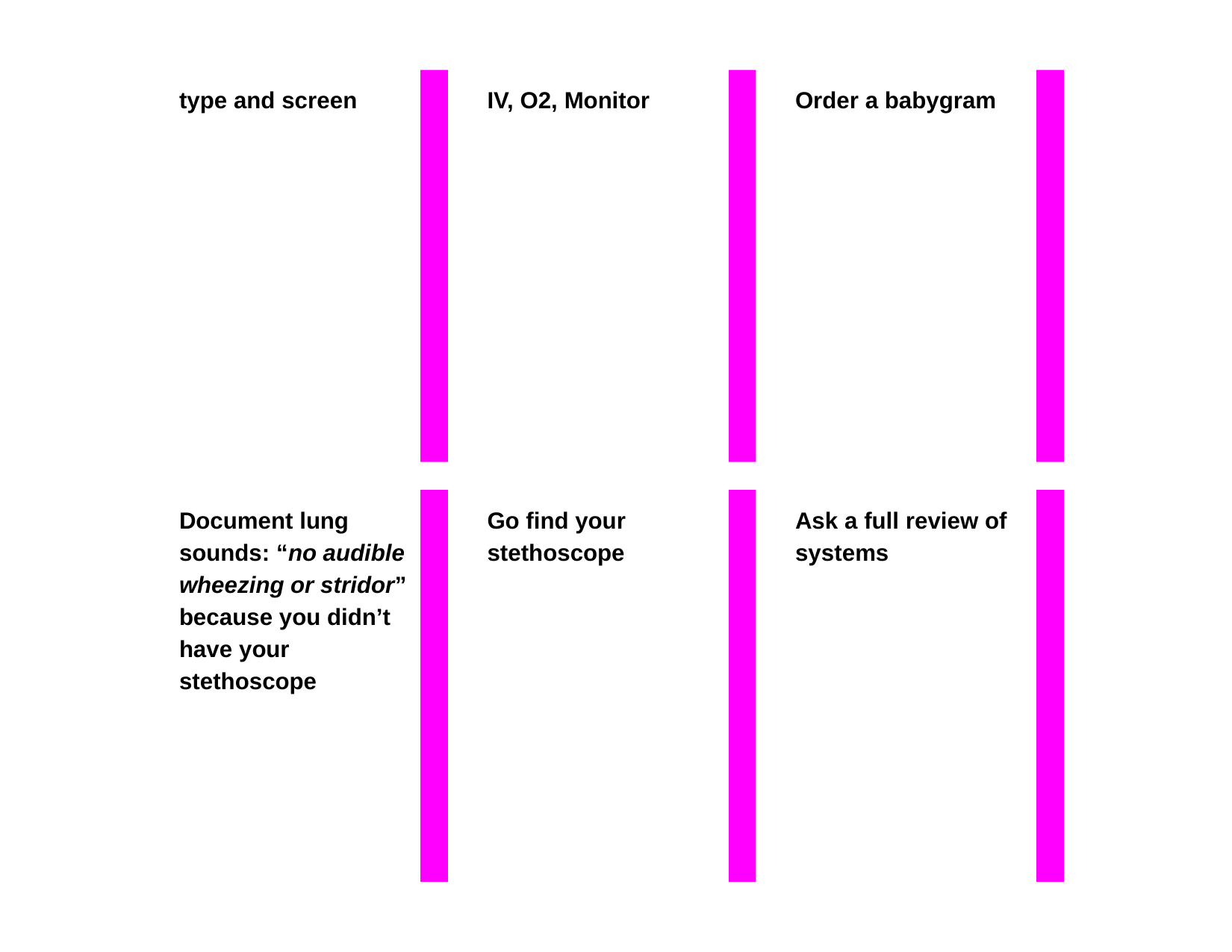

type and screen
IV, O2, Monitor
Order a babygram
Document lung sounds: “no audible wheezing or stridor” because you didn’t have your stethoscope
Go find your stethoscope
Ask a full review of systems

## Slide 42
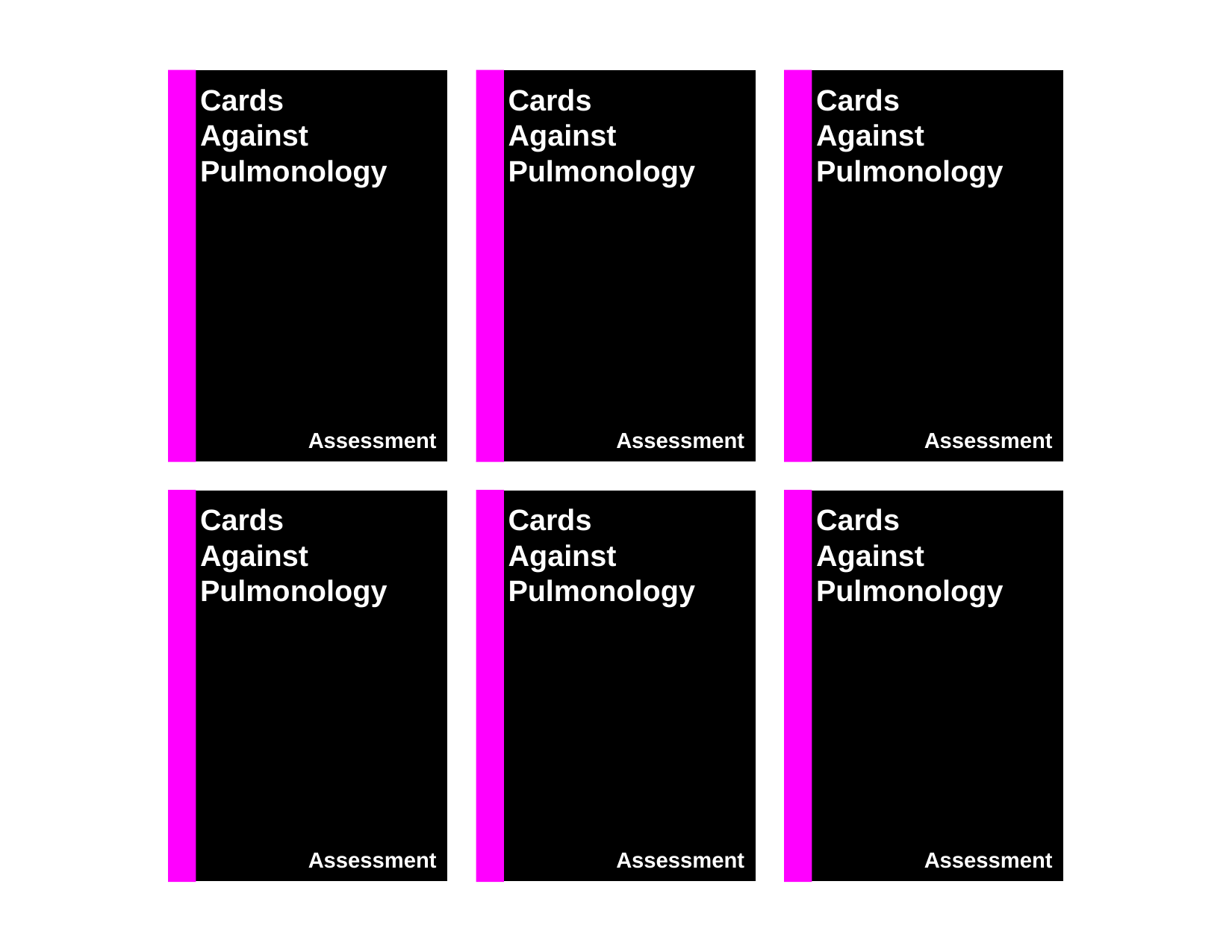

Cards
Against
Pulmonology
Assessment
Cards
Against
Pulmonology
Assessment
Cards
Against
Pulmonology
Assessment
Cards
Against
Pulmonology
Assessment
Cards
Against
Pulmonology
Assessment
Cards
Against
Pulmonology
Assessment

## Slide 43
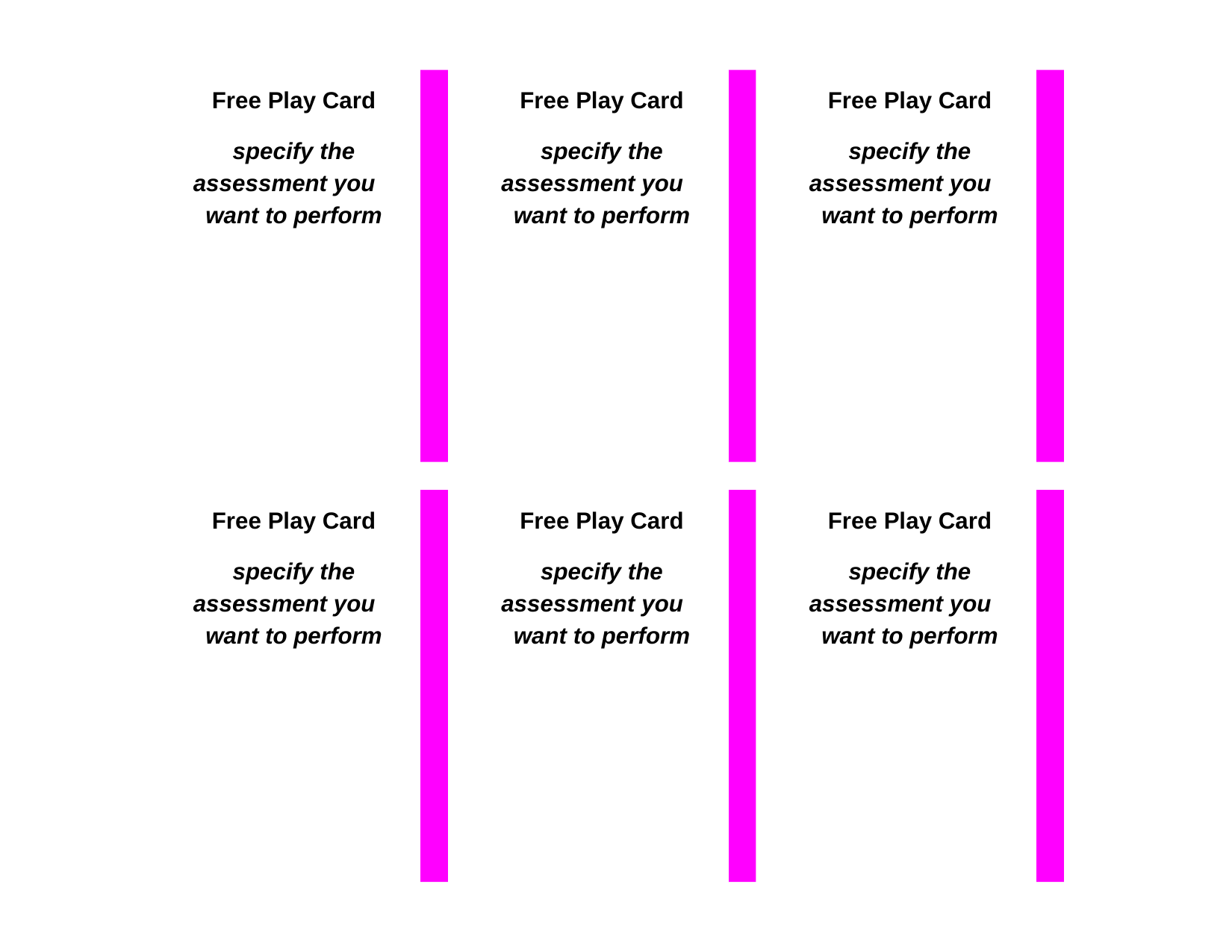

Free Play Card
specify the assessment you want to perform
Free Play Card
specify the assessment you want to perform
Free Play Card
specify the assessment you want to perform
Free Play Card
specify the assessment you want to perform
Free Play Card
specify the assessment you want to perform
Free Play Card
specify the assessment you want to perform

## Slide 44
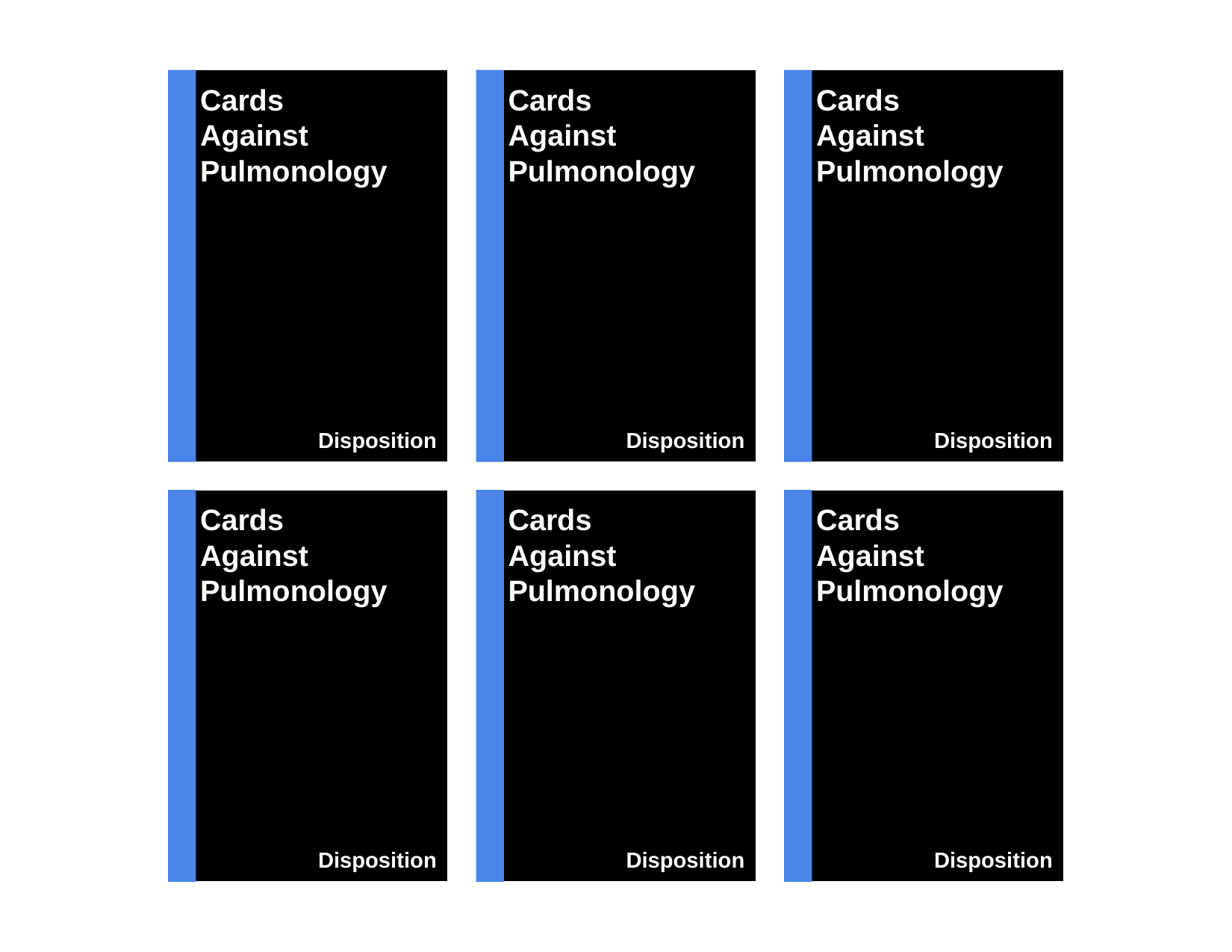

Cards
Against
Pulmonology
Disposition
Cards
Against
Pulmonology
Disposition
Cards
Against
Pulmonology
Disposition
Cards
Against
Pulmonology
Disposition
Cards
Against
Pulmonology
Disposition
Cards
Against
Pulmonology
Disposition

## Slide 45
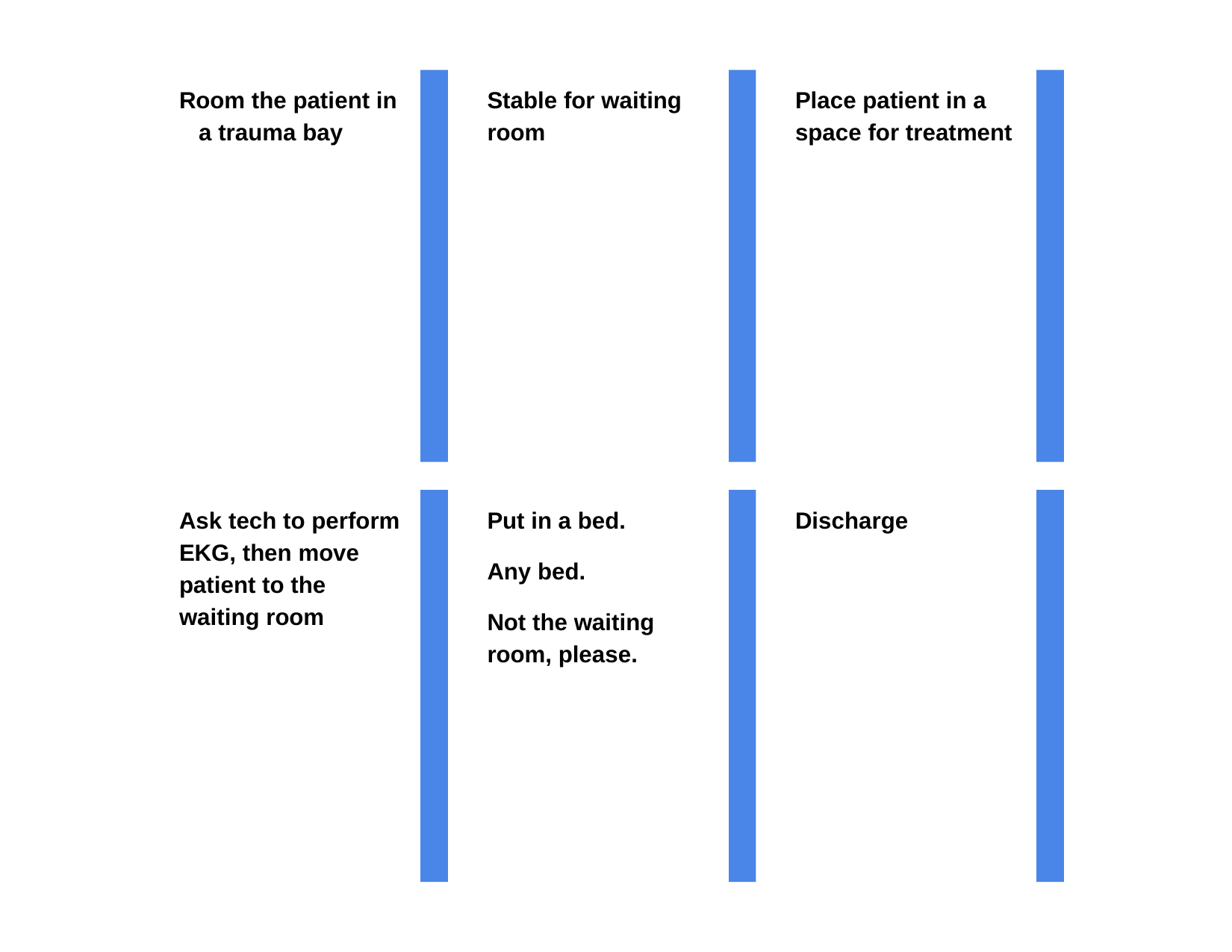

Room the patient in a trauma bay
Stable for waiting room
Place patient in a space for treatment
Ask tech to perform EKG, then move patient to the waiting room
Put in a bed.
Any bed.
Not the waiting room, please.
Discharge

## Slide 46
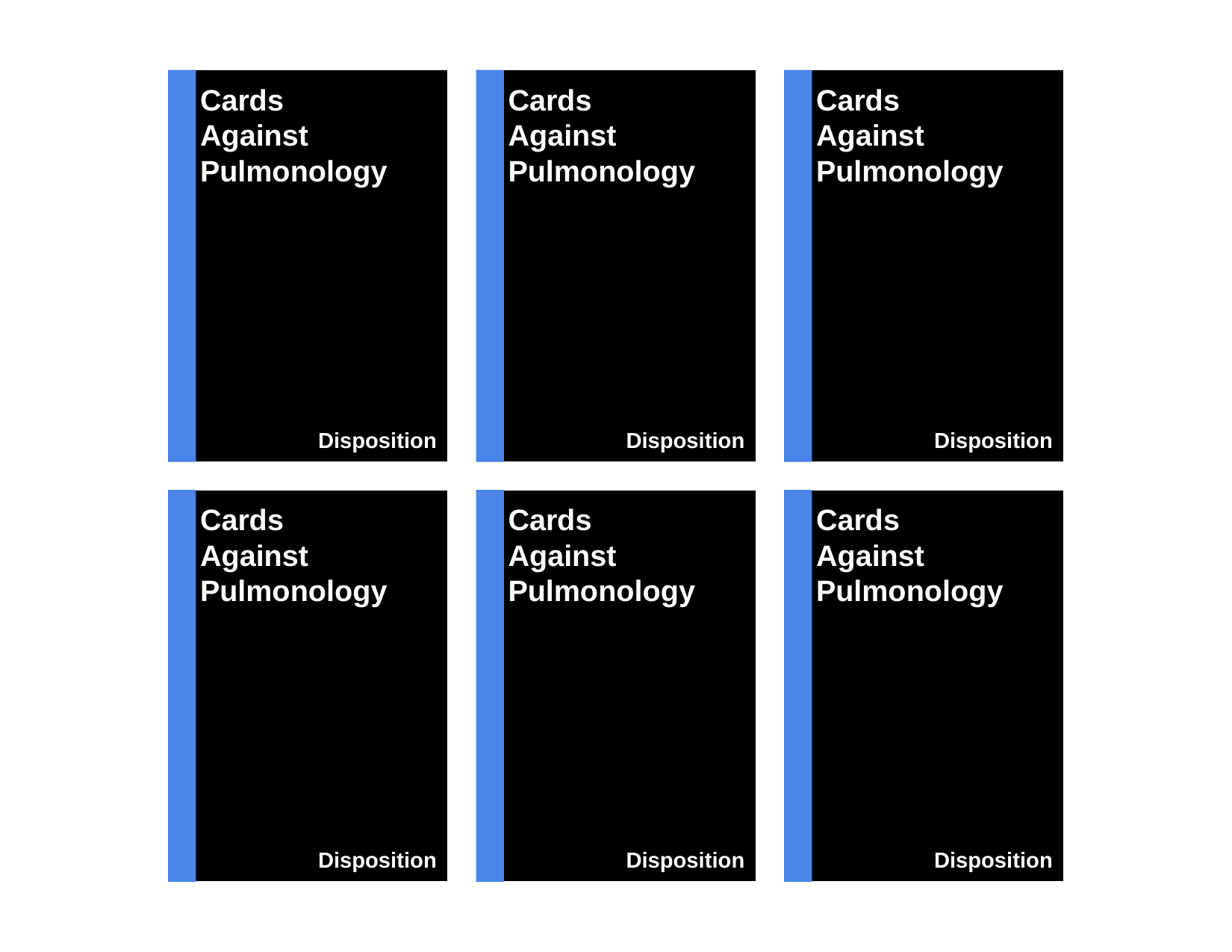

Cards
Against
Pulmonology
Disposition
Cards
Against
Pulmonology
Disposition
Cards
Against
Pulmonology
Disposition
Cards
Against
Pulmonology
Disposition
Cards
Against
Pulmonology
Disposition
Cards
Against
Pulmonology
Disposition

## Slide 47
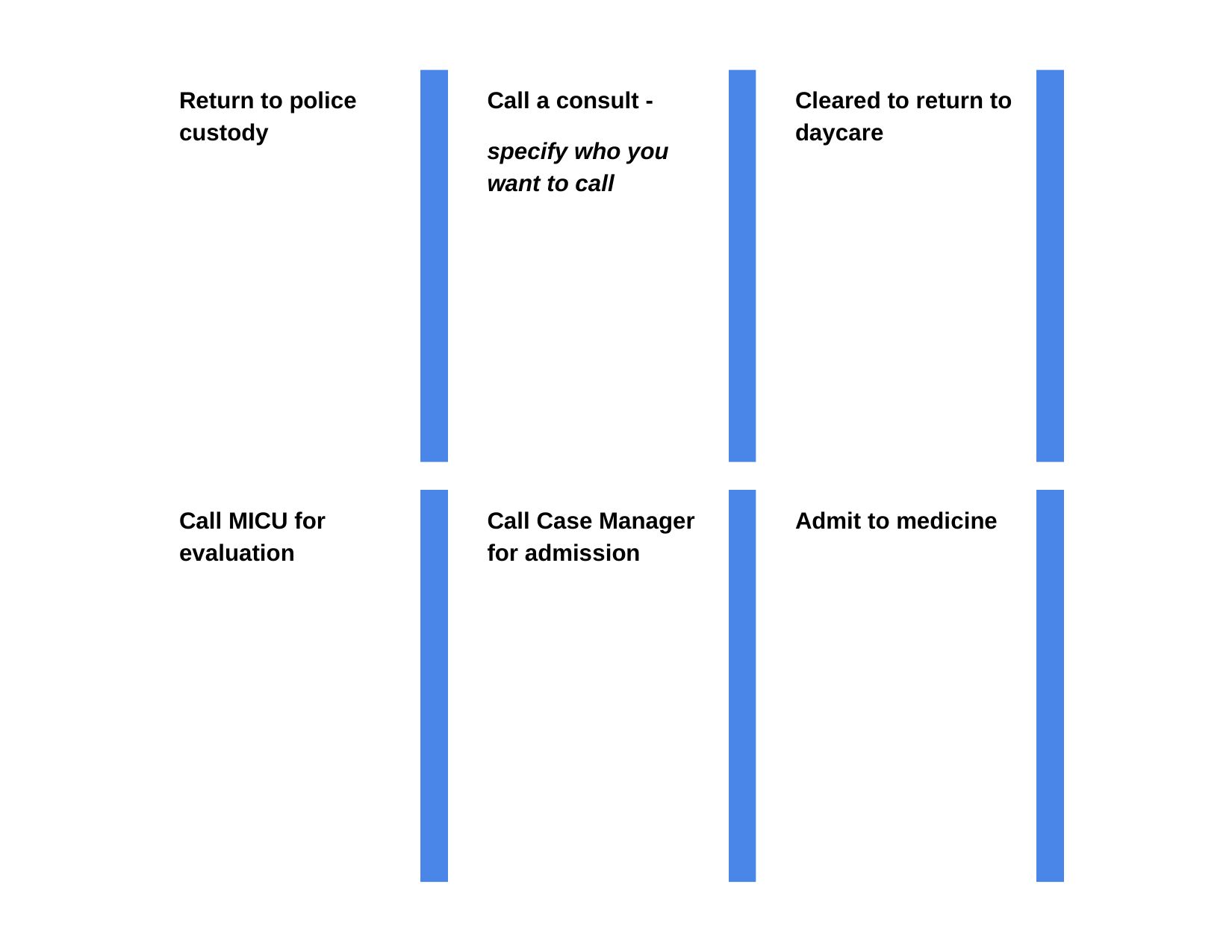

Return to police custody
Call a consult -
specify who you want to call
Cleared to return to daycare
Call MICU for evaluation
Call Case Manager for admission
Admit to medicine

## Slide 48
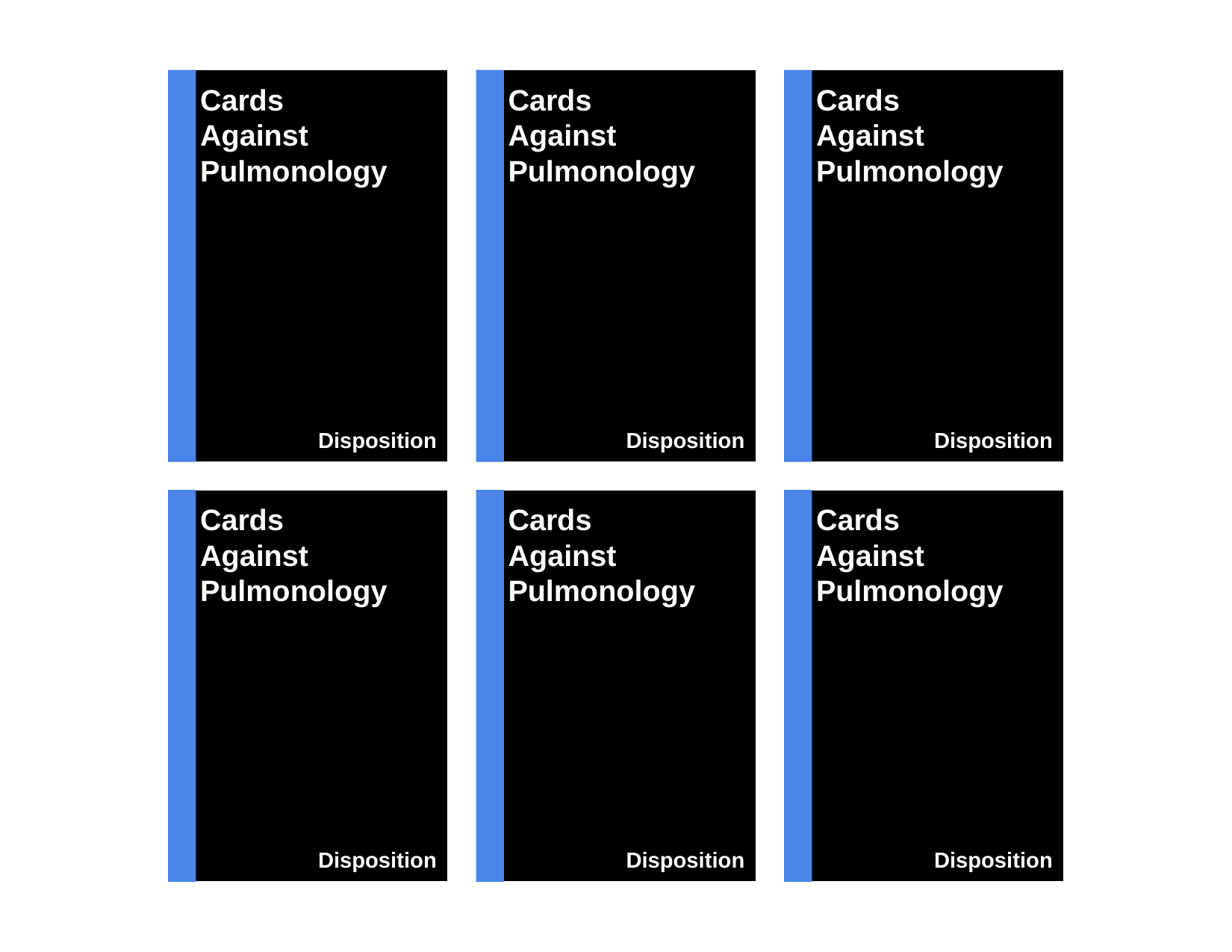

Cards
Against
Pulmonology
Disposition
Cards
Against
Pulmonology
Disposition
Cards
Against
Pulmonology
Disposition
Cards
Against
Pulmonology
Disposition
Cards
Against
Pulmonology
Disposition
Cards
Against
Pulmonology
Disposition

## Slide 49
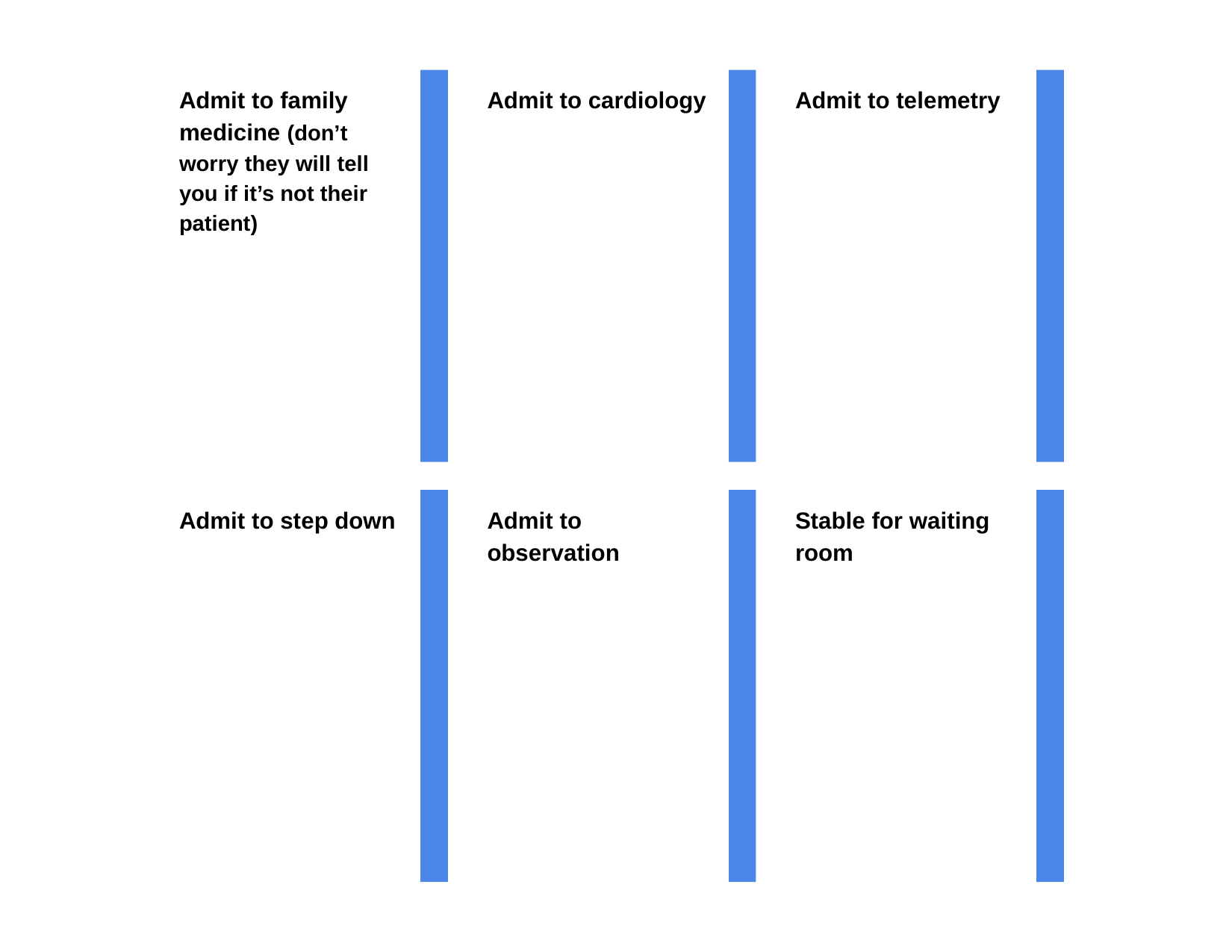

Admit to family medicine (don’t worry they will tell you if it’s not their patient)
Admit to cardiology
Admit to telemetry
Admit to step down
Admit to observation
Stable for waiting room

## Slide 50
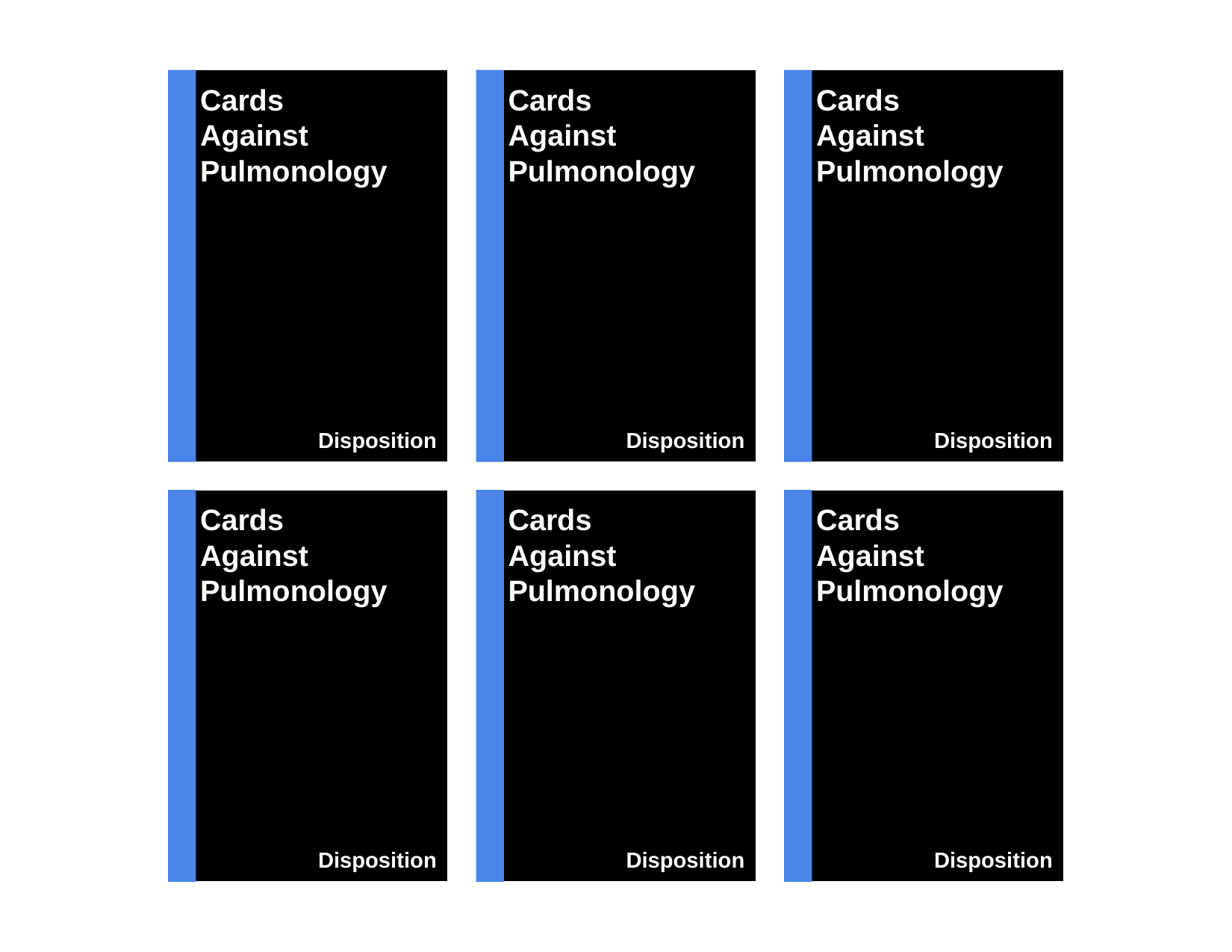

Cards
Against
Pulmonology
Disposition
Cards
Against
Pulmonology
Disposition
Cards
Against
Pulmonology
Disposition
Cards
Against
Pulmonology
Disposition
Cards
Against
Pulmonology
Disposition
Cards
Against
Pulmonology
Disposition

## Slide 51
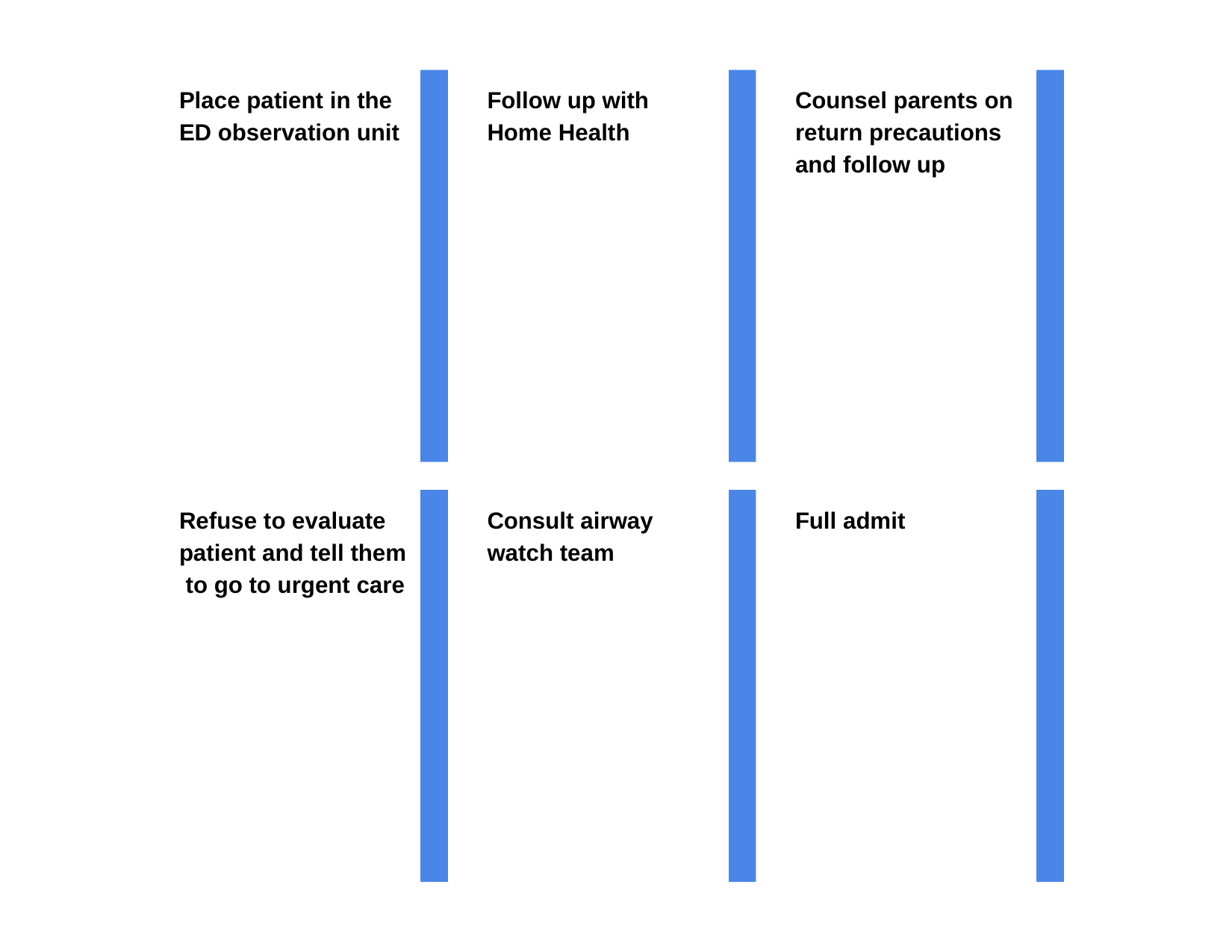

Place patient in the ED observation unit
Follow up with Home Health
Counsel parents on return precautions and follow up
Refuse to evaluate patient and tell them to go to urgent care
Consult airway watch team
Full admit

## Slide 52
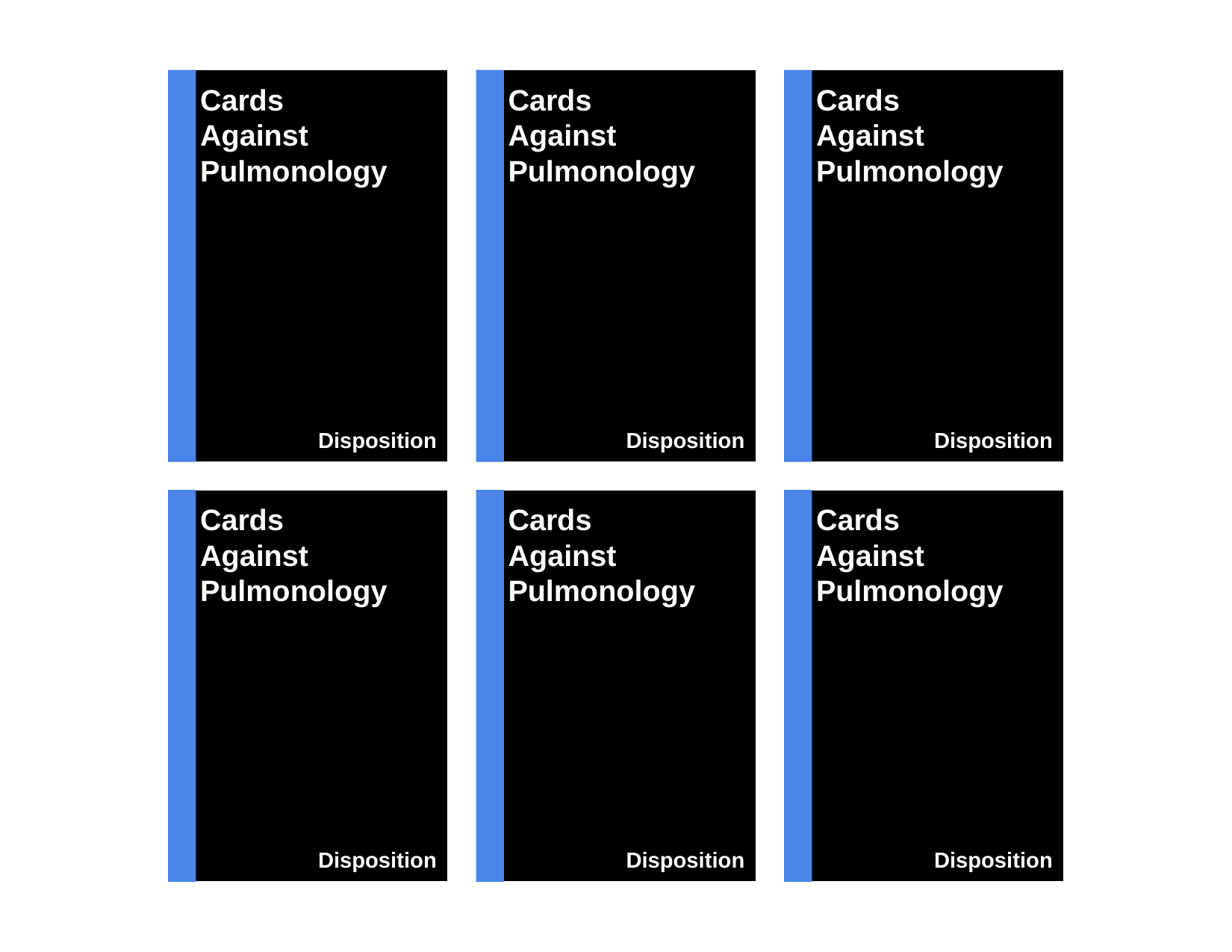

Cards
Against
Pulmonology
Disposition
Cards
Against
Pulmonology
Disposition
Cards
Against
Pulmonology
Disposition
Cards
Against
Pulmonology
Disposition
Cards
Against
Pulmonology
Disposition
Cards
Against
Pulmonology
Disposition

## Slide 53
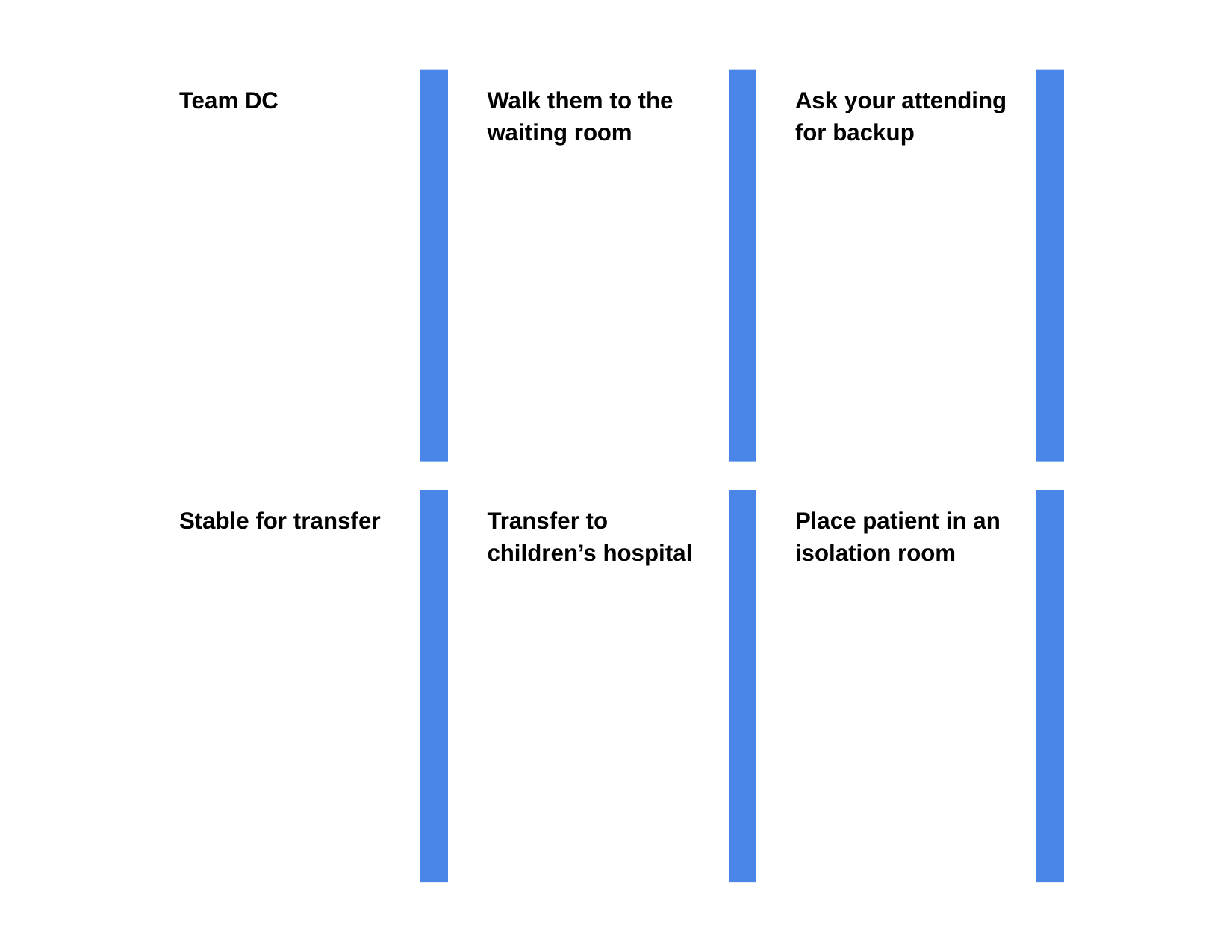

Team DC
Walk them to the waiting room
Ask your attending for backup
Stable for transfer
Transfer to children’s hospital
Place patient in an isolation room

## Slide 54
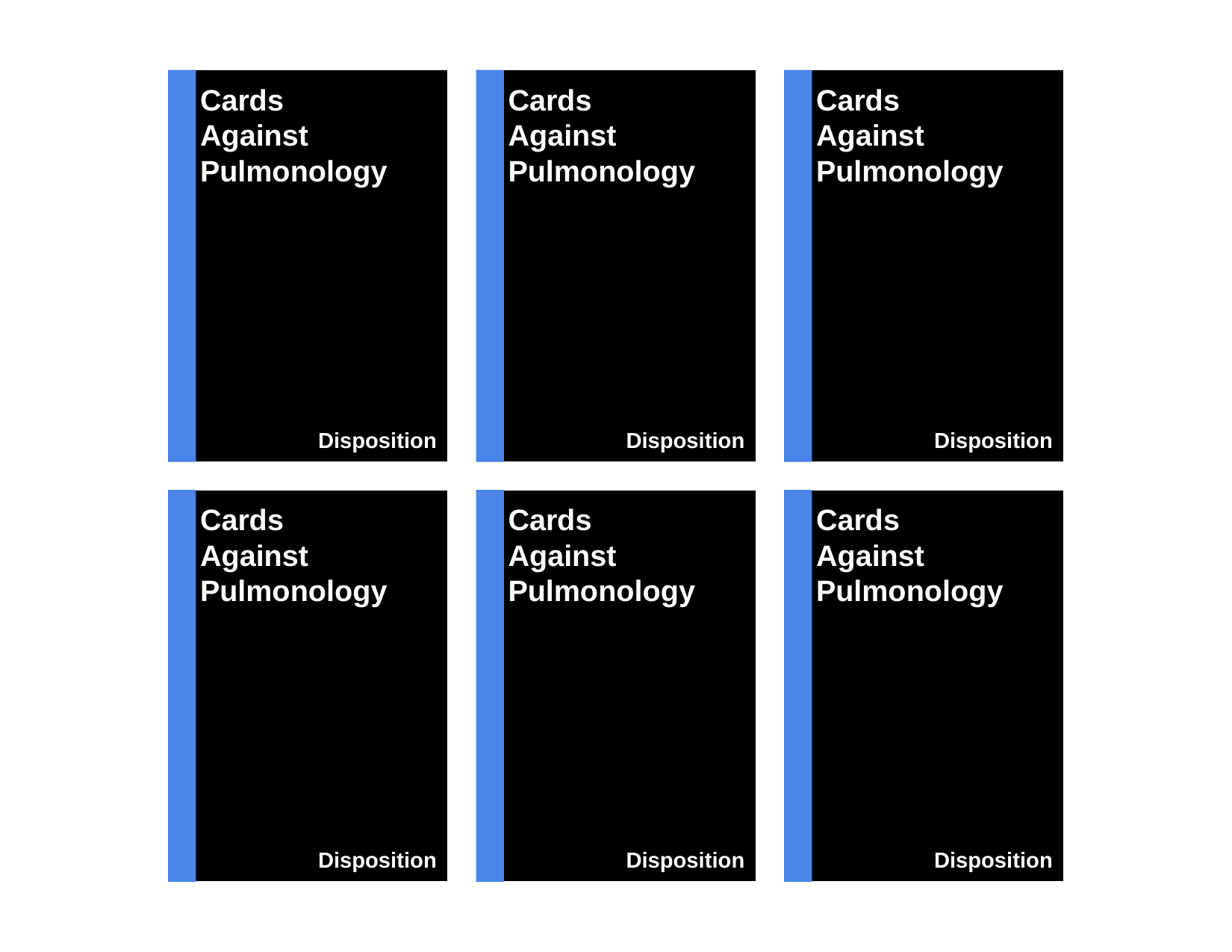

Cards
Against
Pulmonology
Disposition
Cards
Against
Pulmonology
Disposition
Cards
Against
Pulmonology
Disposition
Cards
Against
Pulmonology
Disposition
Cards
Against
Pulmonology
Disposition
Cards
Against
Pulmonology
Disposition

## Slide 55
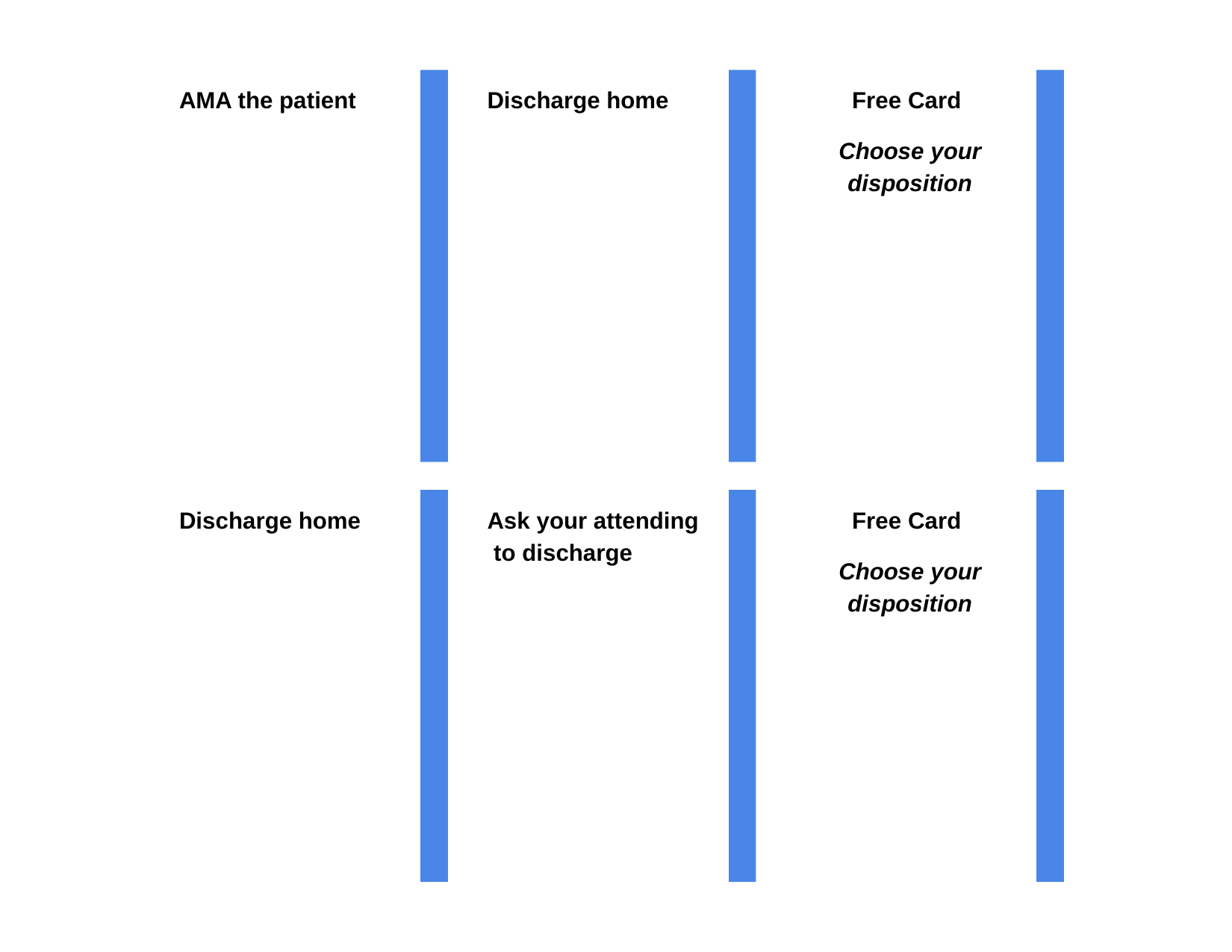

AMA the patient
Discharge home
Free Card
Choose your disposition
Discharge home
Ask your attending to discharge
Free Card
Choose your disposition

## Slide 56
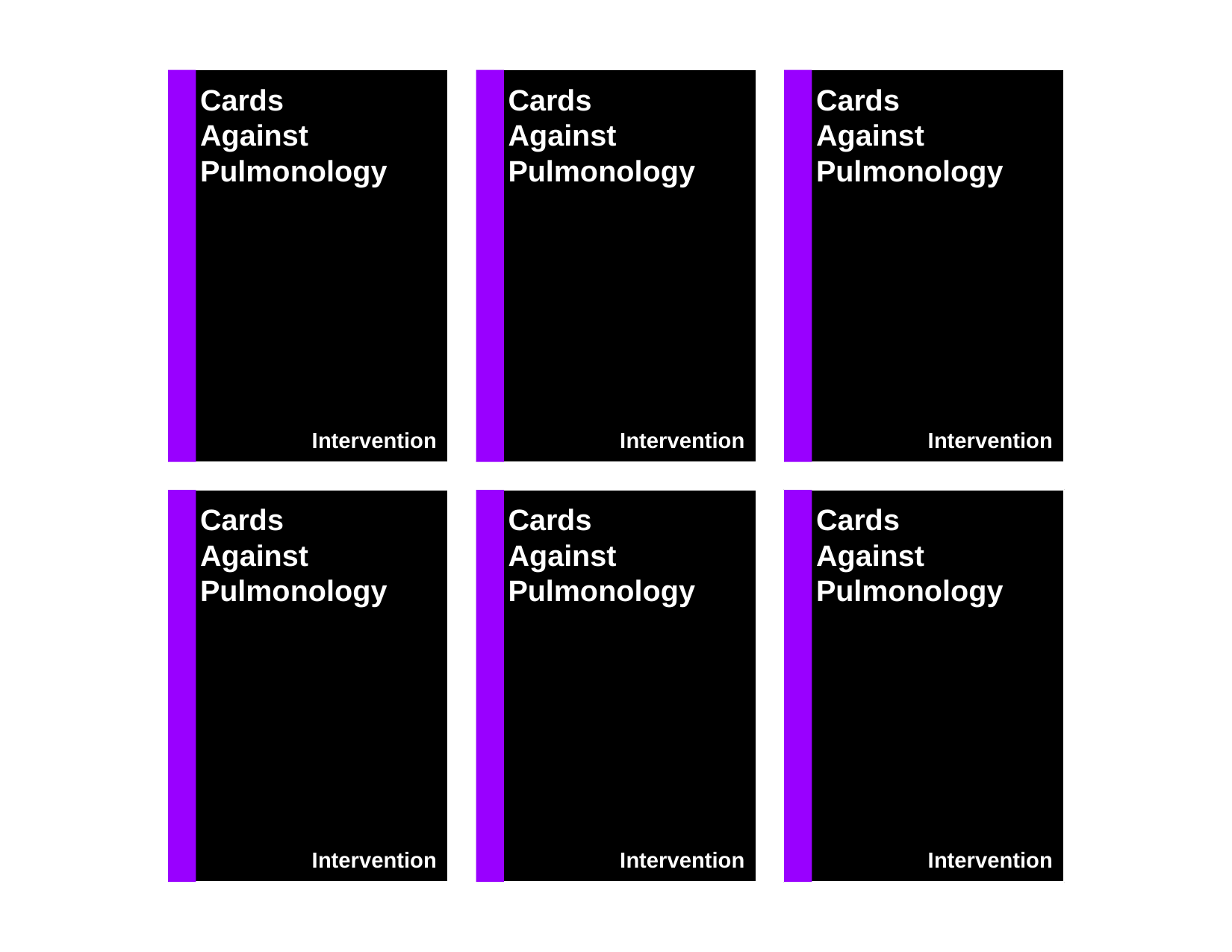

Cards
Against
Pulmonology
Intervention
Cards
Against
Pulmonology
Intervention
Cards
Against
Pulmonology
Intervention
Cards
Against
Pulmonology
Intervention
Cards
Against
Pulmonology
Intervention
Cards
Against
Pulmonology
Intervention

## Slide 57
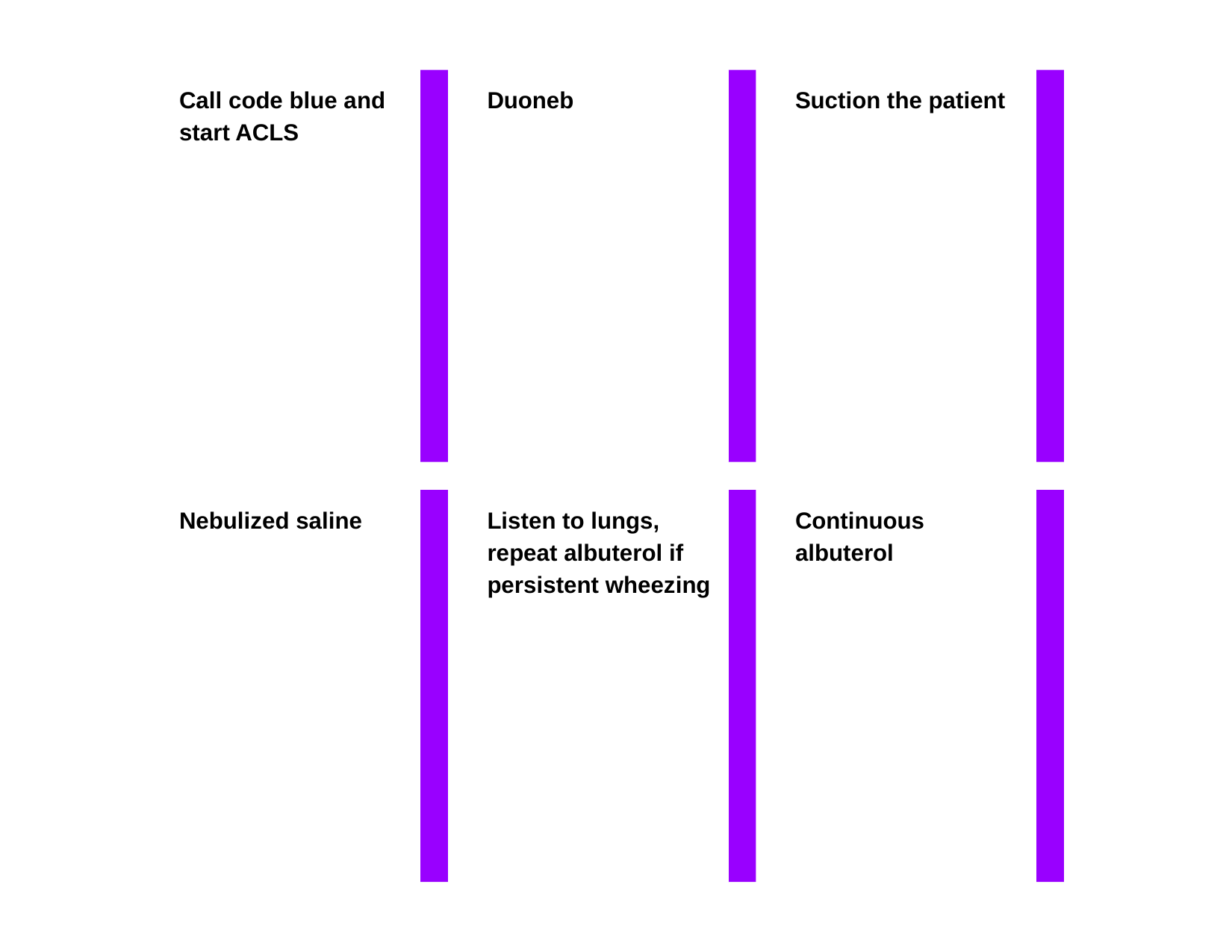

Call code blue and start ACLS
Duoneb
Suction the patient
Nebulized saline
Listen to lungs, repeat albuterol if persistent wheezing
Continuous albuterol

## Slide 58
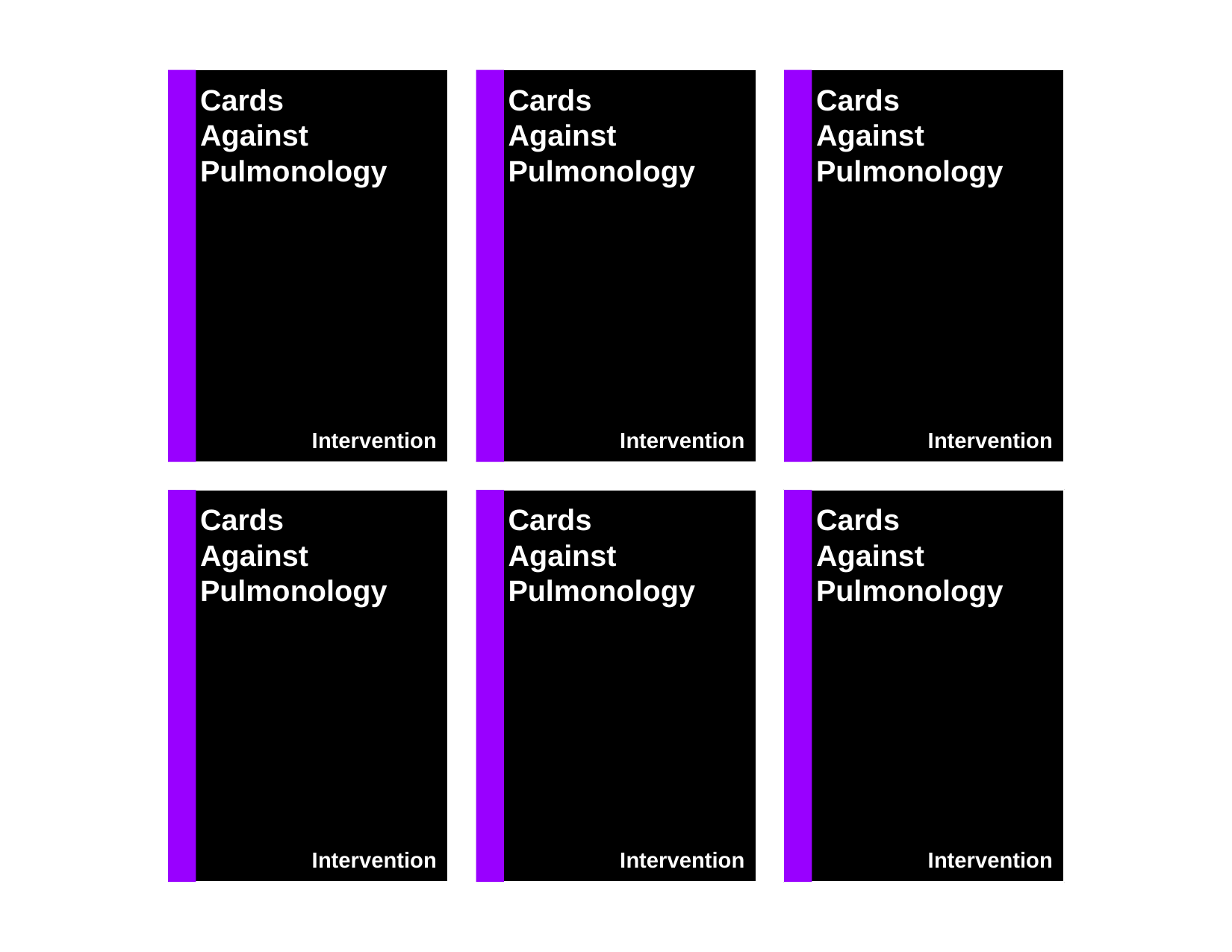

Cards
Against
Pulmonology
Intervention
Cards
Against
Pulmonology
Intervention
Cards
Against
Pulmonology
Intervention
Cards
Against
Pulmonology
Intervention
Cards
Against
Pulmonology
Intervention
Cards
Against
Pulmonology
Intervention

## Slide 59
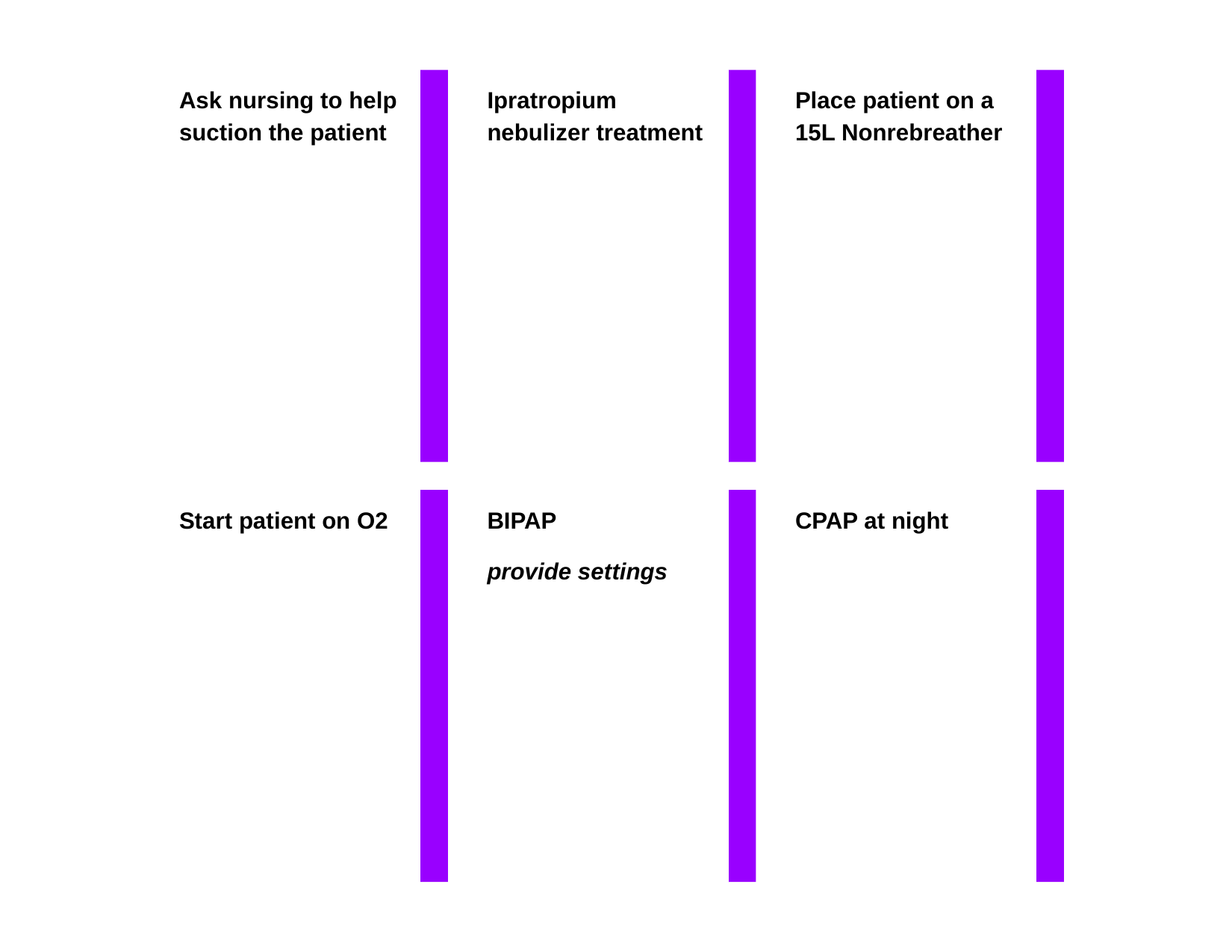

Ask nursing to help suction the patient
Ipratropium nebulizer treatment
Place patient on a 15L Nonrebreather
Start patient on O2
BIPAP
provide settings
CPAP at night

## Slide 60
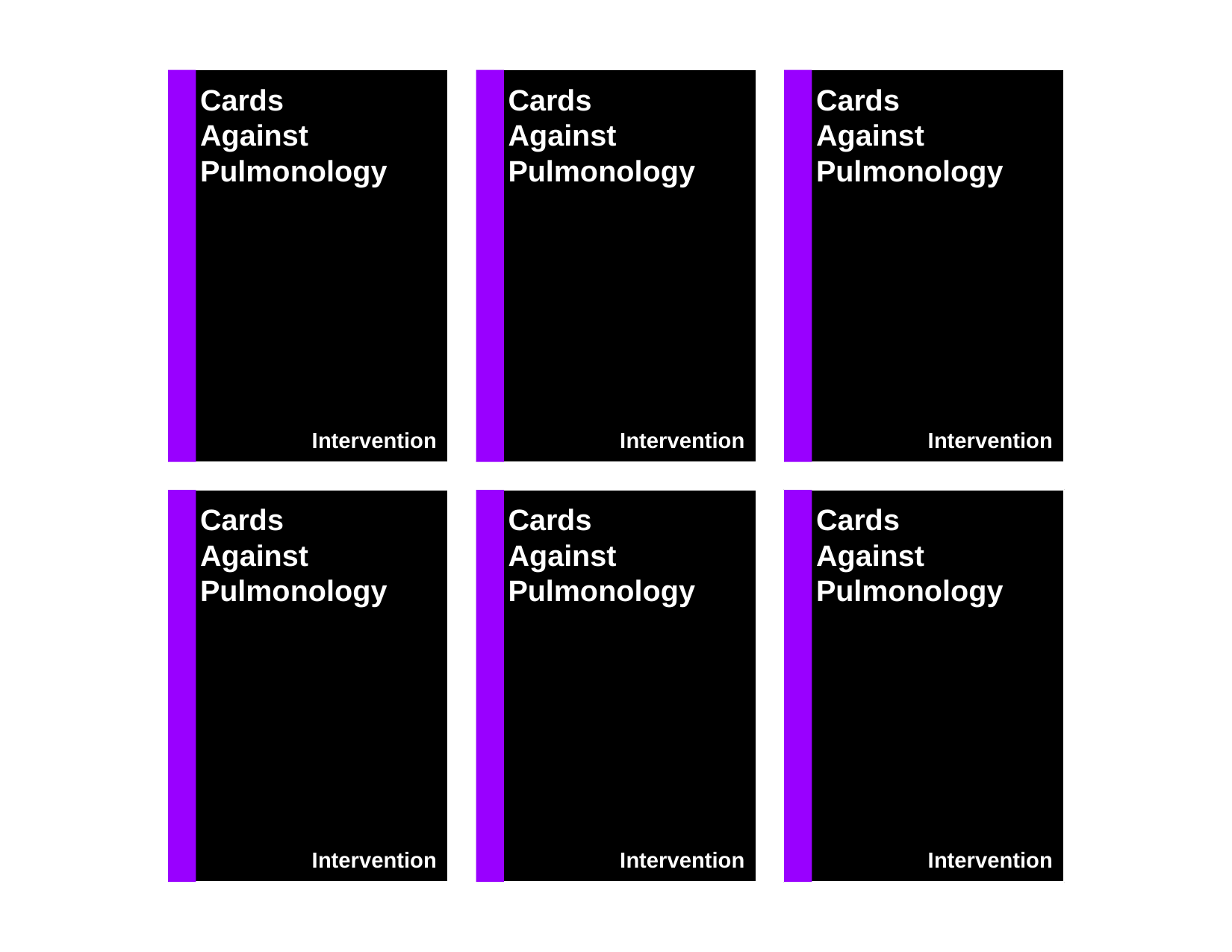

Cards
Against
Pulmonology
Intervention
Cards
Against
Pulmonology
Intervention
Cards
Against
Pulmonology
Intervention
Cards
Against
Pulmonology
Intervention
Cards
Against
Pulmonology
Intervention
Cards
Against
Pulmonology
Intervention

## Slide 61
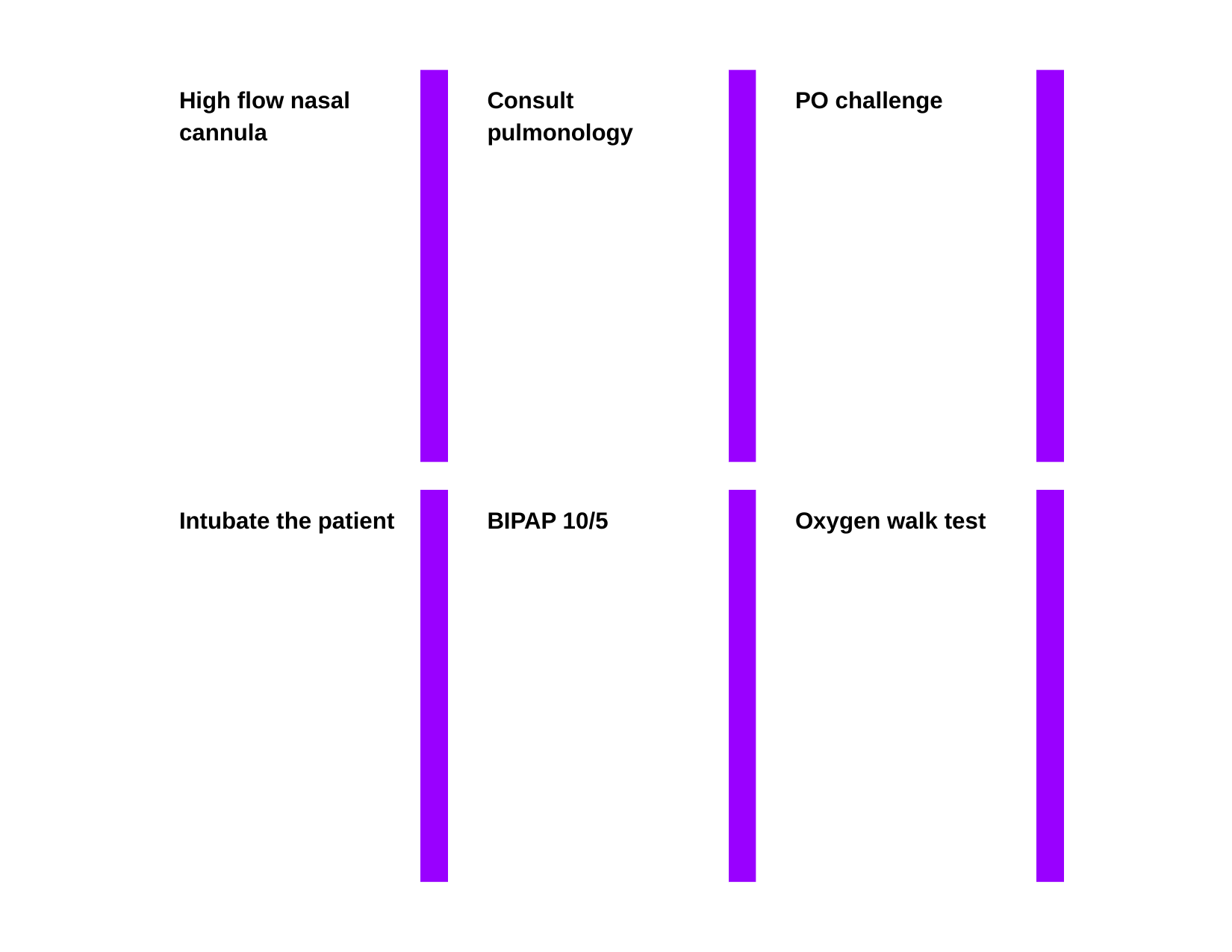

High flow nasal cannula
Consult pulmonology
PO challenge
Intubate the patient
BIPAP 10/5
Oxygen walk test

## Slide 62
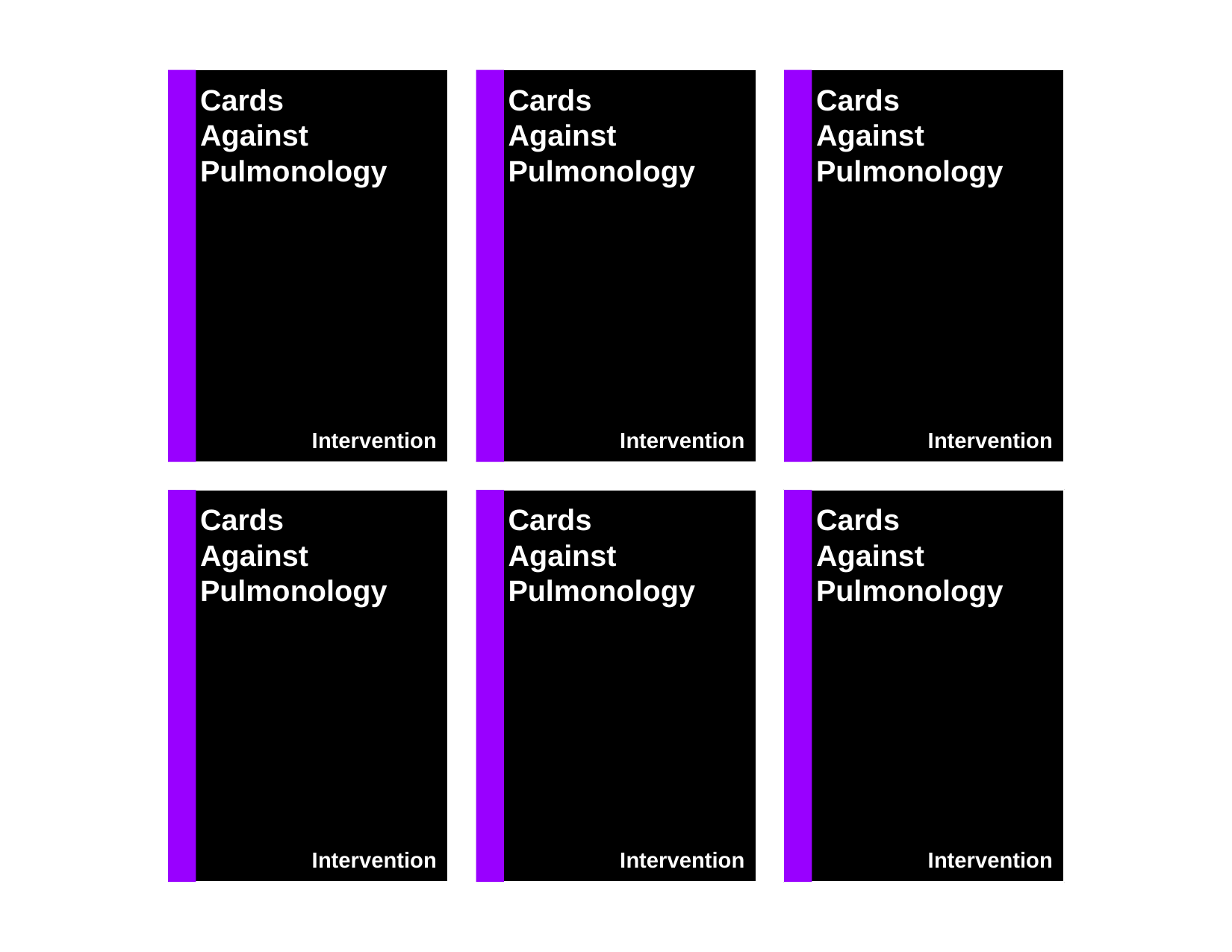

Cards
Against
Pulmonology
Intervention
Cards
Against
Pulmonology
Intervention
Cards
Against
Pulmonology
Intervention
Cards
Against
Pulmonology
Intervention
Cards
Against
Pulmonology
Intervention
Cards
Against
Pulmonology
Intervention

## Slide 63
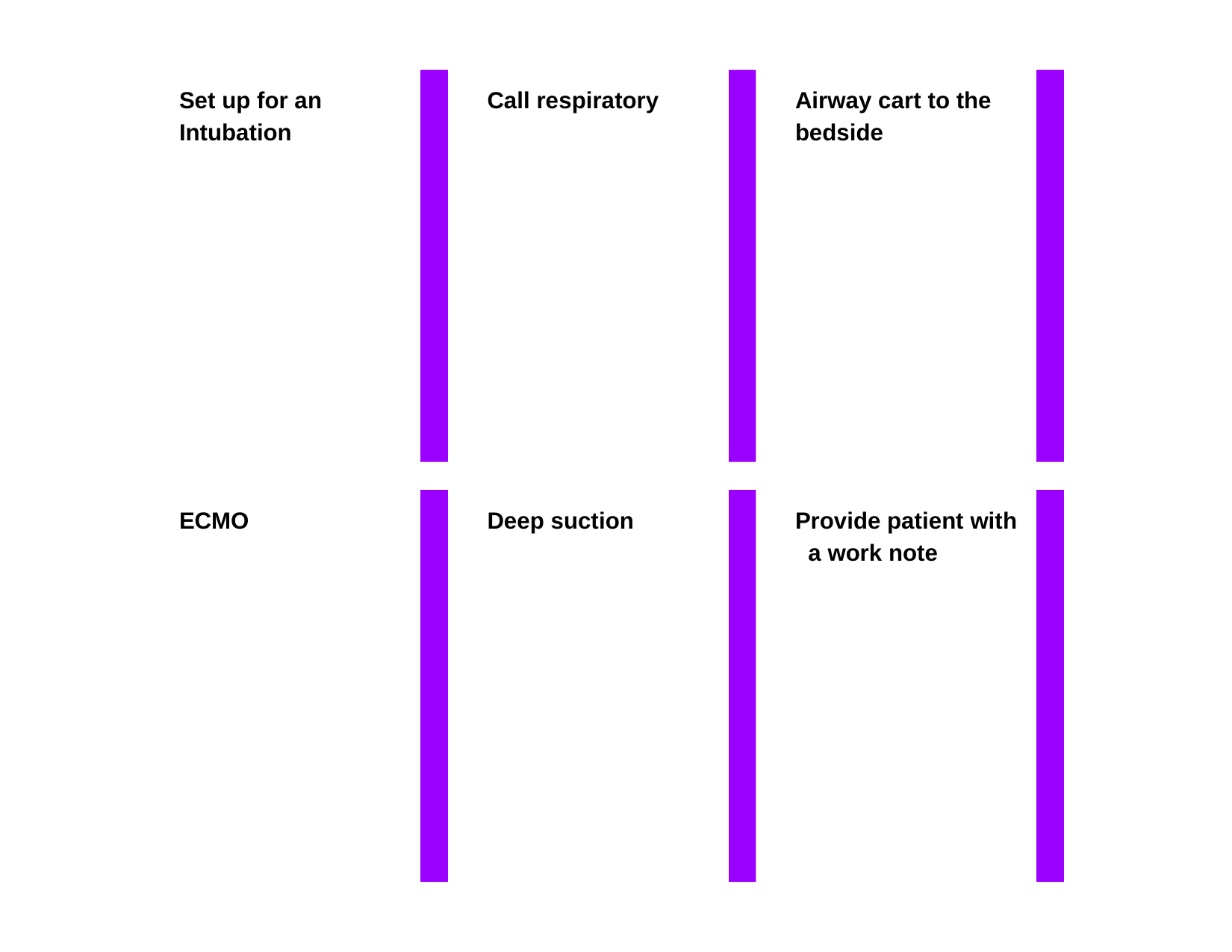

Set up for an Intubation
Call respiratory
Airway cart to the bedside
ECMO
Deep suction
Provide patient with a work note

## Slide 64
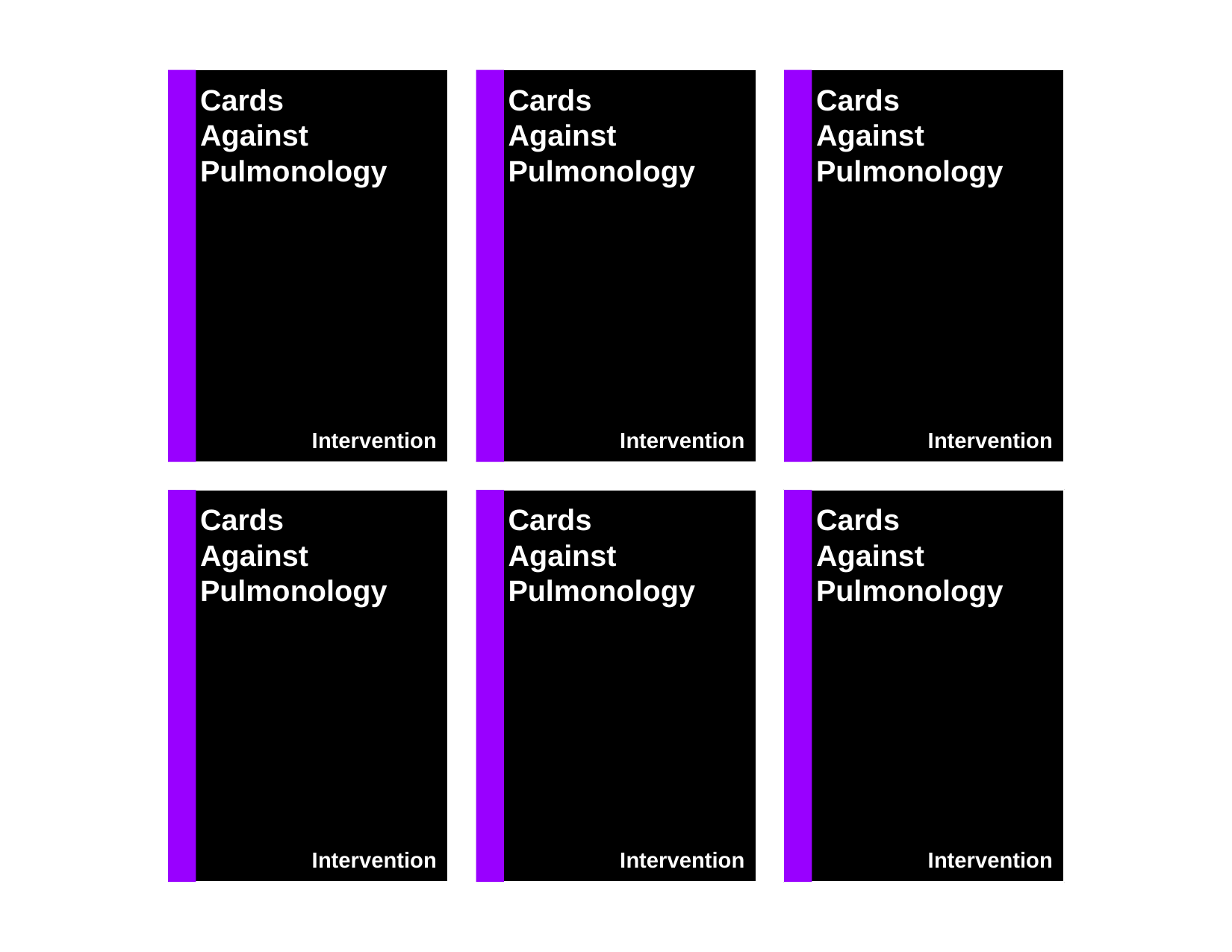

Cards
Against
Pulmonology
Intervention
Cards
Against
Pulmonology
Intervention
Cards
Against
Pulmonology
Intervention
Cards
Against
Pulmonology
Intervention
Cards
Against
Pulmonology
Intervention
Cards
Against
Pulmonology
Intervention

## Slide 65
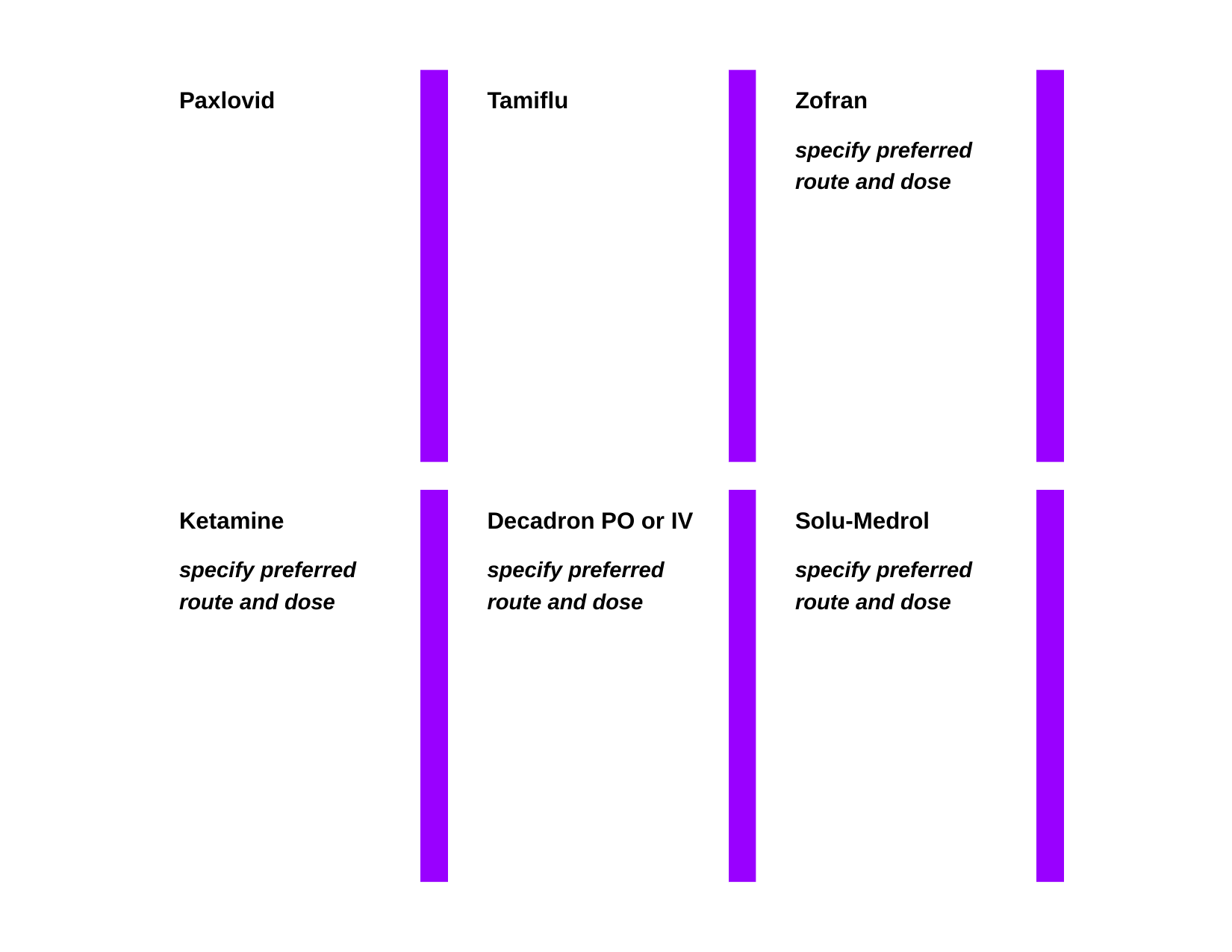

Paxlovid
Tamiflu
Zofran
specify preferred route and dose
Ketamine
specify preferred route and dose
Decadron PO or IV
specify preferred route and dose
Solu-Medrol
specify preferred route and dose

## Slide 66
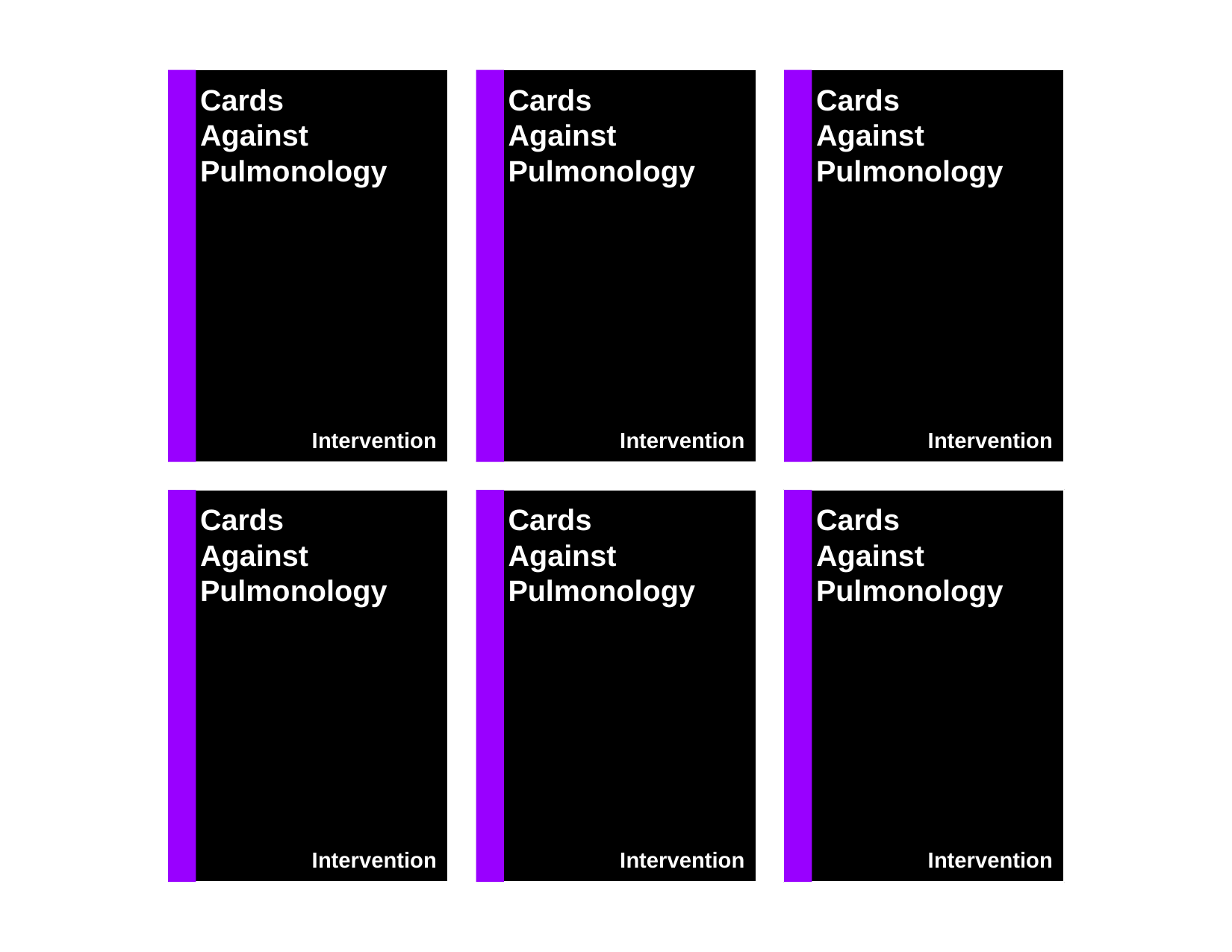

Cards
Against
Pulmonology
Intervention
Cards
Against
Pulmonology
Intervention
Cards
Against
Pulmonology
Intervention
Cards
Against
Pulmonology
Intervention
Cards
Against
Pulmonology
Intervention
Cards
Against
Pulmonology
Intervention

## Slide 67
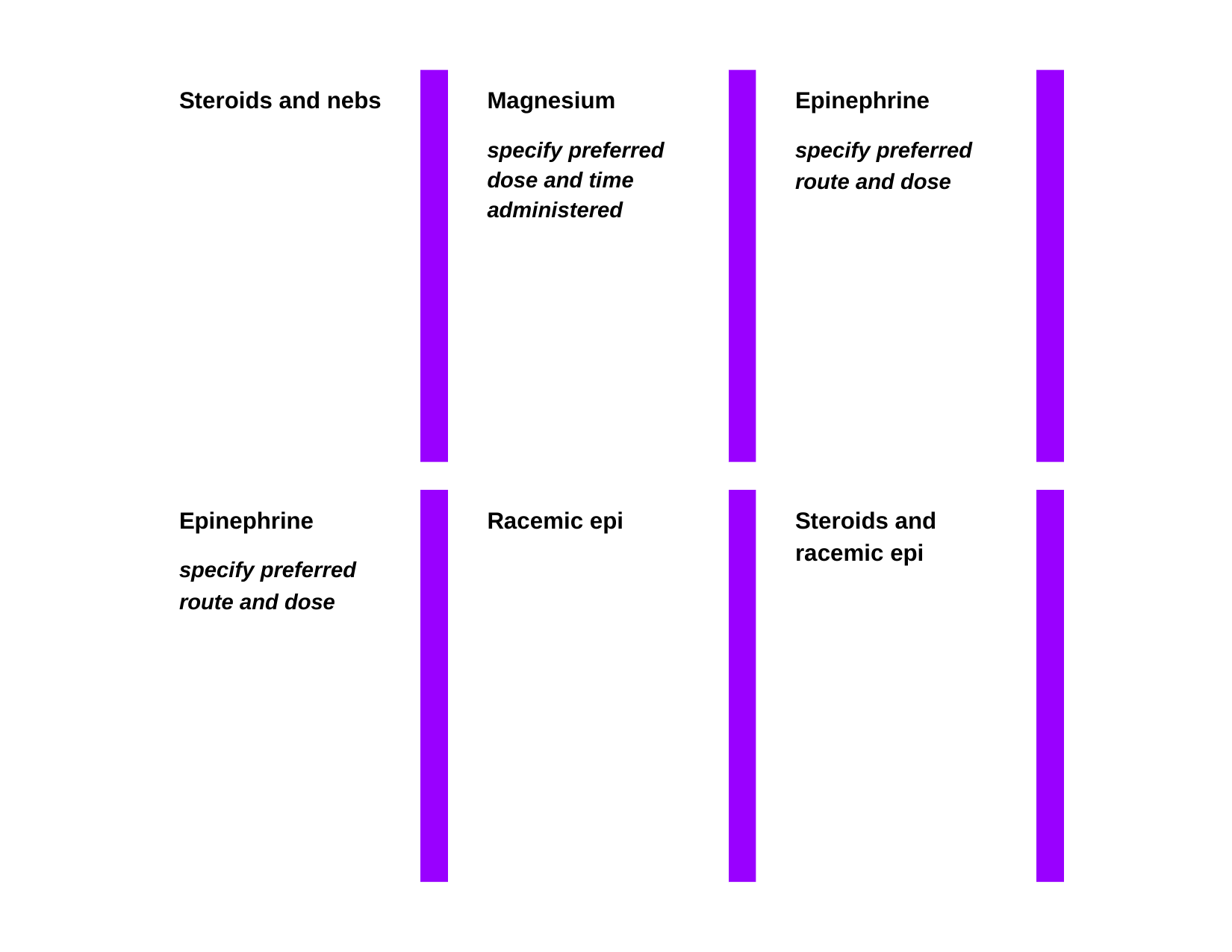

Steroids and nebs
Magnesium
specify preferred dose and time administered
Epinephrine
specify preferred route and dose
Epinephrine
specify preferred route and dose
Racemic epi
Steroids and racemic epi

## Slide 68
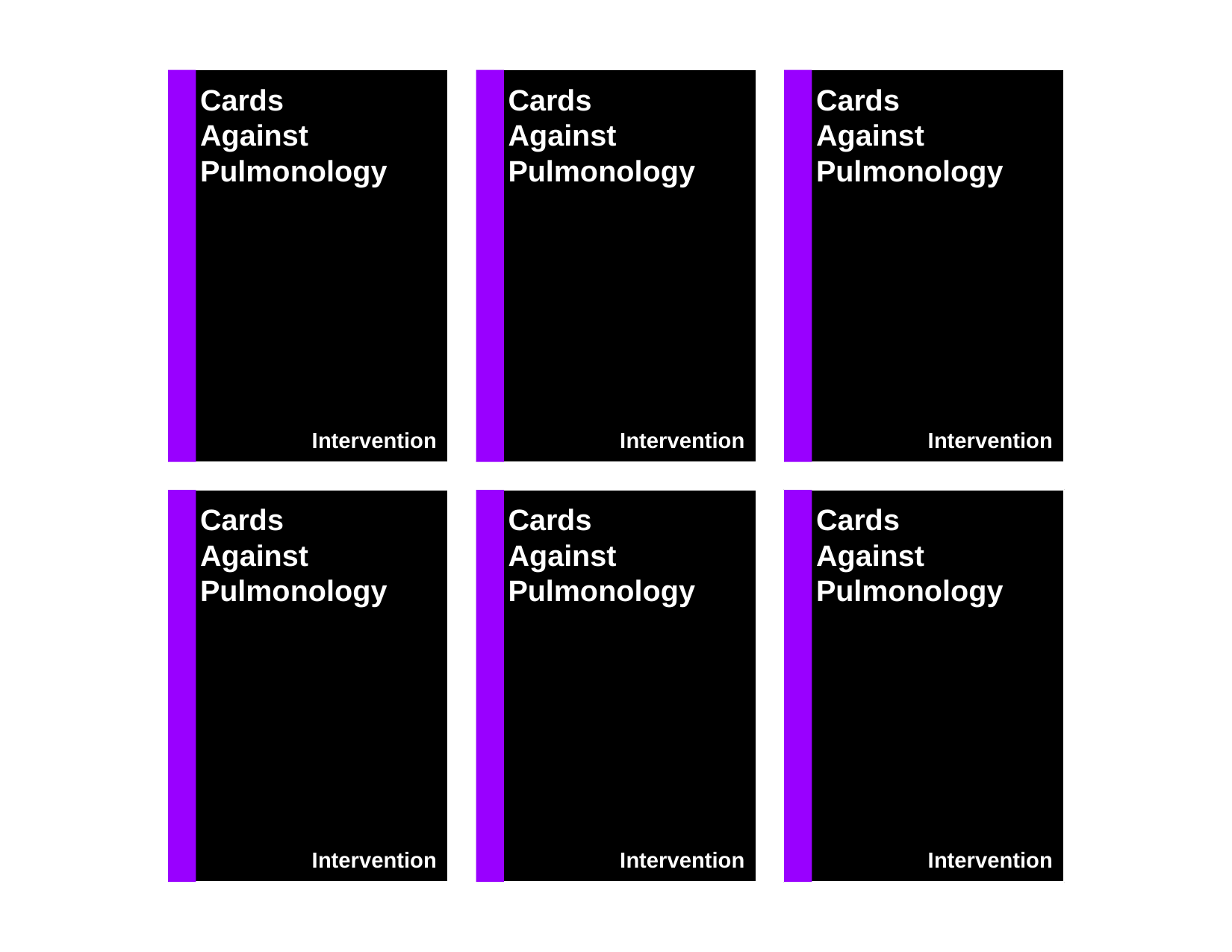

Cards
Against
Pulmonology
Intervention
Cards
Against
Pulmonology
Intervention
Cards
Against
Pulmonology
Intervention
Cards
Against
Pulmonology
Intervention
Cards
Against
Pulmonology
Intervention
Cards
Against
Pulmonology
Intervention

## Slide 69
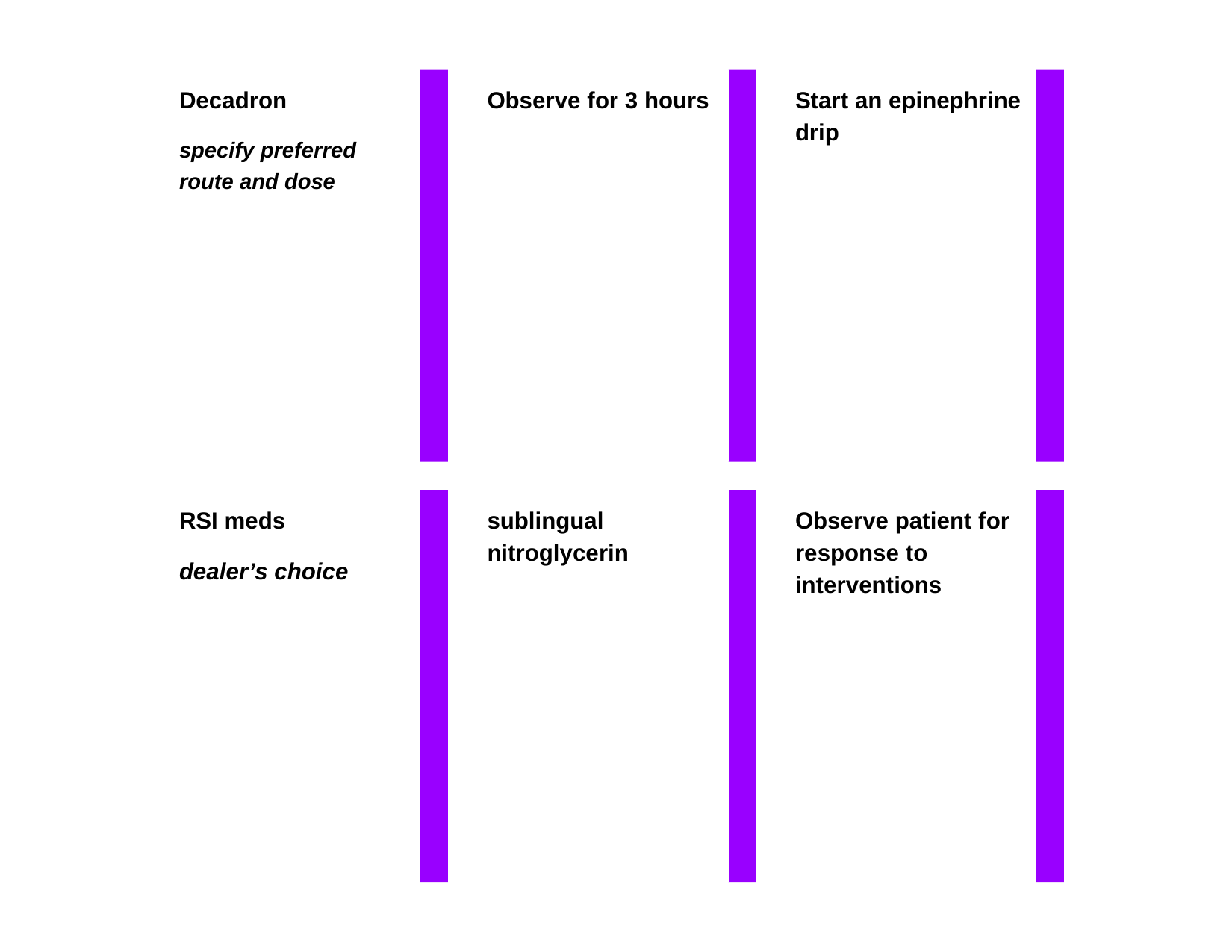

Decadron
specify preferred route and dose
Observe for 3 hours
Start an epinephrine drip
RSI meds
dealer’s choice
sublingual nitroglycerin
Observe patient for response to interventions

## Slide 70
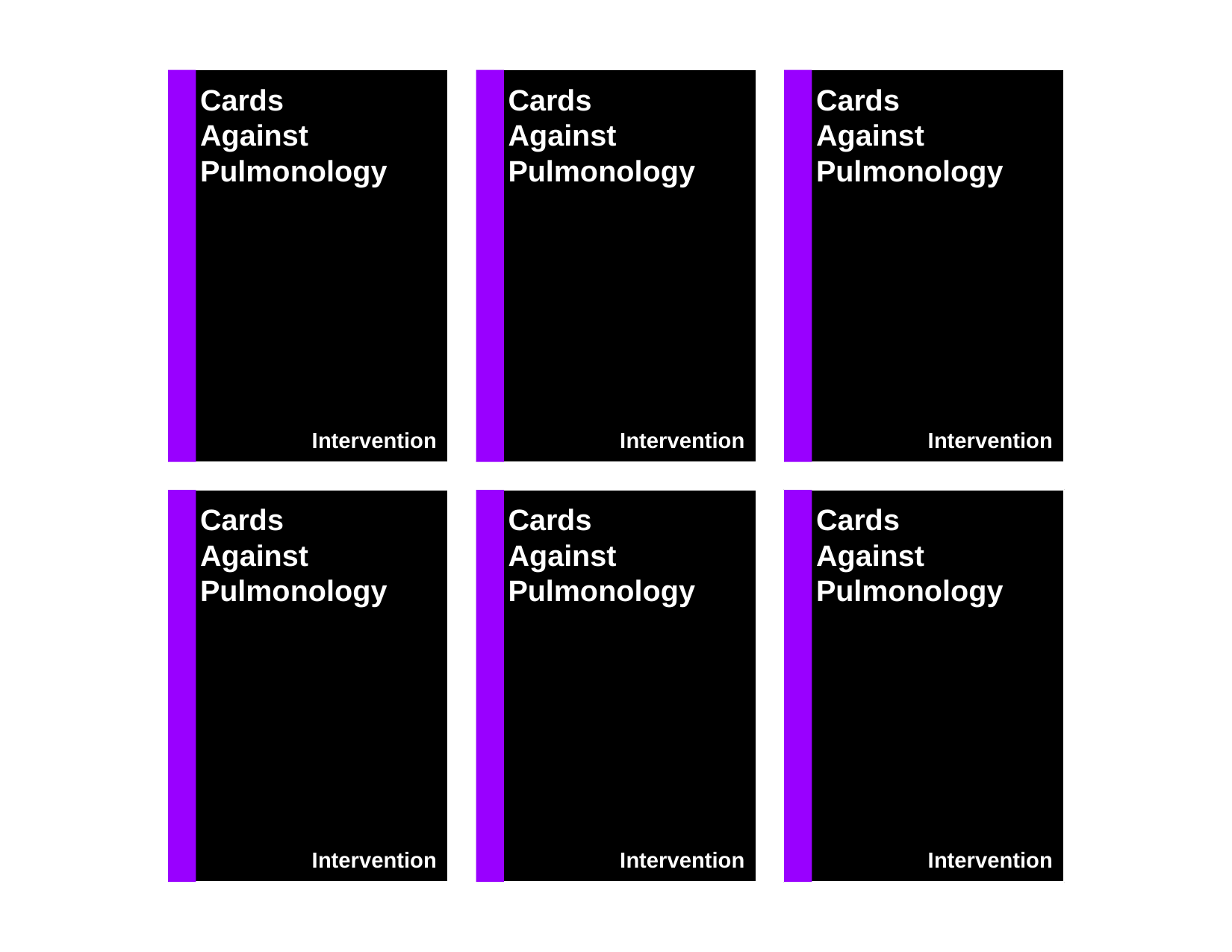

Cards
Against
Pulmonology
Intervention
Cards
Against
Pulmonology
Intervention
Cards
Against
Pulmonology
Intervention
Cards
Against
Pulmonology
Intervention
Cards
Against
Pulmonology
Intervention
Cards
Against
Pulmonology
Intervention

## Slide 71
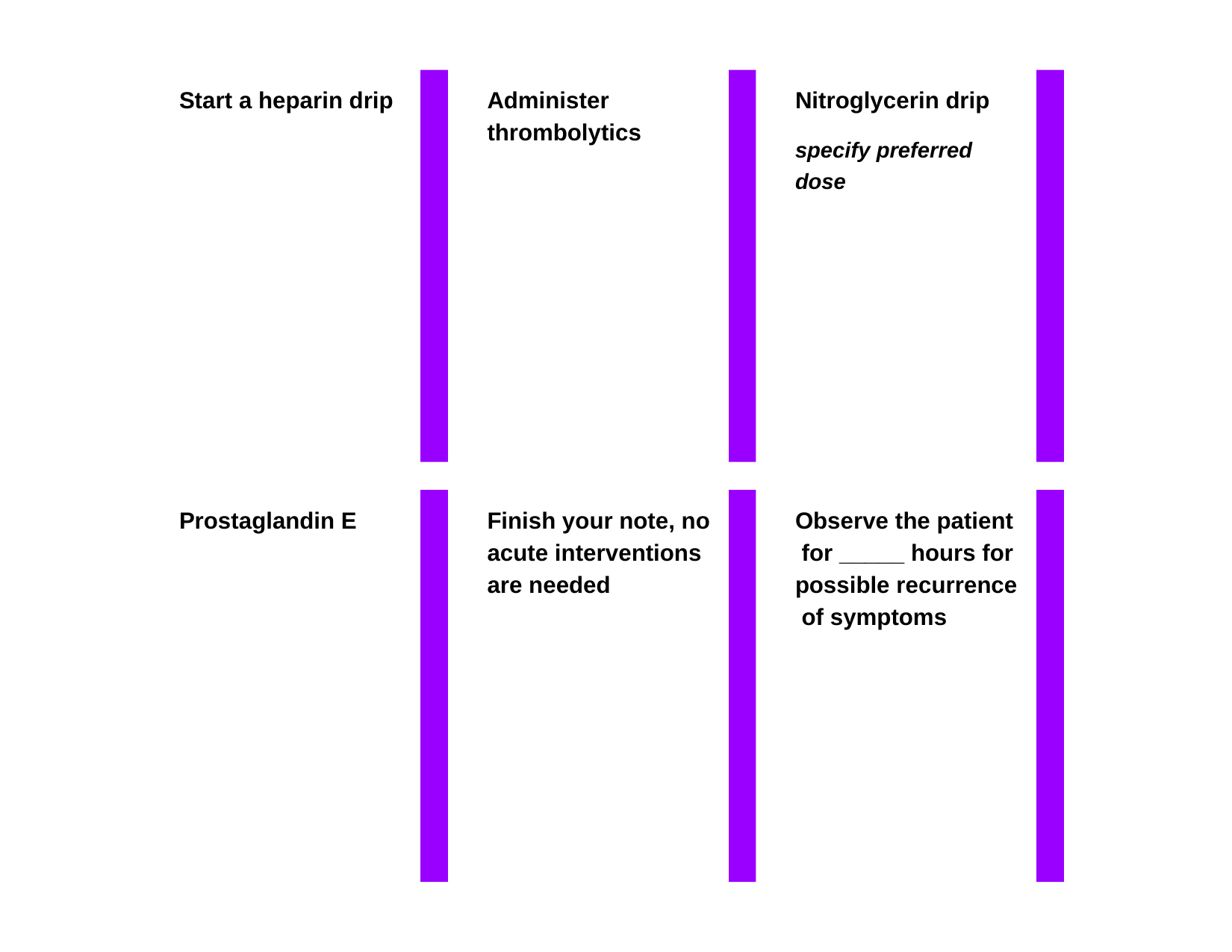

Start a heparin drip
Administer thrombolytics
Nitroglycerin drip
specify preferred dose
Prostaglandin E
Finish your note, no acute interventions are needed
Observe the patient for _____ hours for possible recurrence of symptoms

## Slide 72
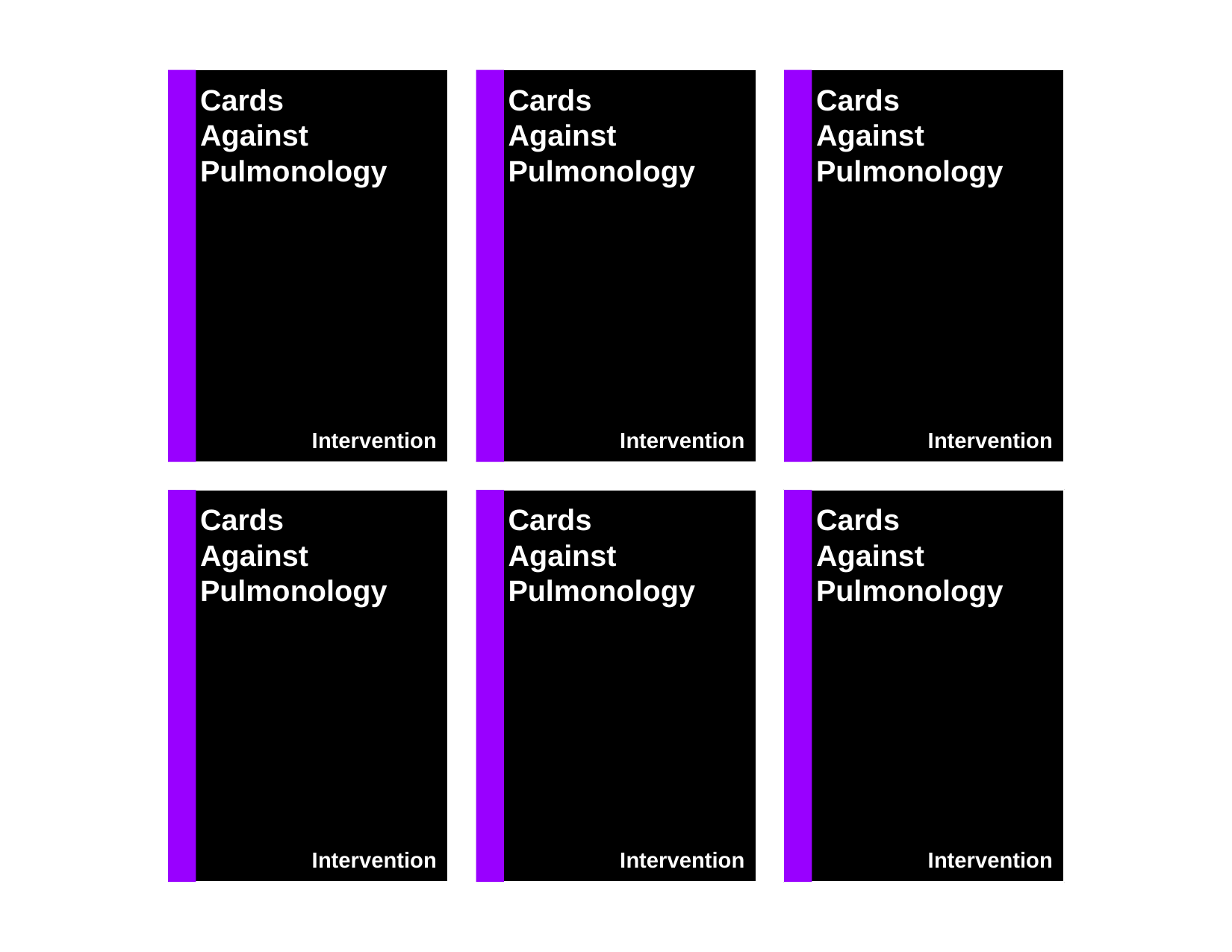

Cards
Against
Pulmonology
Intervention
Cards
Against
Pulmonology
Intervention
Cards
Against
Pulmonology
Intervention
Cards
Against
Pulmonology
Intervention
Cards
Against
Pulmonology
Intervention
Cards
Against
Pulmonology
Intervention

## Slide 73
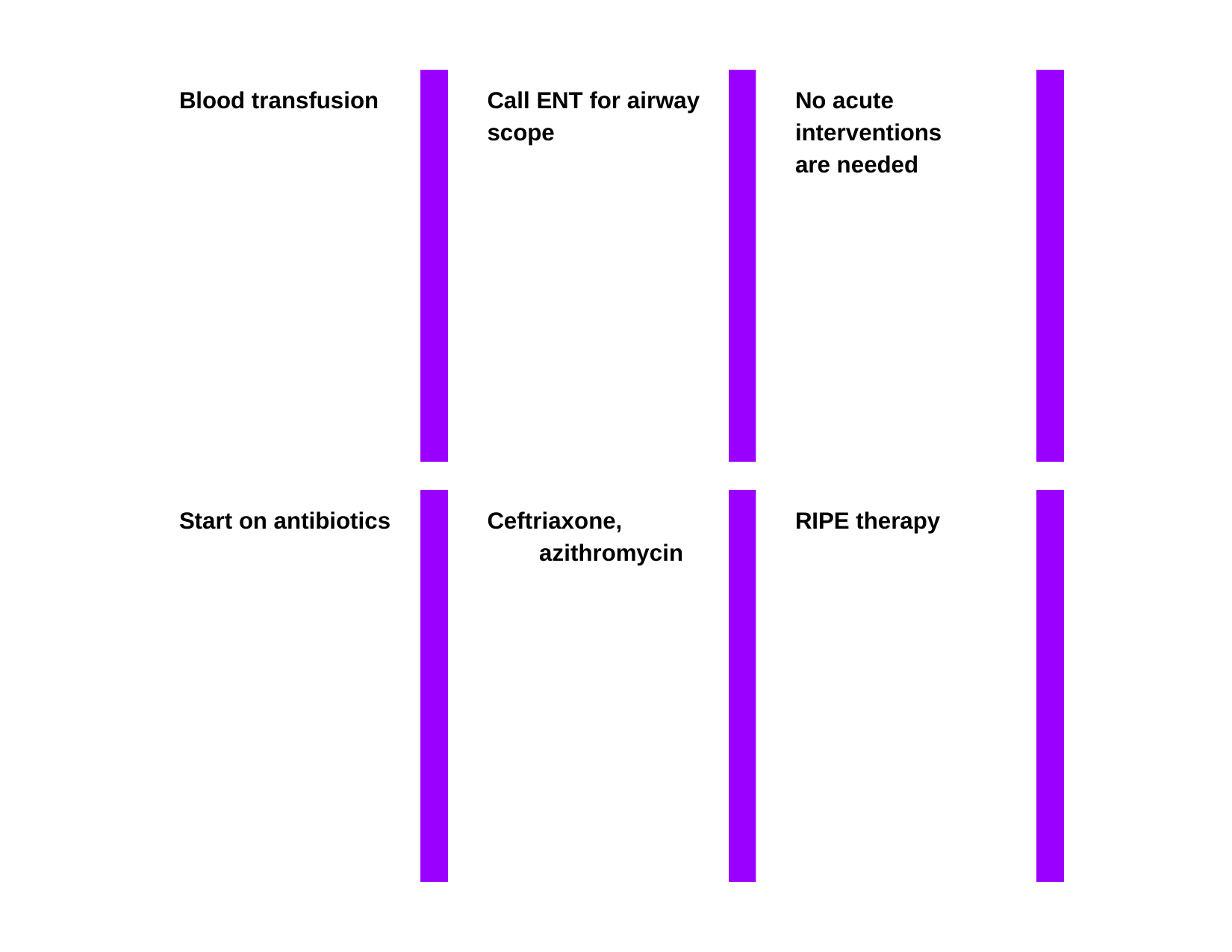

Blood transfusion
Call ENT for airway scope
No acute interventions are needed
Start on antibiotics
Ceftriaxone, azithromycin
RIPE therapy

## Slide 74
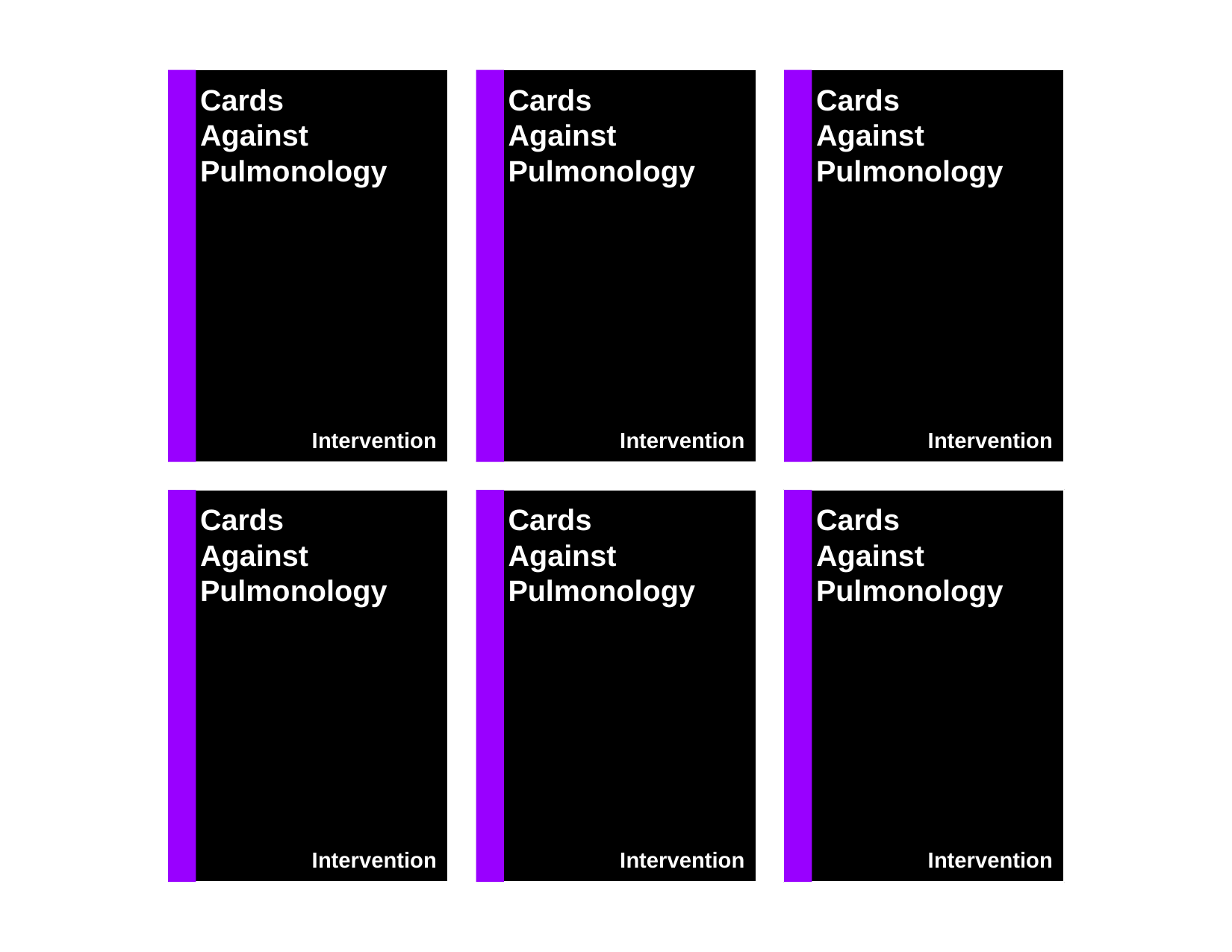

Cards
Against
Pulmonology
Intervention
Cards
Against
Pulmonology
Intervention
Cards
Against
Pulmonology
Intervention
Cards
Against
Pulmonology
Intervention
Cards
Against
Pulmonology
Intervention
Cards
Against
Pulmonology
Intervention

## Slide 75
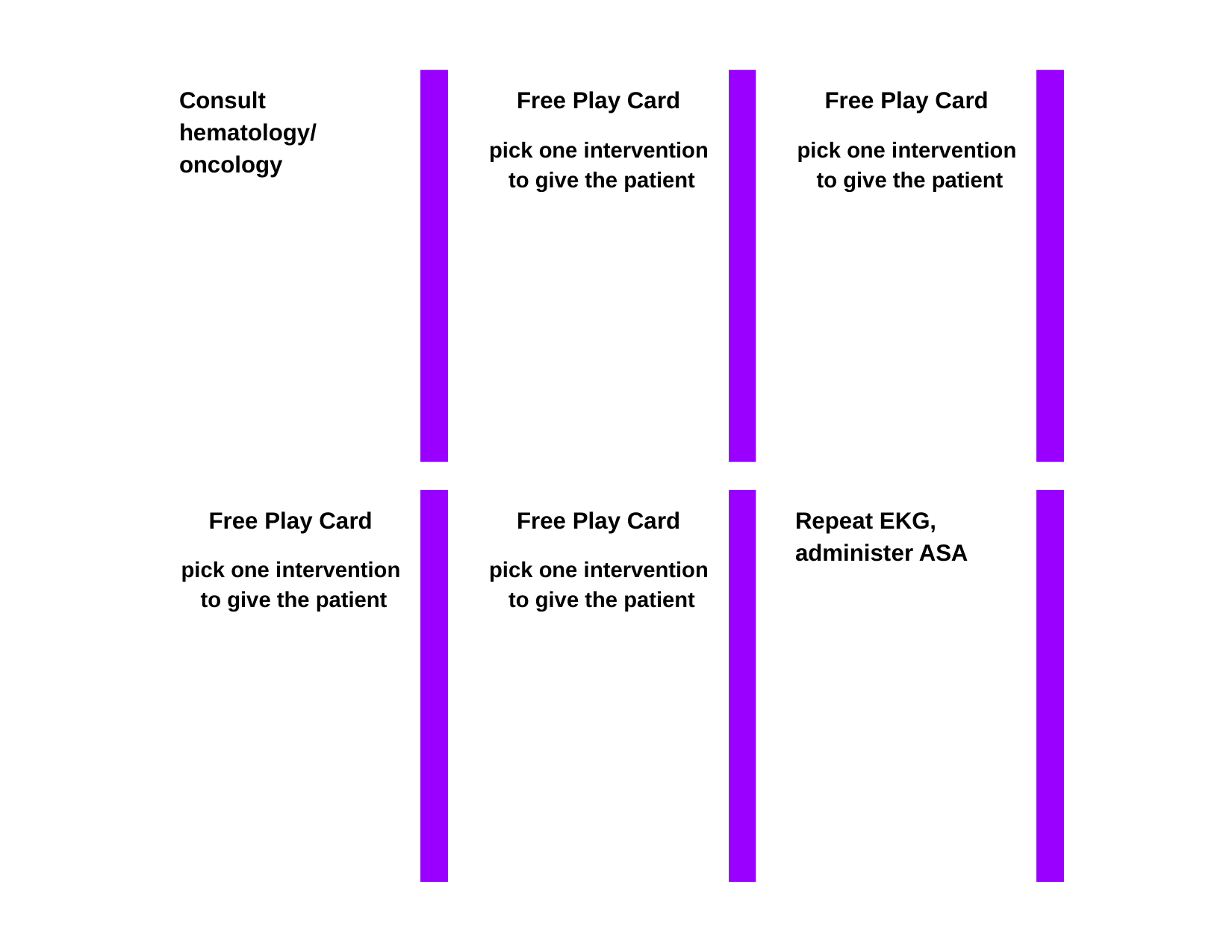

Consult hematology/ oncology
Free Play Card
pick one intervention to give the patient
Free Play Card
pick one intervention to give the patient
Free Play Card
pick one intervention to give the patient
Free Play Card
pick one intervention to give the patient
Repeat EKG, administer ASA
